# A life cycle risk assessment of nanopesticides in freshwater

**DOI:** 10.1016/j.ese.2025.100565

**Published:** 2025-05-02

**Authors:** Mingyan Ke, Keshuo Zhang, Andrea L. Hicks, Fan Wu, Jing You

**Affiliations:** a College of Environment and Climate, Guangdong Provincial Key Laboratory of Environmental Pollution and Health, Jinan University, Guangzhou, 511443, China; b Department of Civil and Environmental Engineering, University of Wisconsin-Madison, Madison, WI, 53706, USA

**Keywords:** Nanopesticide, Risk assessment, Life cycle assessment, Characterization factor, Impact score

## Abstract

Conventional ecological risk assessments prioritize downstream anthropogenic impacts, overlooking risks arising from upstream processes involving highly hazardous substances and indirect emissions. This narrow focus obscures high-risk hotspots and renders traditional methodologies ill-suited for evaluating novel chemical entities. Nanopesticides, designed for targeted delivery of pesticidal active ingredients, are increasingly deployed to enhance efficiency, yet their altered environmental fate and transport dynamics may reshape end-of-life risks while their full lifecycle impacts remain uncharacterized. Here, we address this gap using imidacloprid (IMI) and its nano-encapsulated variant (nano-IMI) as case studies. By applying life cycle assessment and integrating the USEtox ecotoxicity model with the nano-specific SimpleBox4Nano framework, we quantify "cradle-to-gate" environmental impacts and derive substance-specific ecotoxicity metrics, enabling systematic characterization of end-of-life risks associated with these formulations. Production-stage ecological risks of nano-IMI (4.63 × 10^3^ CTUe) are approximately four times greater than those for conventional IMI (1.18 × 10^3^ CTUe). However, end-of-life freshwater ecological risks from nano-IMI emissions (0.012–6.93 × 10^4^ CTUe) are 2–5 orders of magnitude lower compared with IMI (1.59 × 10^3^–6.13 × 10^6^ CTUe), accounting for rainfall variability, toxicity data selection, fate, and environmental transport scenarios. Under equivalent rainfall conditions, nano-IMI exhibited up to three orders of magnitude lower integrated life-cycle freshwater ecological risks, underscoring its potential as an environmentally preferable alternative to conventional IMI. This research introduces a comprehensive and novel methodology for evaluating engineered nanomaterial alternatives across realistic environmental scenarios, providing essential insights into nanopesticide risk assessment throughout their lifecycle.

## Highlights

• Life-cycle freshwater risks of conventional/nano-imidacloprid (IMI/nano-IMI) exhibit stage-specific disparities.
• Production-stage risk of nano-IMI is four times higher than IMI due to intensive nano-synthesis.
• End-of-life risks for nano-IMI are 2–5 orders of magnitudes lower than IMI under various scenarios.
• Nano-IMI reduces cumulative life-cycle freshwater risk by up to three orders of magnitude.
• The integrated framework provides a quantitative guide for designing greener chemicals.

## Graphical abstract

**Figure:**
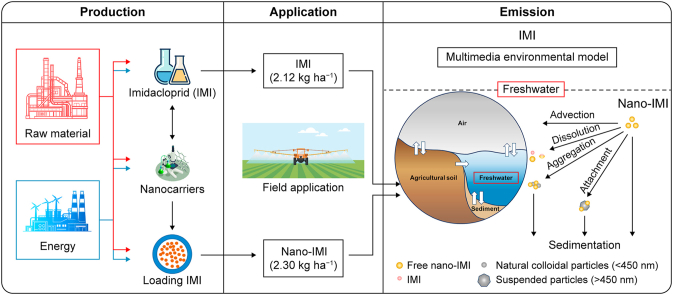

## 1 Introduction

The rapid growth in the global population and escalating food demand have led to increased use of pesticides in agricultural activities, resulting in significant environmental tradeoffs [1]. Nanotechnology-enabled pesticides represent an important measure for increasing pesticide efficiency while potentially decreasing harmful environmental consequences by facilitating active components with high stability, improved effectiveness, and targeted release against organisms compared to their conventional counterparts [2,3].

Nanopesticides can potentially reduce ecological threats due to their altered fate and transport behaviors after being applied in the field [[4], [5], [6]]. However, to date, few studies have demonstrated the adverse end-of-life outcomes nanopesticides can have on non-target organisms once they are released, such as impacting plant photosynthesis [7], causing larval mortality and biochemical modifications in midges [8] and leading to deformities and fatalities in embryonic fish [9,10]. In addition, there is a lack of information on the differences in toxicity between nanopesticides and conventional pesticides [11].

More importantly, the environmental impacts of nanopesticides cascade across their entire life cycle [1]. However, the tradeoffs between ecosystem exposure to nanopesticides and the impacts associated with the chemical synthesis procedures throughout the products’ life cycles have not been thoroughly considered [[12], [13], [14]]. The global nanopesticides market, valued at $735 million in 2024, is projected to increase to around $2.08 billion by 2032, demonstrating a compound annual growth rate (CAGR) of 13.9 % between 2024 and 2032 [15]. With the continuous expansion of the nanopesticides market, there is an urgent need for regulatory and scientific scrutiny of these products.

Life cycle assessment (LCA) offers a comprehensive evaluation of the environmental impacts of every stage of a product's life, from raw material extraction and production to its use and disposal [16]. However, current cradle-to-grave LCAs for nano-enabled products are challenging due to their unique nano-properties, making them challenging to compare with conventional products. Specifically, studies have indicated that a lack of characterization factors (*CFs*) for engineered nanomaterials (ENMs) is key to the high uncertainty regarding their environmental impacts [[17], [18], [19]].

The USEtox model provides a structured approach for assessing human toxicity and ecotoxicity in LCA and other comparative frameworks. It rigorously documents the impact pathways of chemical emissions by analyzing their environmental distribution, fate, and exposure routes for humans and ecosystems and associated toxic effects. These factors constitute the midpoint characterization factors in LCA [20]. The USEtox model has been extensively used to complement LCA to quantify the (eco)toxicity *CF*s for various organic and inorganic materials.

Previous studies have integrated *CFs* and LCAs to comprehensively illustrate the complete overall risks of chemicals within certain industries, such as dyeing media [21], microplastics [22], pharmaceutical and personal care products [23], and shale gas exploitation [24]. However, nanomaterials differ from conventional chemicals. Owing to the absence of inventory data for *CF*s and nano-specific physicochemical properties, it is challenging to quantify the ecotoxicity *CFs* of nanopesticides. Therefore, current LCA studies seldom consider nano-specific emissions when determining the environmental impacts of nanopesticides [25]. Moreover, directly applying the USEtox model to calculate the *CFs* of nanomaterials may introduce potential biases when understanding potential risks. Characterization factors should, therefore, be incorporated with predicted environmental concentrations (PECs) considering the fate of chemicals to derive an impact score (*IS*) to illustrate the end-of-life risks of such chemicals [20]. SimpleBox and SimpleBox4Nano (SB4N) are two multimedia environmental fate models developed to simulate the fate and exposure of typical substances and nanomaterials in the environment [26]. SB4N can be effectively integrated with the USEtox model to capture the specific environmental fate of nanoparticles accurately and has proven effective in deriving PECs for metallic nanomaterials, such as nano-TiO_2_ [27], nano-Ag [28], and nano-Cu [29]. This hybrid architecture enables the simultaneous tracking of the fate of engineered nanoparticles (ENPs), such as aggregation and dissolution status, while revealing potential ecological risks under different environmental conditions.

A thorough LCA is urgently needed as the first line of defense to ensure environmental safety and determine whether nanopesticides represent a safer alternative to conventional pesticides before their widespread use [30,31]. Imidacloprid (IMI) is among the most extensively used neonicotinoid insecticides and functions as an agonist for nicotinic acetylcholine receptors (nAChRs), disrupting neurotransmission in the central nervous systems of invertebrates [32]. Imidacloprid has been widely detected in surface water up to ppm levels [33]. To minimize the potential adverse effects of conventional IMI, nano-encapsulated imidacloprid (nano-IMI) was developed and registered for use in North America and has exhibited increased efficacy [34], an extended efficacy period [35], and enhanced photodegradation performance [36].

To systematically reveal the ecological risks and substitution potential of nano-IMI to conventional IMI, the present study performed an LCA to evaluate and compare the “cradle-to-gate” environmental impacts of IMI and nano-IMI production. Furthermore, the end-of-life ecotoxicity *IS* of IMI and nano-IMI were estimated by incorporating USEtox and SimpleBox/SB4N, complementing the LCA results. Our approach systematically reveals the ecological risks of nanopesticides from production to end-of-life emissions and facilitates a comparative analysis of the relative impacts of both pesticides. The integrated results can promote the sound management of nano-agrochemicals and are important for minimizing the adverse impacts of nano-agrochemicals for implementing the 2030 Agenda for Sustainable Development.

## 2 Methods

### 2.1 Life cycle assessments of IMI and nano-IMI production

**Fig. 1:**
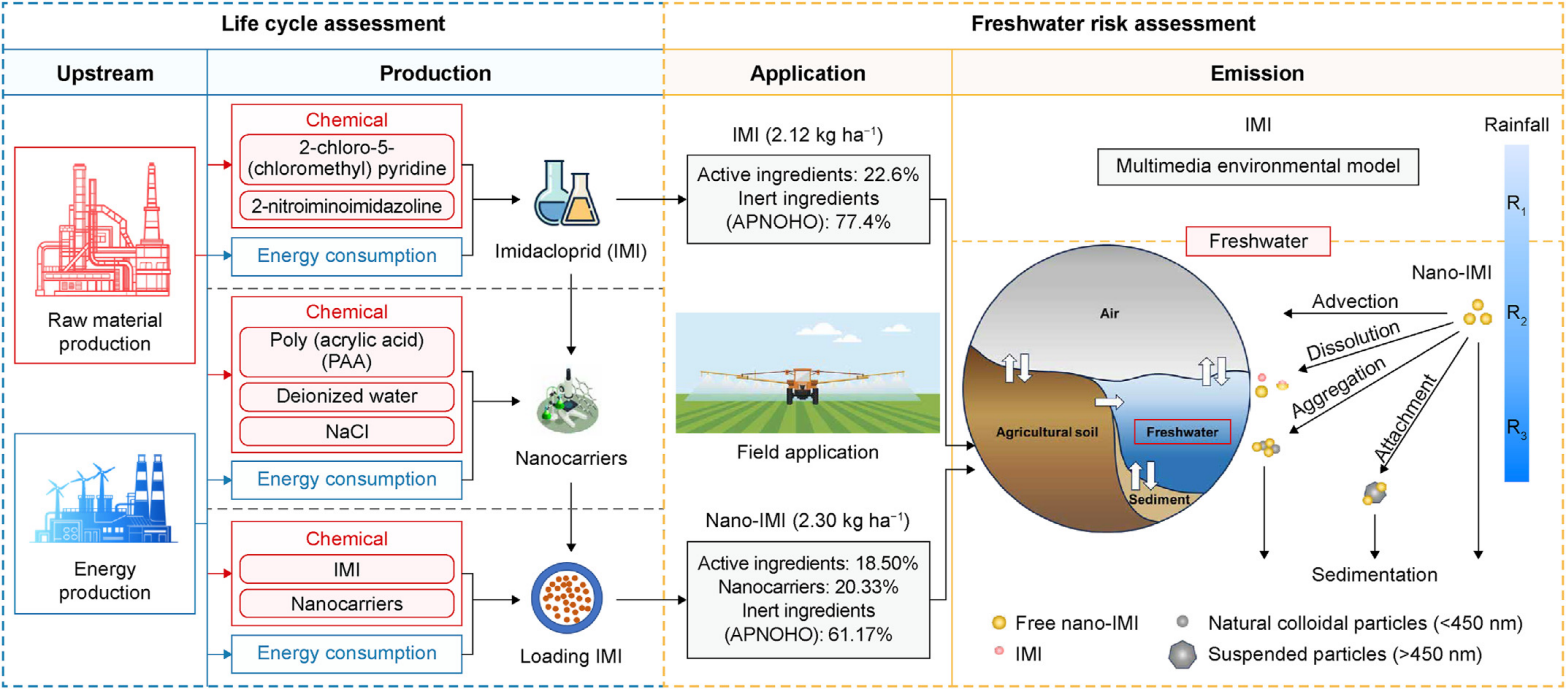
System boundaries considering the life cycle impacts during pesticide production and the end-of-life freshwater risks. R_1_, R_2_, and R_3_ represent limited, normal, and heavy rainfall scenarios, respectively.

***Goal and scope.*** The framework illustrating the overall freshwater risks of IMI and nano-IMI is depicted in Fig. 1. A process-based LCA was initially conducted to quantitatively elucidate the corresponding environmental impacts of nano-IMI/IMI production and identify environmental hotspots. Our initial impact assessment employed mass-based functional units (1 kg of pesticide production). Additionally, the annual per-hectare quantity of pesticide applied was used to rescale environmental impacts (2.30 and 2.12 kg ha^−1^ for nano-IMI and IMI, respectively) [37,38]. The system boundary encompassed the raw material extraction and the production stage for IMI and nano-IMI (Fig. 1). Given that the nanopesticide utilized in this study was manufactured in Canada, the tool for the reduction and assessment of chemical and other environmental impacts (TRACI 2.1) was selected as the impact assessment method to align with North American regulatory frameworks, while regionalized life cycle inventory (LCI) data were sourced from the ecoinvent V3.8 North American datasets. This regional approach establishes a transferable methodological paradigm, providing actionable insights for other jurisdictions developing nano-agrochemical regulations.

***Life cycle inventories*.** The active ingredient (IMI) of nano-IMI was encapsulated through the polymer nanocarrier Allosperse® delivery system (polyacrylic acid, PAA), allowing IMI to have elevated stability and an extended residence period and to be readily used along with other plant protection substances [38]. The respective LCIs for IMI and nano-IMI production were compiled from patents and scientific publications (Supplementary Material Text S1 and Table S1–S18). The detailed synthesis and encapsulation procedures for each pesticide are depicted in Fig. S1–S16 (Supplementary Material). Briefly, IMI and polyacrylic acid nano-capsules were dissolved in a methanol solvent, and the active ingredients IMI can be encapsulated by adjusting the pH of the solvent. Subsequently, the methanol solvent was removed, and the nano-IMI particles were recovered through evaporation and freeze-drying, respectively. The conventional IMI formula comprises 22.6 % active ingredients and 77.4 % unspecified inert adjuvant. The encapsulation rate of PAA nanocarriers in the nano-IMI was up to 91 % [39], resulting in the theoretical composition of 18.5 % IMI, 20.3 % nanocarriers, and 61.2 % unspecified inert adjuvant for the nano-IMI formulation (Supplementary Material Table S17) [40]. The preliminary findings indicate that no significant toxicity was observed in nano-capsules or other adjuvant additives of nano-IMI [9].

Assumptions**.** The present study assumed that the inert component of both IMI and nano-IMI is APNOHO (Chemical abstracts service registry number: 9016-45-9), enabling an equivalent comparison between these two pesticides [40]. The amount of IMI utilized in nano-IMI encapsulation surpasses the loading capacity of the nano-carrier, thus necessitating IMI recovery, which was included in the system boundary for the nano-IMI synthesis. Byproducts generated were emitted to the “technosphere” as avoided products. Furthermore, it was assumed that both IMI and nano-IMI had been manufactured within a closed reactor, thereby eliminating the possibility of their release during the production phase. The life cycle environmental impacts were modeled using SimaPro software with ecoinvent as the main database.

### 2.2 Multimedia environmental model

SimpleBox and SB4N were employed to assess the distribution of IMI and nano-IMI across multimedia environments, specifically air, freshwater, agricultural soil, and sediment, under steady-state conditions. These models facilitate the determination of steady-state PECs for IMI and nano-IMI by computing the steady-state solution of the mass balance matrices for these substances in different environmental compartments [20]. SB4N was specifically tailored to estimate environmental concentrations of ENPs in various states (e.g., free, aggregated, and attached) under steady-state conditions based on their physicochemical properties [41]. Emissions of IMI and nano-IMI in each environmental compartment were determined through a mass balance approach in matrices equations (1), (2). Subsequently, the PECs and emission fractions for both compounds were calculated:

(1) $$m_{\text{IMI}}=-{\bar{K}}^{-1}\times e_{\text{IMI}}$$

(2) $$m_{\text{nano}-\text{IMI}}=-{\bar{I}}^{-1}\times e_{\text{nano}-\text{IMI}}$$

where vector ***m*** represents the mass (g) of pesticides emitted to various environmental compartments, and the vector ***e*** denotes the emission rate (g s^−1^) of pesticides in those compartments, calculated based on the total annual soil application (1.07 × 10^8^ and 1.99 × 10^8^ kg y^−1^ for IMI and nano-IMI, respectively) (Supplementary Material Table S22). Matrices $\bar{K}$ and $\bar{I}$ correspond to the first-order rate constant (s^−1^) matrix of the transport and removal processes engaged with IMI and nano-IMI, all considered at a continental scale. All associated parameters are summarized in Sheet 4 (Supplementary Data 1 and 2). It is assumed that IMI or nano-IMI was applied through agricultural soils; thus, the soil was considered an emission source. At the same time, recognizing that precipitation can significantly affect the multimedia distribution of pesticides, rainfall data were collected from the World Bank Climate Portal to represent three rainfall scenarios: limited rainfall (R_1_: 18.1 mm y^−1^), normal rainfall (R_2_: 700 mm y^−1^, default value in the model), and heavy rainfall (R_3_: 3.04 × 10^3^ mm y^−1^) (Supplementary Data 1 Sheet 2). Detailed input parameters and calculations for the transport and removal processes of IMI (matrix $\bar{K}$) are provided in Supplementary Data 1 (Sheets 2–4 and 6–9).

Due to the specific physicochemical properties of nano-IMI, including the processes of aggregation with natural colloids or suspended particles, the dissolution rate constant (release rate of active ingredients), and the behavior of nano-IMI in freshwater environments involving deposition and advection [42], two scenarios assessed different hetero-aggregation behaviors of nano-IMI in the freshwater compartment. For scenario 1 (S1), hetero-aggregation is regarded as a clearing process, meaning the aggregated particles are removed from the system. Scenario 2 (S2) considers hetero-aggregation as a transformation process, where aggregated particles remain in the system and are gradually settled to the sedimentation [43]. Previous studies suggest that the environmental concentration of free ENP in freshwater is relatively low, and hetero-aggregation is much more likely to occur than homo-aggregation; hence, homo-aggregation was ignored during the process [44]. Moreover, as the attachment coefficient is a sensitive parameter for the SB4N model to the PECs [45], three attachment coefficients were considered in the present study, including the attachment coefficient obtained from previous literature (α_1_) [41], the attachment coefficient based on the SB4N model user guideline (α_2_) [26], and the default coefficient in the SB4N model (α_3_) (Supplementary Data 2 Sheets 2–4 and 6–10). Two aggregation scenarios, three α, and three rainfall scenarios were considered to capture all possible multimedia distributions of nano-IMI in freshwater. Historical measured environmental concentrations (MECs) of IMI were compared with PECs to validate the robustness of the theoretical model (Supplementary Material Text S2).

### 2.3 Ecotoxicity characterization factors

The USEtox model provides freshwater ecotoxicological impacts of chemical emissions conceiving environmental compartments as well-mixed boxes and enhances the accuracy of life cycle impact assessment (LCIA). It integrates fate, exposure, and effect to quantitatively analyze the cause–effect relationship of contaminants, resulting in low inter-model variations [46]. The characterization factor (*CF*) was derived using equation (3).

**Fig. 2:**
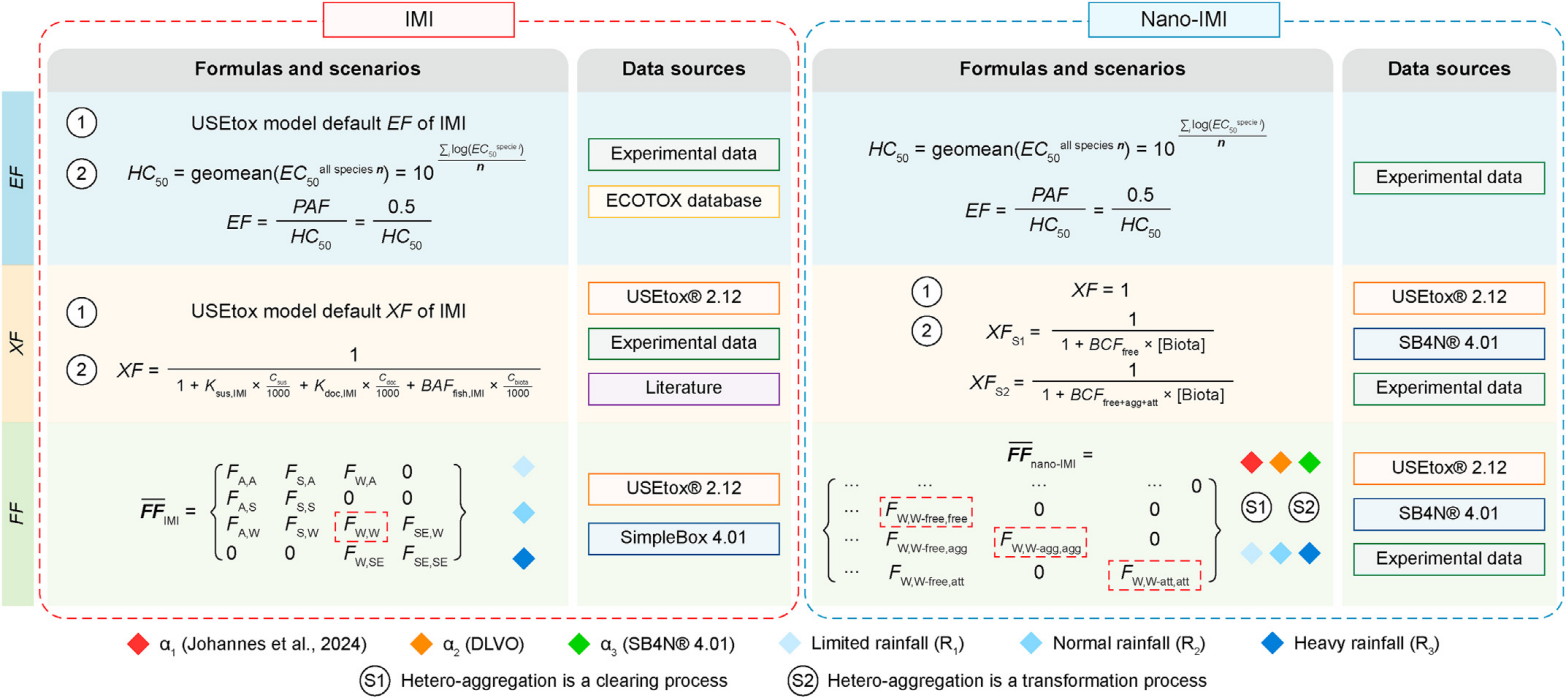
Summary of different models, formulas, and data sources used for deriving the freshwater characterization factors (*CF*s) of imidacloprid (IMI) and nano-IMI. The effect factor (*EF*), exposure factor (*XF*), and fate factor (*FF*) indicate the toxicity, bioavailability, and residence time of a substance in the environment, respectively. α_1_, α_2_, α_3_ are three attachment coefficients considered. SB4N: SimpleBox4Nano model. DLVO: Derjaguin, Landau, Verwey, and Overbeek theory. The parameters of the formulas in the figure are detailed in Supplementary Material Text S3.

(3) CF=EF×FF×XF

where effect factors (*EF*) signify toxicological impacts in terms of the potentially affected fraction of species (PAF) over a volume per mass of exposed substances (PAF m^3^ per kg emission). Fate factors (*FF*) denote the contaminant residence time (d), while exposure factors (*XF*) indicate the accessibility of the contaminant to organisms (dimensionless, on a scale from 0 to 1). The landscape was “continent” for *CFs* derivation to accommodate geographic diversity, and “emission to freshwater” was designated as the end-of-life compartment. We attempted to identify sensitive factors and capture all possible *CF*s for IMI and nano-IMI by setting up various scenarios; *EF*, *FF*, and *XF* under various scenarios are summarized in Fig. 2.

***Effect factor derivative.*** The *EF* was calculated using equations (4), (5):

(4) $$EF=\frac{PAF}{{HC}_{50}}=\frac{0.5}{{HC}_{50}}$$

(5) $${HC}_{50}=\sqrt[{n}]{\prod_{i=1}^{n}{EC}_{50,i}}\to {\log\;HC}_{50}=\frac{1}{n}\sum_{i=1}^{n}{\log\;EC}_{50,i}$$

where *HC*_50_ (hazardous concentration) is calculated by the geometric mean of the median effective concentration (*EC*_50_, μg L^−1^) [47]. The *n* in equation (5) denotes the total number of tested species, and *i* indicates the number assigned to each species. While traditional models use PNEC's conservative approach, the model suggests using more robust parameters such as *HC*_50_s (derived from *EC*_50_s) for the impact assessment of substances. A PAF of 0.5 was selected based on the least data demand and uncertainty [47]. The USEtox recommends incorporating three chronic or sub-chronic species-level toxicity data points from three trophic levels (e.g., crustaceans, fish, and algae) for the *EF* derivative. However, due to the limited toxicity data available for nano-IMI, the *CFs* estimated in this study are “indicative,” with a significant level of uncertainty. Here, we conducted acute and chronic toxicity tests on embryonic zebrafish (*Danio rerio*), crustaceans (*Daphnia magna*), and a benthic organism (*Chironomus kiinensis*) in our laboratory to obtain basic toxicity data for nano-IMI (Supplementary Material Table S26).

By comparison, toxicological data for IMI are abundant. To minimize data selection bias, four scenarios considering toxicity data usage are summarized in Table S27–S29 (Supplementary Material). In all cases, *EF* was the default value derived from the USEtox model. For *EF*_1_, our research employed the same species selection for both IMI and nano-IMI to facilitate comparative analysis. *EF*_2_ omitted insect data and included only non-target species, and *EF*_3_ considered all species available. All toxicity data are based on freshwater organisms (Supplementary Material Text S3.1).

***Fate factor derivative***. The freshwater fate matrix $\bar{FF}$ for IMI and nano-IMI were derived by calculating the negative inverse of the transfer rate coefficient matrices $\bar{K}$ and $\bar{I}$ (equations (6), (7)) [48]:

(6) $${\bar{FF}}_{\text{IMI}}=-{\bar{K}}^{-1}$$

(7) $${\bar{FF}}_{\text{nano}-\text{IMI}}=-{\bar{I}}^{-1}$$

Elements within these matrices represent the respective *FF* of IMI and nano-IMI in individual environmental compartments (Supplementary Material Text S3.2). For nano-IMI, the fate factors can be calculated using equations (8), (9), reflecting two scenarios (S1 and S2) of hetero-aggregation:

(8) $${FF}_{\text{nano}-\text{IMI},S1}={FF}_{\text{free}}$$

(9) $${FF}_{\text{nano}-\text{IMI},S2}={FF}_{\text{free}}+{FF}_{\text{agg}}+{FF}_{\text{att}}$$

where *FF*_free_, *FF*_agg_, and *FF*_att_ correspond to the *FF* of nano-IMI in its free, aggregated, and attached forms within the freshwater compartment. More details are provided in Text S3.2 (Supplementary Material).

***Exposure factor derivative.*** The *XF* represents the bioavailability of the chemical to humans or other species, which can be quantified as the fraction of the chemical transferred to the receptor population/species within a specific time period [20]. In previous studies, *XF* was generally assumed as 1, indicating that all chemicals were bioavailable to organisms [27,43,49]. Here, taking into account the PECs of IMI in the freshwater compartment, an additional *XF* was considered using equation (10) for IMI:

(10) $${XF}_{\text{IMI}}=\frac{1}{1+k_{\text{sus},\text{IMI}}\times \frac{C_{\text{sus}}}{1000}+k_{\text{doc},\text{IMI}}\times \frac{C_{\text{doc}}}{1000}+{BAF}_{\text{fish},\text{IMI}}\times \frac{C_{\text{biota}}}{1000}}$$

where *k*_sus,IMI_ and *k*_doc,IMI_ represent the suspended solids/water partitioning coefficient and dissolved (colloidal) organic carbon/water partition coefficient of IMI, respectively. *BAF*_fish,IMI_ denotes the bioaccumulation factor of IMI in fish, while *C*_sus_ and *C*_doc_ refer to the concentrations of suspended matter and dissolved (colloidal) organic carbon in freshwater, respectively. All relevant parameters are documented in Table S32 (Supplementary Material).

Given that the USEtox model was primarily developed for inorganic and organic substances, the current *XF* may not fully accommodate the peculiarities of nanoparticles [48]. Thus, the aggregated and attached nano-IMI in S1 were considered non-bioavailable, whereas all three forms of nano-IMI in S2 were deemed bioavailable. The *XF* of S1 and S2 were determined using equations (11), (12), respectively:

(11) $${XF}_{S1}=\frac{1}{1+{BCF}_{\text{free}}\times \left[Biota\right]}$$

(12) $${XF}_{S2}=\frac{1}{1+{BCF}_{\text{free}+\text{agg}+\text{att}}\times \left[Biota\right]}$$

where *BCF* is the bioconcentration factor of nano-IMI in freshwater organisms (mL g^−1^), and [*Biota*] is the biology concentration in the freshwater environment (1 mg L^−1^). Previous studies have quantified the temporal uptake and depuration of both pesticides and determined their *BCF*s in freshwater organisms [9] (Supplementary Material Text S3.3 and Table S33).

### 2.4 Freshwater impact score

The freshwater *IS* quantifies the ecotoxicity of chemicals released into specific environmental compartments, measured in comparative toxic units (CTUe), which can also be described as PAF m^3^ d (equation (13)) [50]:

(13) $$IS={CF}_{i}\times m_{i}$$

where *m*_*i*_ (kg) represents the mass of chemical *i* emitted into freshwater. Given the rapid release of the IMI content from nano-IMI in freshwater [9], the modified equation (14) was used to determine the freshwater *IS* of nano-IMI:

(14) $${IS}_{\text{nano}-\text{IMI}}={CF}_{\text{nano}-\text{IMI}}\times m_{\text{nano}-\text{IMI}}+{CF}_{\text{IMI}}\times m_{\text{released}-\text{IMI}}$$

where *m*_released-IMI_ represents the total mass of IMI released from nano-IMI into freshwater. The total IMI and nano-IMI emissions were calculated based on the emission fractions obtained from the multimedia environmental model and the annual pesticide application rates.

### 2.5 Sensitivity and uncertainty analyses

A sensitivity analysis was performed to identify the parameters significantly influencing the LCA results to produce IMI and nano-IMI. Each input parameter was altered by 20 %, with all other parameters held constant, and the system's potential environmental impact was recalculated to evaluate the effects of these changes on the overall LCA results. A parameter was considered sensitive if its sensitivity factor was more than 2 %.

An uncertainty analysis was conducted using Monte Carlo simulations, executing 1000 iterations with a 95 % confidence interval to determine the highest and lowest bounds for the environmental impacts of IMI and nano-IMI production [28]. Furthermore, *IS* under various scenarios, including rainfall conditions and variations in α, aggregation scenarios, and differing toxicity data selection, were systematically examined (Fig. 2).

## 3 Results and discussion

### 3.1 Environmental impacts of IMI and nano-IMI production

**Fig. 3:**
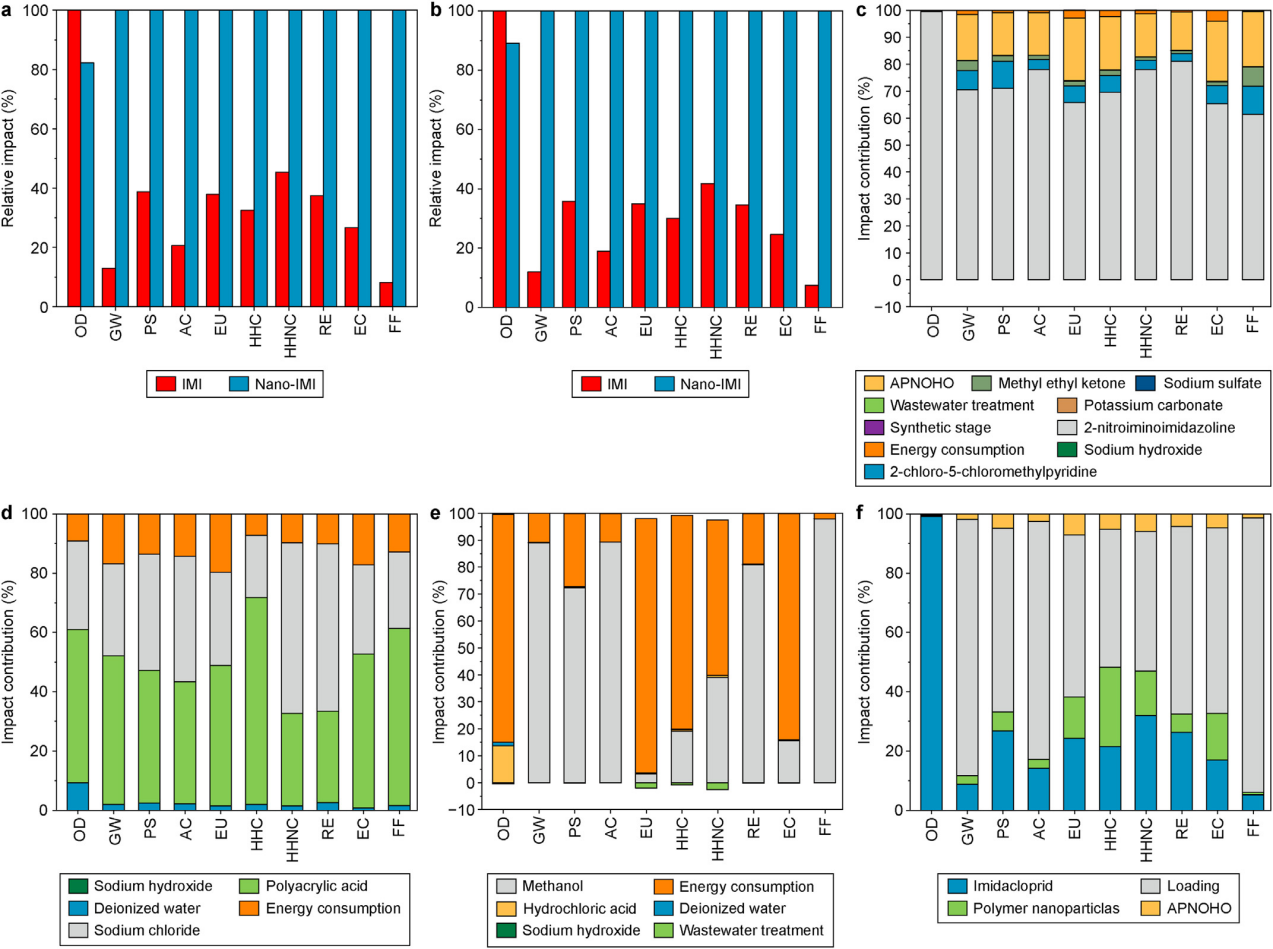
a–**b**, Impact comparison between imidacloprid (IMI) and nano-IMI based on the mass production (1 kg; **a**) and the amount of pesticide application per unit area (IMI: 2.12 kg ha^−1^; nano-IMI: 2.30 kg ha^−1^; **b**). **c**–**f**, Environmental impact contribution during the IMI production (**c**), nanocarrier production (**d**), loading process (**e**), and the overall production of nano-IMI (**f**, including panels **c**–**e**). The *x*-axis is environmental impact categories derived by the tool for the reduction and assessment of chemical and other environmental impacts (TRACI): ozone depletion (OD), global warming potential (GW), smog (PS), acidification (AC), eutrophication (EU), human health-carcinogenic (HHC), human health-noncarcinogenic (HHNC), respiratory effects (RE), ecotoxicity (EC), and fossil fuel depletion (FF).

The environmental impacts and input contributions during IMI and nano-IMI production are depicted in Fig. 3. The ecotoxicity of nano-IMI during production was 2.12 × 10^3^ CTUe, which is approximately four times higher than that of IMI (566 CTUe) (Supplementary Material Table S19 and Fig. 3a). This is primarily due to the encapsulation process used for nano-IMI, which involves additional solvents (e.g., methanol) and techniques (e.g., evaporation and freeze-drying) to encapsulate IMI and synthesize nano-IMI. Moreover, nano-IMI requires a higher total mass per unit area (2.30 kg ha^−1^) than IMI (2.12 kg ha^−1^) as recommended by the application guidelines, due to its lower active ingredient content. Nevertheless, applying similar quantities did not significantly impact the environmental consequences of the two pesticides (Fig. 3b).

Over 60 % of IMI's production-related environmental impacts originates from the synthesis of 2-nitroaminoimidazoline, an intermediate compound, followed by the adjuvant used (APNOHO) (Fig. 3c). The production of nano-IMI involves three stages: IMI synthesis, nano-carrier synthesis (Fig. 3d), and IMI encapsulation (Fig. 3e). The loading process had the highest impact among nine impact categories during nano-IMI production, except for ozone depletion (Fig. 3f). Specifically, the nano-encapsulation procedure accounted for approximately 62.6 % of the ecotoxicity, followed by IMI production (17 %) and nano-carrier synthesis (15.7 %).

Methanol and energy consumption were environmental hotspots during the loading process. Ozone depletion was comparable during both IMI and nano-IMI production, which was mainly attributed to the high quantity of chloroform solvents used to produce 2-nitroaminoimidazoline. The sensitivity analysis showed that 2-nitroaminoimidazoline, APNOHO, pyridine, methyl ethyl ketone, and energy consumption were the most sensitive parameters for IMI production (Supplementary Material Table S20), whereas IMI production, nanocarrier production, APNOHO, methanol, and energy consumption were the sensitive parameters for nano-IMI production (Supplementary Material Table S21 and Fig. S17).

Overall, the production of nano-IMI imposed higher environmental burdens than that of IMI. Owing to the lack of comprehensive LCAs for nanopesticides in the literature, we compared the environmental impacts of nano-IMI with those of other metal-based nanoparticles (Supplementary Material Fig. S18). The impacts associated with nano-IMI production are low compared to those of the synthesis of various ENPs. For instance, studies have demonstrated that the ecotoxicity of manufacturing 1 kg of silver nanoparticles (Ag NPs) ranged from 2.08 × 10^3^ to 1.37 × 10^5^ CTUe, which is one to two orders of magnitude higher than that for nano-IMI production (2.12 × 10^3^ CTUe) [51,52]. From a production perspective, nano-IMI may surpass other metal-based nanopesticides due to its reduced environmental impact. Furthermore, applying green synthesis, grounded in the principles of green chemistry, for producing nanomaterials could significantly reduce their ecological impact. For instance, iron-oxide nanoparticles synthesized via green methods exhibit ecotoxicity three orders of magnitude lower than those produced using conventional co-precipitation methods [53]. Thus, optimizing the nano-IMI production process in accordance with green chemistry principles—for instance, using non-toxic compounds derived from biological resources (such as natural plant extracts) as nanocarrier materials [54], substituting solvents with low-toxic alternatives, or utilizing clean energy sources—holds significant potential for reducing ecotoxicity.

### 3.2 Predicted environmental concentrations in freshwater

The environmental transport coefficients for IMI and nano-IMI under three distinct rainfall events are detailed in Sheet 5 (Supplementary Data 1 and 2). The proportions of IMI discharged into freshwater ecosystems depended significantly on the rainfall intensity, with distribution percentages of 7.9 %, 76.2 %, and 93.1 % under limited (R_1_), normal (R_2_), and heavy (R_3_) rainfall scenarios, respectively (Supplementary Material Table S23). The freshwater discharge fraction of IMI exhibited a broader range than the fractions reported in earlier research (21 % and 68 %), mainly due to the more extreme rainfall scenarios considered in the present study [55]. By contrast, nano-IMI showed a significantly lower multicompartment distribution compared to IMI, ranging from 0.0003 % to 1.32 % across the three rainfall scenarios (Supplementary Material Table S24). Our model suggests that once nano-IMI was introduced into agricultural soil, it mainly aggregated with natural colloids (45.05–45.77 %) in soil pore water or attached to solid grains (54.21–54.93 %), with only a small percentage (less than 0.1 %) entering freshwater through surface runoff and soil erosion (Supplementary Data 2 Sheet 1).

**Fig. 4:**
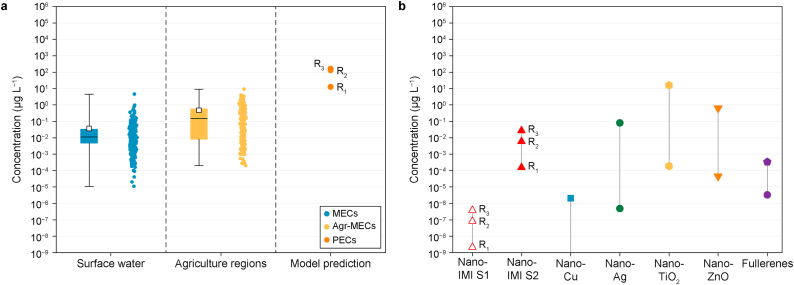
a, The measured environmental concentrations (MECs) and predicted environmental concentrations (PECs) of imidacloprid (IMI) considering three rainfall scenarios. Whiskers represent the summarized upper and lower MECs of IMI in surface water and agricultural regions (Agr-MECs) [70]. **b**, The PECs ranges of nano-IMI and representative engineered nanoparticles (ENPs) collected from the literature [29].

To validate the precision of the fate model, historical environmental IMI concentrations measured in surface water (MECs) and agricultural regions (agr-MECs) were compiled for comparison (Fig. 4a). The PECs of IMI in surface water were one to two orders of magnitude higher than the corresponding MECs, primarily due to two factors. First, the model assumed that the spatial scale (continental) comprised all agricultural fields, which largely amplified the scale of land receiving pesticide application and, therefore, the total amount of pesticides utilized. In the present study, if we considered that 42 % of the land comprised agricultural fields [56], the PECs for IMI would range from 1.85 × 10^−2^ to 22.9 μg L^−1^, similar to the previously reported agr-MECs (2.00 × 10^−4^ to 9.14 μg L^−1^) (Supplementary Material Table S25). Second, owing to the lack of actual data for nano-IMI entering the soil after application, 100 % of the nano-IMI and IMI applied to the field was assumed to enter the soil compartment, initiating their distribution in the environment. During agricultural spraying, approximately 30–50 % of pesticides are dispersed into the air through drift and evaporation [57], with 9.5 % drifting onto non-agricultural land [58]. The percentage of pesticides entering agricultural soil was approximately 5–37 % due to the combined effects of crop absorption and leaf surface interception [59]. While accounting for these variations, the PECs were found to be similar to the MECs in previously reported studies (Supplementary Material Fig. S19). Although introducing correction factors could reduce the variation between PECs and MECs for IMI, the lack of corresponding data for nano-IMI necessitated assuming 100 % discharge to simulate a worst-case scenario, facilitating an equivalent comparison between the two pesticides. Furthermore, a comparison of the worst-case PECs range for nano-IMI in surface water with those of other ENPs revealed that nano-IMI exhibited similar ranges to CuO and Ag nanoparticles, as reported in freshwater studies (Fig. 4b).

### 3.3 Freshwater *CF*s of IMI and nano-IMI

***EF.*** The *EF* of nano-IMI was 373 PAF m^3^ per kg emission using toxicity data from our previous laboratory experiments (summarized in Supplementary Material Table S26) [9]. Due to the toxicity data's limitation to three species across only two trophic levels, the USEtox model categorized the *EF* of nano-IMI as “indicative.” The selection of toxicity data could greatly influence the reliability of *EF* estimates. Accordingly, four *EFs* were calculated for IMI, using different sets of toxicological data to enable meaningful comparisons while minimizing uncertainties (Supplementary Material Table S27–S29). The default *EF* for IMI in USEtox, based on eight species across two trophic levels, was 59.4 PAF m^3^ per kg emission. When restricted to the same species as nano-IMI, the *EF*_1_ of IMI increased to 2.64 × 10^3^ PAF m^3^ per kg emission, approximately sevenfold the *EF* of nano-IMI. Moreover, the recalculated *EF*_2_ and *EF*_3_ for IMI, considering species across three trophic levels with more toxicity data collected, were orders of magnitude higher than the *EF* of nano-IMI (Supplementary Material Table S30). Excluding insects, *EF*_2_ was notably higher, at 9.01 × 10^3^ PAF m^3^ per kg emission, while *EF*_3_, which included all species, reached 7.46 × 10^4^ PAF m^3^ per kg emission. Although the *EF* included some degrees of uncertainty owing to the scarcity of toxicity data, our analysis included extensive toxicity data for IMI to mitigate potential uncertainties arising from the selection of such data. These scenarios could further propagate to the corresponding *CFs* to capture more possible scenarios. However, acquiring more ecotoxicity data for nano-IMI is still important to enhance the accuracy of future risk assessments.

***FF.*** The calculated *FF* for IMI was 183.7 d, exceeding the default value of approximately 54 d, as suggested by the USEtox model. This discrepancy primarily results from using different model parameters and excluding additional environmental compartments, such as seawater and natural soil. When hetero-aggregation was considered as a removal process to eliminate nano-IMI from the freshwater system, three different attachment efficiencies (α_1_, α_2_, and α_3_) resulted in *FF* values of 0.057, 0.020, and 0.021 d (Supplementary Material Table S31), respectively. These results indicate that nano-IMI could be removed from freshwater within 2 h and, once removed, it is no longer bioavailable to freshwater organisms. Notably, the hetero-aggregation rate was significantly higher than the other rate constants (Supplementary Data 2 Sheet 9), establishing it as the predominant process affecting *FF*.

In S2, the aggregated nano-IMI remained in the water column and settled down gradually to the sediment; therefore, it may still have been bioavailable during the aggregation process, extending the *FF* to 1.8 d for α_1_ and 1.76 d for α_2_ and α_3_ (Supplementary Material Table S31). Under this scenario, the release of the active ingredient from nano-IMI was considered a dissolution process. Due to its extremely rapid dissolution [9], the release of the active ingredient from nano-IMI was a key part of the clearance process in freshwater (*k*_*diss*_ = 1.00 × 10^−5^ s^−1^), markedly higher than the other rate constants. Consequently, the release of active ingredients was identified as the dominant process for removing nano-IMI from freshwater under this scenario. Additionally, the ecotoxicity of the released active ingredient (as IMI) was further considered in the final *IS* derivative.

***XF***. The *XF* of IMI closely resembled the default *XF* of IMI in the USEtox model, with all *XF*s being close to 1 (0.998 and 0.99997) (Supplementary Material Table S32). The *BCF* of nano-IMI under two aggregation scenarios showed only slight differences, resulting in similar *XF*_S1_ and *XF*_S2_ (0.9999997 and 0.9999995) (Supplementary Material Table S33). However, owing to their rapid dissolution (release of the active ingredient), the calculated *XF* for nano-IMI in this study approached 1, suggesting similar bioavailability to IMI. Although minimal disparities were found for *XFs*, the comprehensive consideration of nano-IMI's bioavailability effectively reduces uncertainties in the results. Previous studies have commonly assumed an *XF* of 1 for ENMs, overlooking bioavailability variations among ENMs [43]. Previous studies have calculated *XF* values for graphene oxide [60], nano silver [61], and nano copper [62] at 0.93, 0.80, and 0.33, respectively, indicating potentially higher bioavailability for nano-IMI in freshwater organisms.

**Fig. 5:**
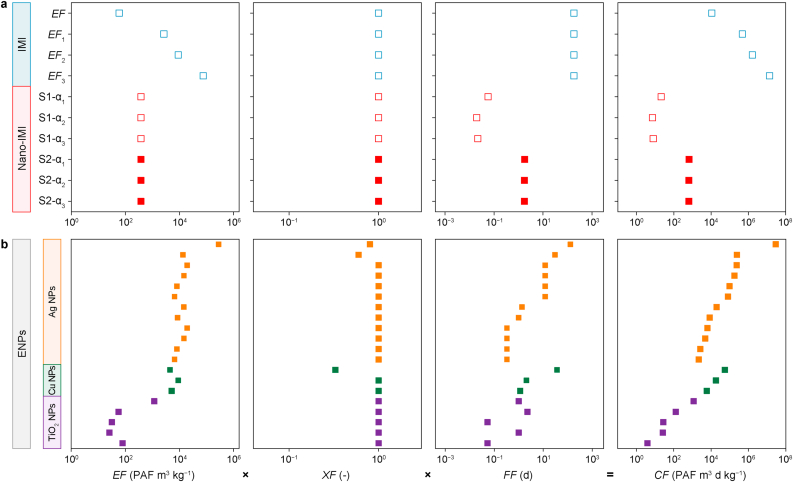
a, The effect factor (*EF*), exposure factor (*XF*), fate factor (*FF*), and characterization factor (*CF*) of IMI and nano-IMI under various scenarios. **b**, The same factors for representative engineered nanoparticles (ENPs) retrieved from the literature are used for comparison, including silver, copper, and titanium dioxide nanoparticles [27,29,43]. Different *EF*s denote distinct organism selections: *EF* is the default species set in USEtox, *EF*_1_ matches the species selected in nano-IMI, *EF*_2_ includes species across three trophic levels (excluding insects), and *EF*_3_ incorporates all species across three trophic levels. S1 and S2 represent two scenarios where hetero-aggregation is regarded as a clearance or transformation process, respectively. α_1_, α_2_, α_3_ are attachment coefficients which are summarized in Supplementary Data 2 Sheet 2.

***CF***. The *CFs* of IMI ranged from 3.20 × 10^3^ to 1.3 × 10^7^ PAF m^3^ d per kg emission (Supplementary Material Table S34), with this variation largely attributable to differences in its *EF* (Fig. 5a). These *CFs* fell within the median to upper range of those reported for pesticides in previous studies (Supplementary Material Table S36). Owing to the different *FFs* calculated based on two aggregation scenarios, the *CF*s of nano-IMI ranged from 7.3 to 21.3 PAF m^3^ d per kg emission under S1 and from 656 to 670 PAF m^3^ d per kg emission under S2 (Supplementary Material Table S35). Hetero-aggregation, which prolongs the residence time of nano-IMI in freshwater, led to increased *CFs* under S2.

By comparison, the *CF*s of nano-IMI were lower than those of metal-based ENPs in freshwater (Fig. 5b). For instance, the *CFs* for Ag NPs ranged from 2.19 × 10^3^ to 6.42 × 10^3^ PAF m^3^ d per kg emission in S1 and from 7.98 × 10^4^ to 2.34 × 10^5^ PAF m^3^ d per kg emission in S2, substantially exceeding those of nano-IMI in analogous scenarios [43]. Additionally, other studies have reported *CFs* for Cu and TiO_2_ NPs of 3.88 × 10^3^ to 1.11 × 10^4^ and 1.55 × 10^3^ PAF m^3^ d per kg emission, respectively, both surpassing those of nano-IMI [27,29]. Collectively, these findings suggest that nano-IMI may exhibit lower end-of-life ecotoxicity compared to IMI and typical commercialized metal-based ENPs.

### 3.4 Freshwater impact scores and integrated risks

**Fig. 6:**
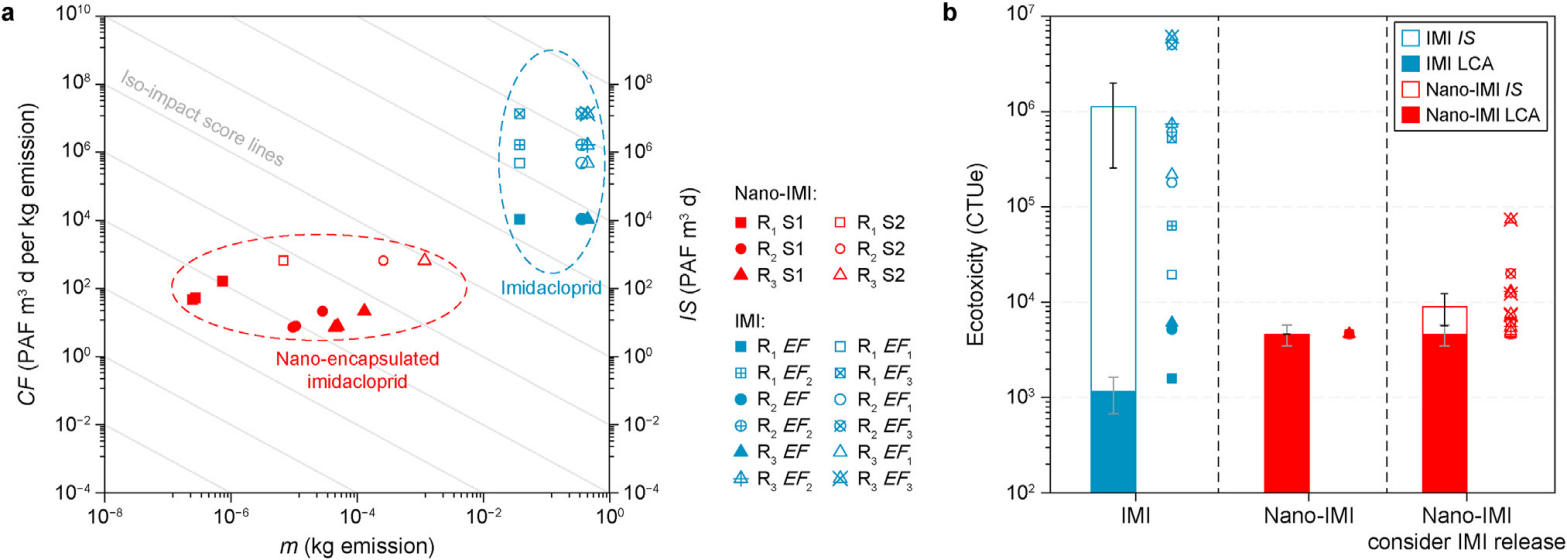
Impact scores (*IS*, **a**) and life cycle ecotoxicity (**b**) of imidacloprid (IMI) and nano-IMI under different scenarios. In panel **a**, *IS* (right-side *y*-axis) are plotted as a product of freshwater emission mass (*x*-axis) and midpoint characterization factor (*CF*s; left-side *y*-axis). *m* represents the mass (g) of pesticides emitted to freshwater compartments under the scenario of the annual per-hectare quantity of pesticide applied. In panel **b**, the black whiskers represent the 95 % confidence intervals of the *IS* for IMI and nano-IMI, while the gray whiskers represent the 95 % confidence intervals of life cycle assessment (LCA) results for IMI and nano-IMI. Data points in panel **b** are summarized life cycle ecotoxicity data of IMI and nano-IMI under all considered scenarios. R represents distinct rainfall scenarios: limited rainfall (R_1_), normal rainfall (R_2_), and heavy rainfall (R_3_). Different *EF*s denote distinct organism selections: *EF* is the default species set in USEtox, *EF*_1_ matches the species selected in nano-IMI, *EF*_2_ includes species across three trophic levels (excluding insects), and *EF*_3_ incorporates all species across three trophic levels. S1 and S2 represent two scenarios where hetero-aggregation is regarded as a clearance or transformation process, respectively.

Here, freshwater *IS* represents the multiplication of pesticides emitted per emission scenario by the corresponding toxicity *CF*, considering all scenarios under which pesticides are emitted. The *IS* of IMI under the limited, normal, and heavy rainfall scenarios ranged from 411 to 5.17 × 10^5^, 3.99 × 10^3^ to 5.01 × 10^6^, and 4.88 × 10^3^ to 6.12 × 10^6^ CTUe, respectively (Supplementary Material Table S37). A previous study quantified the *IS* of 12 pesticides, which ranged from 3.77 × 10^−12^ to 1.83 × 10^6^ CTUe, covering most of the range of *IS* for IMI [63]. The *IS* of IMI calculated in this study fell within the median to upper range for the pesticide cohort, mainly due to the excessive application and worst-case scenario assumed in this study. Comparatively, the freshwater *IS* of nano-IMI was consistently magnitudes lower than that of IMI under all corresponding scenarios, ranging from 1.56 × 10^−4^ to 7.74 CTUe (Fig. 6a, Supplementary Material Table S38).

As the released IMI from nano-IMI could potentially have a similar impact on the ecosystem, additive impacts were considered for the mixture (nano-IMI and released IMI) and were further included in the *IS* of nano-IMI. Considering the release of IMI into freshwater from nano-IMI, the *IS* ranges for nano-IMI (Supplementary Material Table S39) were enhanced to 0.012–400 (R_1_), 0.453–1.53 × 10^4^ (R_2_), and 2.1–6.93 × 10^4^ CTUe (R_3_). The *IS* values resulting from the release of nano-IMI were still substantially lower than those of IMI. The considerable disparity between the two formulations can be ascribed to their notable differences in freshwater emissions and their pronounced variability in ecotoxicity (*CF*).

The inclusion of *IS* as an additional parameter in LCA can reflect the potential “cradle-to-grave” risks associated with a substance. After merging the *IS* and LCA results, nano-IMI generally exhibited a lower freshwater ecotoxicity risk than IMI across most scenarios. IMI's ecotoxicity spanned 1.61 × 10^3^ to 6.13 × 10^6^ CTUe, whereas nano-IMI was restricted within a range of 4.89 × 10^3^ to 7.42 × 10^4^ CTUe. The overall freshwater risk of nano-IMI only surpassed that of IMI under limited rainfall scenarios (R_1_) (Fig. 6b). Furthermore, given the challenges associated with the release kinetics of nanopesticides, we quantified the impacts stemming from their released active ingredients. Nano-IMI still displayed lower freshwater risks compared to IMI under each corresponding scenario, suggesting its potential as a substitute that could reduce freshwater risks associated with pesticide applications. From a life cycle perspective, the production of nano-IMI is less advantageous than that of IMI, due to the additional synthesis processes required, while concerning end-of-life pesticide release, IMI was more prone to freshwater entry, potentially yielding a higher risk than nano-IMI in freshwater.

It is essential to prioritize safety and minimize potential risks associated with the different life cycle stages of these novel nano-agrochemicals. Strategies include developing eco-friendly and more effective variants, refining production processes based on identified LCA hotspots, and adhering to green chemistry principles [64]. Previous studies have proposed a life cycle-based alternative assessment framework for chemical substitution that, along with identifying toxic substances, suggests evaluating the chemical supply chain to enhance the sustainable design of these new entities [[31], [71]]. Additionally, robust regulatory measures and the establishment of strict usage guidelines are necessary to reduce potential risks. Providing comprehensive training and education to farmers on using nanopesticides properly is crucial, especially to emphasize their synergistic mechanisms that reduce application rates and enhance efficiency.

### 3.5 Limitations and uncertainties

Our results suggest that the pronounced tendency of IMI to enter freshwater could pose significant adverse effects on non-target aquatic organisms. However, the residues of nano-IMI were notably higher in soil compared to other compartments, persisting even after partitioning and distribution (Supplementary Material Table S24). Therefore, it is crucial to research introducing nano-IMI to agricultural soil to investigate its potential ecotoxicity, bioavailability to soil organisms, foliar traits, and fate parameters to elucidate potential risks to the soil environment. In addition, this study examines the life cycle environmental impacts of nano-IMI production at a laboratory level due to the absence of industrial-scale data. Comparing both pesticides on the same laboratory basis potentially minimizes underlying uncertainties [65]. Since scaling production from the laboratory to an industrial level can effectively reduce the corresponding environmental impacts [66], future work exploring industry-level life cycle inventories would significantly enhance the accuracy of these impact assessments.

Despite inherent uncertainties, this study has comprehensively evaluated multiple scenarios related to the fate and effects of nanopesticides, thereby reducing potential uncertainties and providing a practical approach for regulating and promoting the sustainable development of nanopesticides from a life cycle perspective. Future efforts could focus on enhancing model precision and reducing uncertainties in ecological risk assessments (ERA) of nano-agrochemicals. Specifically, the lack of precise application and emission data for nanopesticides hindered verifying their environmental concentrations; emerging approaches such as field monitoring and material flow analysis could address this deficiency [67]. Additionally, improving the accuracy of *CF*s for nanopesticides requires comprehensive toxicity data that reflect their diverse properties (e.g., size and carrier agents) and the development of more sensitive model inputs (e.g., attachment coefficients) [68,69].

## 4 Conclusions

The globally recognized need for more sustainable agriculture and food systems has motivated the development and application of nanopesticides. A rigorous assessment conducted before their broad implementation would act as a fundamental safeguard for environmental safety. By harmonizing ERAs' localized impact focus with LCAs’ systems perspective, this framework advances nano-agrochemical risk assessment beyond single phase-limited paradigms, providing a robust methodological framework for the safe application and risk governance of novel chemicals. Although LCA can elucidate the environmental impacts of nano-agrochemicals production, it overlooks the nano-specific properties during product life stages and the end-of-life ecological impacts. To overcome the inconsistency, the present study integrates the results obtained from systematical models and comprehensively addresses the relative *CF*s and associated ecological risks associated with nanopesticide production, application, and release throughout their lifecycle. Under identical rainfall conditions, nano-IMI exhibited a substantially reduced integrated freshwater risk compared to IMI, showing potential as an alternative to conventional IMI.

Our study, for the first time, provides a systematic methodology for verifying the data necessary for risk management of nano-agrochemicals. By incorporating the unique fate, exposure, and effects of nanopesticides, uncertainties related to environmental parameters and nano-specific behaviors and effects were minimized, significantly enhancing the robustness of the results. While critical components such as life cycle inventory databases, multimedia distribution processes, and endpoint toxicity characterization are inherently influenced by regional and environmental variables, the model framework developed in this research study parameterizes key environmental factors, enabling regionally tailored applications without requiring extensive structural modifications. The parameterization of environmental drivers allows for seamless adaptation to diverse agroecological contexts, addressing a critical gap in global nano-agriculture regulation.

## CRediT authorship contribution statement

**Mingyan Ke:** Writing – original draft, Formal analysis. **Keshuo Zhang:** Data curation. **Andrea L. Hicks:** Writing – review & editing. **Fan Wu:** Writing – review & editing, Supervision, Project administration, Investigation, Conceptualization. **Jing You:** Writing – review & editing, Supervision.

## Declaration of competing interest

The authors declare that they have no known competing financial interests or personal relationships that could have appeared to influence the work reported in this paper.
