# Supplementary material for: A life cycle risk assessment of nanopesticides in freshwater
Source: Environ Sci Ecotechnol. 2025 May 2;25:100565. doi: 10.1016/j.ese.2025.100565 (PMC12136839; doi:10.1016/j.ese.2025.100565)
Supplement: Multimedia component 1 [file mmc1.docx]

**Supplementary Material**

**A Life Cycle Risk Assessment of Nanopesticides in Freshwater**

Mingyan Ke,^1^ Keshuo Zhang,^1^ Andrea L. Hicks,^2^ Fan Wu,^1,*^ Jing You^1^

*^1^ College of Environment and Climate, Guangdong Provincial Key Laboratory of Environmental Pollution and Health, Jinan University, Guangzhou, 511443, China*

*^2^ Department of Civil and Environmental Engineering, University of Wisconsin-Madison, Madison, WI 53706, USA*

*Corresponding author.

Email: fanwu@jnu.edu.cn

39 **Tables**, 19 **Figures**

53 Pages

**Text S1**. Life cycle assessment of IMI and nano-IMI production.

**Text S2**. Simplebox and simplebox4Nano model matrices predict the multimedia distribution of IMI and nano-IMI.

**Text S3**. Freshwater characterization factors (*CF*s) of IMI and nano-IMI.

3.1 *Effect factor* (*EF*)

3.2 *Fate factor* (*FF*)

3.3 *Exposure factor* (*XF*)

**List of Tables**

Table S1. Inputs/outputs to produce isobutylene.

Table S2. Inputs/outputs to produce tert-butanol.

Table S3. Inputs/outputs to produce sodium tert-butoxide.

Table S4. Inputs/outputs to produce 2,3-dichloropropionaldehyde (CAS: 10140-89-3).

Table S5. Inputs/outputs to produce 4-chloro-4-(chloromethyl)-5-oxopentanenitrile (CCC, CAS: 150807-86-6).

Table S6. Inputs/outputs to produce 2-chloro-5-chloromethylpyridine (CAS: 70258-18-3).

Table S7. Inputs/outputs to produce 2-nitroaminoimidazoline (CAS: 5465-96-3).

Table S8. Inputs/outputs to produce the active ingredient of imidacloprid (IMI).

Table S9. Inputs/outputs to produce potassium bisulfate.

Table S10. Inputs/outputs to produce potassium persulfate.

Table S11. Inputs/outputs to produce polyacrylic acid (PAA).

Table S12. Inputs/outputs to produce polymer nanoparticles.

Table S13. Inputs/outputs to produce the active ingredient (contain the nanocarrier) of nano-encapsulated imidacloprid (nano-IMI).

Table S14. Inputs/outputs to produce nonylphenol.

Table S15. Inputs/outputs to produce polyethylene glycol nonylphenyl ether (APNOHO, CAS: 9016-45-9).

Table S16. Inputs/outputs to produce IMI.

Table S17. Inputs/outputs to produce nano-IMI.

Table S18. Calculations and assumptions for energy consumption during material syntheses.

Table S19. TRACI characterization results for IMI and nano-IMI production under different functional units.

Table S20. Sensitivity analysis for IMI production.

Table S21. Sensitivity analysis for nano-IMI production.

Table S22. The emission rate of IMI and nano-IMI in multicompartment (vector *e*).

Table S23. Multimedia distribution of IMI under three rainfall scenarios (R_1_: limited rainfall, R_2_: normal rainfall, R_3_: heavy rainfall).

Table S24. Multimedia distribution of nano-IMI under three rainfall scenarios (R_1_, R_2_, R_3_) and three attachment coefficients (α_1_, α_2_, α_3_).

Table S25. Predicted environmental concentrations (PEC) of IMI and nano-IMI in the freshwater compartment.

Table S26. Ecotoxicity data of nano-IMI for effect factor (*EF*) derivative.

Table S27. Ecotoxicity data of IMI for effect factor (*EF*_1_) derivative.

Table S28. Additional toxicity data considered to derive the effect factor (*EF*_2_) of IMI.

Table S29. Additional toxicity data considered to derive the effect factor (*EF*_3_) of IMI.

Table S30. Summarized effects factors (*EF*) for IMI and nano-IMI.

Table S31. The freshwater fate factor (*FF*) of IMI and nano-IMI.

Table S32. Inputs and output for IMI’s exposure factors (*XF*) derivative.

Table S33. Inputs and output for nano-IMI’s exposure factors (*XF*) derivative based on different perspective on hetero-aggregation of nano-IMI in fresh water.

Table S34. The characterization factors (*CF*s) of IMI considering four different *EFs*.

Table S35. The characterization factors (*CF*s) of nano-IMI based on two hetero-aggregation scenarios and three attachment coefficients.

Table S36. Summarized freshwater characterization factors (*CF*s) of conventional pesticide from literature.

Table S37. Integrated impact score (*IS*) and life cycle ecotoxicity of IMI.

Table S38. Integrated impact score (*IS*) and life cycle ecotoxicity of nano-IMI (ignoring IMI release from nano-IMI).

Table S39. Integrated impact score (*IS*) and life cycle ecotoxicity of nano-IMI (considering IMI release from nano-IMI).

**List of Figures**

Figure S1. Main process flow of IMI and nano-IMI production.

Figure S2. Scheme for isobutylene production.

Figure S3. Scheme for tert-butanol production.

Figure S4. Scheme for sodium tert-butoxide production.

Figure S5. Scheme for 2,3-dichloropropionaldehyde (CAS: 10140-89-3) production.

Figure S6. Scheme for 4-chloro-4-(chloromethyl)-5-oxopentanenitrile (CCC, CAS: 150807-86-6) production.

Figure S7. Scheme for 2-chloro-5-chloromethylpyridine (CAS: 70258-18-3) production.

Figure S8. Scheme for 2-nitroaminoimidazoline (CAS: 5465-96-3) production.

Figure S9. Scheme for the active ingredient production of imidacloprid (IMI).

Figure S10. Scheme for potassium bisulfate production.

Figure S11. Scheme for potassium persulfate production.

Figure S12. Scheme for polyacrylic acid (PAA) production.

Figure S13. Scheme for polymer nanoparticles production.

Figure S14. Scheme for the active ingredient (contain the nanocarrier) production of nano-imidacloprid (nano-IMI).

Figure S15. Scheme for nonylphenol (CAS: 25154-52-3) production.

Figure S16. Scheme for polyethylene glycol nonylphenyl ether (APNOHO, CAS: 9016-45-9) production.

Figure S17. Sensitivity analyses of IMI and nano-IMI production.

Figure S18. Ecotoxicity of nano-IMI and ENPs during life cycle production.

Figure S19. Prediction environmental concentration (PEC) of IMI considering multiple factors (Pesticide drift, plant absorption, proportion of agricultural soil at continental scale, etc.).

**Text S1: Life cycle assessment of IMI and nano-IMI production**

1.1 Imidacloprid synthesis

Following a comprehensive review on SciFinder, we selected the optimal synthesis approach for imidacloprid (IMI) that demonstrated the highest yield. Using patent CN103641815A, we established the Life Cycle Inventory (LCI) for IMI, which improved the production process of imidacloprid, achieving a remarkable yield of up to 98.7%. The synthesis of the crucial intermediate, 2-chloro-5-chloromethylpyridine, was described in patent CN110483382A, yielding an impressive 91.2% [1]. This intermediate was synthesized by dissolving 2-chloro-2-chloromethyl-1-aldehyde valeronitrile (CCC, CAS: 150807-86-6) and the catalyst N, N-dimethylformamide (DMF) in toluene, followed by cyclization with phosgene. Detailed production protocols are available in Figure S7 and Table S6.

Additionally, the LCI for another intermediate, 2-nitroaminoimidazoline, was developed based on patent US5453529A, achieving a yield of 96.1%. This intermediate was produced by dissolving nitroimidodithiocarbonate and ethylenediamine in chloroform and then cyclized. These production details are illustrated in Figure S8 and Table S7. The final synthesis of imidacloprid involved a direct condensation of 2-chloro-5-chloromethylpyridine and 2-nitroaminoimidazoline under mild alkaline conditions at 40–50℃ [2], using methyl ethyl ketone as the solvent and potassium carbonate as the deacidification agent [3]. Detailed documentation of the IMI production process is provided in Figure S9 and Table S8.

1.2 Nano-imidacloprid synthesis

The production process for nano-imidacloprid (nano-IMI) was developed following the specifications of patent US20200260724A1, issued to Vive Crop Protection Co., LTD., Canada. This process comprises two primary stages: the synthesis of nanocarriers and the encapsulation of pesticide active ingredients [4]. The nanocarriers predominantly consist of polyacrylic acid (PAA), synthesized as per the methods outlined in patent WO2014056846A1. The process involves heating aqueous solutions of acrylic acid and potassium persulfate initiator at 80°C for 16 hours in a sealed vessel, followed by an open vessel treatment at 120°C for 1 hour to remove water and yield PAA [5]. The initiator, potassium persulfate, is produced by electrolyzing a solution of potassium bisulfate in sulfuric acid [6], where potassium bisulfate is a by-product of manufacturing nitric acid from potassium nitrate and sulfuric acid.

KNO_3_ + H_2_SO_4_ → KHSO_4_ + HNO_3_

The electrolytic reaction is:

2 KHSO_4_ → K_2_S_2_O_8_ + H_2_

To prepare the nanocarrier, solid PAA is dissolved in deionized water under UV irradiation at pH 9.63. This is followed by the addition of NaCl to decrease the polymer charge density, resulting in the formation of compact nanosphere carriers. These steps and associated details are available in Figures S10–S13 and Tables S9–S12.

During the loading phase, nanocarriers dispersed in methanol are mixed with IMI using a vortex mixer, allowing IMI to diffuse into the swollen nanospheres. Methanol is subsequently evaporated, anchoring IMI within the carriers. To improve nano-IMI suspension stability in water, the dry mixture from evaporation is dissolved in deionized water at pH 8 using NaOH, converted to a clear solution, and then freeze-dried into a polymer particle-laden powder [4]. Study suggests that dialysis bags can effectively isolate free IMI, achieving a 100% reuse rate [7]. The drug loading efficiency for nano-IMI, based on the literature, is estimated at 91% [8]. Further specifics are delineated in Figure S14 and Table S13.

1.3 Adjuvant APNOHO synthesis

Both IMI and nano-IMI pesticides comprise not only active pesticide ingredients but also inert ingredients that improve the products' physical properties. Nonetheless, the constituents of inert substances were not specified for IMI and nano-IMI. Given that APNOHO is an inert component in over 650 pesticide products, it was used as inert ingredient in IMI and nano-IMI [9, 10]. APNOHO was synthesized from nonylphenol and propylene oxide (refer to Figures S15–S16 and Tables S14–S15 for details) [11]. The composition ratios in IMI, as per the guidelines, indicate that it consists of 22.6% IMI and 77.4% inert compounds [12]. nano-IMI is composed of 18.5% IMI, 20.33% nanocarrier, and 61.17% APNOHO, determined from the encapsulating rates and instructions provided accordingly [13]. Additional information is detailed in Tables S16–S17.

**Text S2:** **Simplebox and simplebox4Nano model matrices predict the multimedia distribution of IMI and nano-IMI.**

The specific calculation process of matrix $\bar{K}$ and $\bar{I}$ are described in Supplementary material_2 and _3, respectively. The $\bar{K}$ [4×4] and $\bar{I}$ [15×15] are represented as:

$$m=\left[ \begin{matrix} m_{A} \\ m_{S} \\ m_{W} \\ m_{\mathrm{SE}} \end{matrix} \right], e=\left[ \begin{matrix} e_{A} \\ e_{S} \\ e_{w} \\ 0 \end{matrix} \right], \bar{K}=\left[ \begin{matrix} \text{-∑}k_{A} & k_{\text{SA}} & k_{\text{WA}} & \text{0} \\ k_{\text{AS}} & \text{-∑}k_{S} & 0 & 0 \\ k_{\text{AW}} & k_{\text{SW}} & \text{-∑}k_{W} & k_{\text{SEW}} \\ 0 & \text{0} & k_{\text{WSE}} & \text{-∑}k_{\mathrm{SE}} \end{matrix} \right]$$

$$m=\left[ \begin{matrix} m_{\mathrm{Afree}} \\ m_{\mathrm{Aagg}} \\ m_{\mathrm{Aatt}} \\ m_{\mathrm{Rfree}} \\ m_{\mathrm{Ragg}} \\ m_{\mathrm{Ratt}} \\ m_{\mathrm{Sfree}} \\ m_{\mathrm{Sagg}} \\ m_{\mathrm{Satt}} \\ m_{\mathrm{Wfree}} \\ m_{\mathrm{Wagg}} \\ m_{\mathrm{Watt}} \\ m_{\mathrm{SEfree}} \\ m_{\mathrm{SEagg}} \\ m_{\mathrm{SEatt}} \end{matrix} \right], e=\left[ \begin{matrix} e_{\mathrm{Afree}} \\ 0 \\ 0 \\ 0 \\ 0 \\ 0 \\ e_{\mathrm{Sfree}} \\ 0 \\ 0 \\ e_{\mathrm{Wfree}} \\ 0 \\ 0 \\ 0 \\ 0 \\ 0 \end{matrix} \right], \bar{I}=\left[ \begin{matrix} \text{-(∑}\text{k}_{\text{Afree}}\text{)} & \text{0} & \text{0} & \text{0} & \text{0} & \text{0} & \text{0} & \text{0} & \text{0} & \text{0} & \text{0} & \text{0} & \text{0} & \text{0} & \text{0} \\ \text{k}_{\text{aggA}} & \text{-(∑}\text{k}_{\text{Aagg}}\text{)} & \text{0} & \text{0} & \text{0} & \text{0} & \text{0} & \text{0} & \text{0} & \text{0} & \text{0} & \text{0} & \text{0} & \text{0} & \text{0} \\ \text{k}_{\text{attA}} & \text{0} & \text{-(∑}\text{k}_{\text{Aatt}}\text{)} & \text{0} & \text{0} & \text{0} & \text{0} & \text{0} & \text{0} & \text{0} & \text{0} & \text{0} & \text{0} & \text{0} & \text{0} \\ \text{k}_{\text{ARfree}} & \text{0} & \text{0} & \text{-(∑}\text{k}_{\text{Rfree}}\text{)} & \text{0} & \text{0} & \text{0} & \text{0} & \text{0} & \text{0} & \text{0} & \text{0} & \text{0} & \text{0} & \text{0} \\ \text{0} & \text{k}_{\text{ARagg}} & \text{0} & \text{0} & \text{-(∑}\text{k}_{\text{Ragg}}\text{)} & \text{0} & \text{0} & \text{0} & \text{0} & \text{0} & \text{0} & \text{0} & \text{0} & \text{0} & \text{0} \\ \text{0} & \text{0} & \text{k}_{\text{ARatt}} & \text{0} & \text{0} & \text{-(∑}\text{k}_{\text{Ratt}}\text{)} & \text{0} & \text{0} & \text{0} & \text{0} & \text{0} & \text{0} & \text{0} & \text{0} & \text{0} \\ \text{k}_{\text{depASfree}} & \text{0} & \text{0} & \text{k}_{\text{depRSfree}} & \text{0} & \text{0} & \text{-(∑}\text{k}_{\text{Sfree}}\text{)} & \text{0} & \text{0} & \text{0} & \text{0} & \text{0} & \text{0} & \text{0} & \text{0} \\ \text{0} & \text{k}_{\text{depASagg}} & \text{0} & \text{0} & \text{k}_{\text{depRSagg}} & \text{0} & \text{k}_{\text{aggS}} & \text{-(∑}\text{k}_{\text{Sagg}}\text{)} & \text{0} & \text{0} & \text{0} & \text{0} & \text{0} & \text{0} & \text{0} \\ \text{0} & \text{0} & \text{k}_{\text{depASatt}} & \text{0} & \text{0} & \text{k}_{\text{depRSatt}} & \text{k}_{\text{attS}} & \text{0} & \text{-(∑}\text{k}_{\text{Satt}}\text{)} & \text{0} & \text{0} & \text{0} & \text{0} & \text{0} & \text{0} \\ \text{k}_{\text{depAWfree}} & \text{0} & \text{0} & \text{k}_{\text{depRWfree}} & \text{0} & \text{0} & \text{k}_{\text{runSWfree}} & \text{0} & \text{0} & \text{-(∑}\text{k}_{\text{Wfree}}\text{)} & \text{0} & \text{0} & \text{k}_{\text{rsSEWfree}} & \text{0} & \text{0} \\ \text{0} & \text{k}_{\text{depAWagg}} & \text{0} & \text{0} & \text{k}_{\text{depRWagg}} & \text{0} & \text{0} & \text{k}_{\text{runSWagg}} & \text{0} & \text{k}_{\text{aggW}} & \text{-(∑}\text{k}_{\text{Wagg}}\text{)} & \text{0} & \text{0} & \text{k}_{\text{rsSEWagg}} & \text{0} \\ \text{0} & \text{0} & \text{k}_{\text{depAWatt}} & \text{0} & \text{0} & \text{k}_{\text{depRWatt}} & \text{0} & \text{0} & \text{k}_{\text{eroSWatt}} & \text{k}_{\text{attW}} & \text{0} & \text{-(∑}\text{k}_{\text{Watt}}\text{)} & \text{0} & \text{0} & \text{k}_{\text{rsSEWatt}} \\ \text{0} & \text{0} & \text{0} & \text{0} & \text{0} & \text{0} & \text{0} & \text{0} & \text{0} & \text{k}_{\text{depWSEfree}} & \text{0} & \text{0} & \text{-(∑}\text{k}_{\text{SEfree}}\text{)} & \text{0} & \text{0} \\ \text{0} & \text{0} & \text{0} & \text{0} & \text{0} & \text{0} & \text{0} & \text{0} & \text{0} & \text{0} & \text{k}_{\text{depWSEagg}} & \text{0} & \text{k}_{\text{aggSE}} & \text{-(∑}\text{k}_{\text{SEagg}}\text{)} & \text{0} \\ \text{0} & \text{0} & \text{0} & \text{0} & \text{0} & \text{0} & \text{0} & \text{0} & \text{0} & \text{0} & \text{0} & \text{k}_{\text{depWSEatt}} & \text{k}_{\text{attSE}} & \text{0} & \text{-(∑}\text{k}_{\text{SEatt}}\text{)} \end{matrix} \right]$$

Subscripts A, R, S, W, and SE denote the environmental compartments of air, rain, agricultural soil, freshwater, and sediment, respectively. In the context of nano-IMI, the subscripts free, agg, and att indicate its free, aggregated, and attached forms, respectively. The subscripts dep, run, ero, bur, and rs correspond to deposition, runoff, erosion, burial, and resuspension, respectively. The subscript order signifies the transport of substances between compartments; for instance, AW indicates the transport of chemicals from the air to the freshwater compartment.

**Collection of measured environmental concentration (MEC) of IMI**

The global surface water concentrations of imidacloprid were obtained from published articles [14]. Additionally, we categorized the measured environmental concentration (MEC) of IMI into two groups based on the measurement area: agricultural regions and surface water. Any MEC of IMI that did not specify the measurement area was classified into the surface water category.

**Text S3:** **Freshwater effect factor, fate factor, and exposure factor of IMI and nano-IMI.**

3.1 Effect factor

The calculation of Ecotoxicity Factors (*EF*) should rely on the Species Sensitivity Distribution (SSD) to achieve the Potentially Affected Fraction (PAF) value [15]. According to USEtox, it is advisable to incorporate chronic or sub-chronic toxicity data from three distinct freshwater aquatic species, such as algae, crustaceans, and fish, representing three trophic levels. To transition from acute to chronic toxicity data, USEtox guidelines recommend using an acute-to-chronic ratio (ACR) of 2. However, research by Smit et al. has shown that the ACR for IMI varies significantly, ranging from 16 to 143 [16]; therefore, using the default ACR of 2 could lead to substantial deviations. Here, 90.9 was selected as the ACR of IMI based on the guideline values of neonicotinoid insecticides established previously [17]. For nano-IMI, an ACR of 2 is appropriate, as suggested by our own acute versus chronic toxicity tests. Nano-IMI's ecotoxicological data are compiled in Table S26.

3.2 Fate factor

The fate matrix $\bar{FF}$ is defined as the negative inverse of the transfer rate coefficient matrix [18], where this matrix's elements represent the fate factor (*FF*) of chemicals in each environmental compartment. Consequently, the fate factors for IMI and nano-IMI are computed using equations (S1) and (S2), respectively.

$\bar{FF}_{\mathrm{IMI}}=-\bar{K}^{-1}=\left[ \begin{matrix} {FF}_{\mathrm{AA}} & {FF}_{\mathrm{AS}} & {FF}_{\mathrm{AW}} & {FF}_{\mathrm{ASE}} \\ {FF}_{\mathrm{SA}} & {FF}_{\mathrm{SS}} & {FF}_{\mathrm{SW}} & {FF}_{\mathrm{SSE}} \\ {FF}_{\mathrm{WA}} & {FF}_{\mathrm{WS}} & {FF}_{\mathrm{WW}} & {FF}_{\mathrm{WSE}} \\ {FF}_{\mathrm{SEA}} & {FF}_{\mathrm{SES}} & {FF}_{\mathrm{SEW}} & {FF}_{\mathrm{SESE}} \end{matrix} \right]$ S1

$\bar{FF}_{nano-IMI}=-\bar{I}^{-1}=\left[ \begin{matrix} \ldots& \ldots& \ldots& \ldots\\ \ldots& {FF}_{WW,free} & \ldots& \ldots\\ \ldots& \ldots& {FF}_{WW,agg} & \ldots\\ \ldots& \ldots& \ldots& {FF}_{WW,att} \end{matrix} \right]$ S2

The entirety of the matrix $\bar{FF}_{\mathrm{IMI}}$ and $\bar{FF}_{nano-IMI}$ are presented in Supplementary material _2 and _3, respectively, whereas the freshwater *FF* of IMI and nano-IMI are summarized in Table S31.

3.3 Exposure factor

The exposure factor (*XF*) of nano-IMI under S1 and S2 can be derived from equations (S3) and (S4), respectively [19].

$\text{XF}_{\text{S}\text{1}}\text{ = }\frac{\text{m}_{\text{free}}}{\text{m}_{\text{free}}\text{ }\text{+}\text{ }\text{m}_{\text{Biota}}}\text{ }\text{=}\text{ }\frac{\text{1}}{\text{1}\text{ }\text{+}\text{ }\frac{\text{m}_{\text{Biota}}}{\text{m}_{\text{free}}}}\text{ }\text{= }\frac{\text{1}}{\text{1}\text{ }\text{+}\text{ }\mathrm{BCF}_{\mathrm{free}}\text{ }\text{*}\text{ }\text{[Biota]}}$ S3

$\text{XF}_{\text{S2}}\text{ = }\frac{\text{m}_{\text{free}}\text{ }\text{+}\text{ }\text{m}_{\text{agg}}\text{ }\text{+}\text{ }\text{m}_{\text{att}}}{\text{m}_{\text{free}}\text{ }\text{+}{\text{ }\text{m}}_{\text{agg}}\text{ }\text{+}\text{ }\text{m}_{\text{att}}\text{ }\text{+}\text{ }\text{m}_{\text{Biota}}}$ $\text{= }\frac{\text{1}}{\text{1}\text{ }\text{+}\text{ }\frac{\text{m}_{\text{Biota}}}{\text{m}_{\text{free}}\text{ }\text{+}{\text{ }\text{m}}_{\text{agg}}\text{ }\text{+}\text{ }\text{m}_{\text{att}}}}$ $\text{= }\frac{\text{1}}{\text{1}\text{ }\text{+}\mathrm{BCF}_{free+agg+att}\text{ }\text{*}\text{ }\text{[Biota]}}$ S4

The variables m_free_, m_agg_, and m_att_ denote the masses of nano-IMI in its free, aggregated, and attached forms within freshwater compartments. m_Biota_ refers to the mass of nano-IMI accumulated by biota. BCF and [Biota] signify the bioaccumulation factors for nano-IMI and the population concentrations of organisms in freshwater, respectively. The *XF*s of nano-IMI are available in Table S33.

**TABLES**

Table S1. Inputs/outputs to produce isobutylene [20].

| Yield 94.8 % | Section | Material | Amount | Unit | Corresponding LCI | Database | Comments |
| --- | --- | --- | --- | --- | --- | --- | --- |
| 9.27 g  isobutylene | Input | Isobutanol | 12.91 | g | {RER}  \| market for isobutanol \| APOS, U | Ecoinvent 3 | 16.08 mL |
|  |  | Aluminium oxide | 24 | g | {RNA}\| aluminium oxide production \| APOS, U | Ecoinvent 3 | Dehydration catalyst |
|  |  | Nitrogen | 2.06 | g | liquid {CA-QC} \| air separation, cryogenic \| APOS, U | Ecoinvent 3 | Diluent gas (Density: 0.00125 g/cm^3^) |
|  |  | Electricity | 41.39 | kJ | low voltage {Canada without Quebec} \| market group for \| APOS, U | Ecoinvent 3 | 200 ℃ |
|  | Output | Nitrogen, atmospheric | 2.06 | g | Emissions to air | - | N_2_ |
|  |  | Water, CA | 2.97 | cm^3^ | Emissions to water | - | - |
|  |  | Catalyst waste | 24 | g | Final waste flows | - | Al_2_O_3_ |

Table S2. Inputs/outputs to produce tert-butanol [21].

| Yield 98.68 % | Section | Material | Amount | Unit | Corresponding LCI | Database | Comments |
| --- | --- | --- | --- | --- | --- | --- | --- |
| 73.14 g  tert-butanol | Input | Deionized water | 20 | g | from tap water, at user {Europe without Switzerland} \| market for water, deionised, from tap water, at user \| APOS, U | Ecoinvent 3 | - |
|  |  | Isobutylene | 56.11 | g | Created (table S1) | - | - |
|  |  | Anionic resin | 10 | g | {RER}\| market for anionic resin \| APOS, U | Ecoinvent 3 | Catalyst |
|  |  | Electricity | 252.7 | kJ | low voltage {Canada without Quebec} \| market group for \| APOS, U | Ecoinvent 3 | 85 ℃ |
|  | Output | Water, CA | 2.98 | cm^3^ | Emissions to water | - | - |
|  |  | Catalyst waste | 10 | g | Final waste flows | - | Anionic resin |

Table S3. Inputs/outputs to produce sodium tert-butoxide [22].

| Yield 99 % | Section | Material | Amount | Unit | Corresponding LCI | Database | Comments |
| --- | --- | --- | --- | --- | --- | --- | --- |
| 23.8 g  sodium tert-butoxide | Input | Deionized Water | 4.46 | g | from tap water, at user {Europe without Switzerland} \| market for water, deionised, from tap water, at user \| APOS, U | Ecoinvent 3 | - |
|  |  | Sodium amide | 9.8 | g | {RER}\| market for sodium amide \| APOS, U | Ecoinvent 3 | - |
|  |  | tert-Butanol | 19 | g | Created (table S2) | - | - |
|  |  | Toluene | 209.3 | g | liquid {RER}\| market for toluene, liquid \| APOS, U | Ecoinvent 3 | 240 mL |
|  |  | Electricity | 673.9 | kJ | low voltage {Canada without Quebec} \| market group for \| APOS, U | Ecoinvent 3 | 70-110 ℃ |
|  |  | Electricity | 43.2 | kJ | low voltage {Canada without Quebec} \| market group for \| APOS, U | Ecoinvent 3 | Distillation |
|  | Output | Ammonium, ion | 8.68 | g | Emissions to water | - | Ammonia water |
|  |  | Waste water | 210.1 | cm^3^ | average {Europe without Switzerland}\| market for wastewater, average \| APOS, U | Ecoinvent 3 | Toluene |

Table S4. Inputs/outputs to produce 2,3-dichloropropionaldehyde (CAS: 10140-89-3) [23].

| Yield 89 % | Section | | Material | | Amount | | Unit | | Corresponding LCI | Database | | Comments |  |
| --- | --- | --- | --- | --- | --- | --- | --- | --- | --- | --- | --- | --- | --- |
| 41 g  2,3-dichloro-propionaldehyde | |  | Input | | Acrolein | | 20 | | g | {GLO}\| market for \| APOS, U | | Ecoinvent 3 | 0.357 mol |
|  |  |  | Ethylene dichloride | | 100 | | g | | {RER}\| market for ethylene dichloride \| APOS, U | Ecoinvent 3 | | - |  |
|  |  |  | Chlorine | | 25 | | g | | gaseous {CA-QC} \| chlor-alkali electrolysis, membrane cell \| APOS, U | Ecoinvent 3 | | 0.353 mol |  |
|  |  |  | Electricity | | 151.2 | | kJ | | low voltage {Canada without Quebec} \| market group for \| APOS, U | Ecoinvent 3 | | -1-0 ℃ |  |
|  |  | Electricity | | 7.2 | | kJ | | low voltage {Canada without Quebec} \| market group for \| APOS, U | Ecoinvent 3 | | Reduction vaporization |  |  |
|  |  | Output | | Chlorine | | 2.1 | | g | | Emissions to air | - | | - |
|  |  |  |  | Waste water | | 101.9 | | cm^3^ | | average {Europe without Switzerland}\| market for wastewater, average \| APOS, U | Ecoinvent 3 | | Ethylene dichloride |

Table S5. Inputs/outputs to produce 4-chloro-4-(chloromethyl)-5-oxopentanenitrile (CCC, CAS: 150807-86-6) [23].

| Yield 79.4 % | Section | Material | Amount | Unit | Corresponding LCI | | Database | | Comments |
| --- | --- | --- | --- | --- | --- | --- | --- | --- | --- |
| 47 g  CCC | Input | 2,3-dichloro-propionaldehyde | 40 | g | Created (table S4) | - | | - | |
|  |  | Acrylonitrile | 25 | g | {GLO}\| market for \| APOS, U | Ecoinvent 3 | | - | |
|  |  | Xylene | 172 | g | {RER}\| market for xylene \| APOS, U | Ecoinvent 3 | | 200 mL; density: 0.86 g/cm3 | |
|  |  | Sodium hydroxide | 1 | g | without water, in 50% solution state {CA-QC} \| chlor-alkali electrolysis, membrane cell \| APOS, U | Ecoinvent 3 | | Amount: 0.5/50% = 1 g | |
|  |  | Sodium tert-butoxide | 0.69 | g | Created (table S3) | - | | Catalyst | |
|  |  | Hydrochloric acid | 2.4 | g | without water, in 30% solution state {CA-QC} \| hydrochloric acid production, from the reaction of hydrogen with chlorine \| APOS, U | Ecoinvent 3 | | Neutralizing catalyst;  Amount: 0.72/30% = 2.4 g | |
|  |  | Sodium chloride | 59.95 | g | brine solution {GLO}\| market for \| APOS, U | Ecoinvent 3 | | Detergent (50 mL; density: 1.199 g/cm^3^) | |
|  |  | Electricity | 1011 | kJ | low voltage {Canada without Quebec} \| market group for \| APOS, U | Ecoinvent 3 | | 25-30 ℃ | |
|  |  | Electricity | 22.5 | kJ | low voltage {Canada without Quebec} \| market group for \| APOS, U | Ecoinvent 3 | | Reduction vaporization | |
|  | Output | Sodium chloride | 64.03 | g | Emissions to water | - | | - | |
|  |  | Waste water | 190 | cm^3^ | average {Europe without Switzerland}\| market for wastewater, average \| APOS, U | Ecoinvent 3 | | Xylene | |

Table S6. Inputs/outputs to produce 2-chloro-5-chloromethyl-pyridine (CAS: 70258-18-3) [1].

| Yield 91.2 % | Section | Material | Amount | Unit | Corresponding LCI | Database | Comments |
| --- | --- | --- | --- | --- | --- | --- | --- |
| 14.9 g  2-chloro-5-chloromethyl-pyridine | Input | N, N-dimethyl-formamide | 3.65 | g | {GLO}\| market for \| APOS, U | Ecoinvent 3 | 0.05 mol |
|  |  | Phosgene | 10.88 | g | liquid {RER}\| market for \| APOS, U | Ecoinvent 3 | - |
|  |  | Toluene | 10.14 | g | liquid {RER}\| market for toluene, liquid \| APOS, U | Ecoinvent 3 | 0.11 mol |
|  |  | Nitrogen | 3.08 | g | liquid {CA-QC} \| air separation, cryogenic \| APOS, U | Ecoinvent 3 | - |
|  |  | CCC | 18 | g | Created (table S5) | - | 0.1 mol |
|  |  | Electricity | 98.28 | kJ | low voltage {Canada without Quebec} \| market group for \| APOS, U | Ecoinvent 3 | 30 ℃ |
|  |  | Electricity | 2.7 | kJ | low voltage {Canada without Quebec} \| market group for \| APOS, U | Ecoinvent 3 | Reduction vaporization |
|  |  | Electricity | 288 | kJ | low voltage {Canada without Quebec} \| market group for \| APOS, U | Ecoinvent 3 | Nitrogen stripping |
|  | Output | Nitrogen, atmospheric | 3.08 | g | Emissions to air | - | - |
|  |  | Carbon dioxide | 4.05 | g | Emissions to air | - | - |
|  |  | Waste water | 23.73 | cm^3^ | average {Europe without Switzerland}\| market for wastewater, average \| APOS, U | Ecoinvent 3 | Toluene |

Table S7. Inputs/outputs to produce 2-nitroaminoimidazoline (CAS: 5465-96-3) [2].

| Yield 96.1 % | Section | Material | Amount | Unit | Corresponding LCI | Database | Comments |
| --- | --- | --- | --- | --- | --- | --- | --- |
| 1.25 g  2-nitroamino-imidazoline | Input | Trichloromethane | 8.88 | g | {RER}\| market for trichloromethane \| APOS, U | Ecoinvent 3 | - |
|  |  | Ethylenediamine | 0.72 | g | {RER}\| market for ethylenediamine \| APOS, U | Ecoinvent 3 | - |
|  |  | Nitro-compound | 1.66 | g | {RER}\| production \| APOS, U | Ecoinvent 3 | Instead of Dimethyl N-nitroimidodithio-carbonate (CAS: 141972-53-4) |
|  |  | Diethyl ether | 35.72 | g | without water, in 99.95% solution state {GLO}\| diethyl ether production \| APOS, U | Ecoinvent 3 | Detergent |
|  |  | Electricity | 0.41 | kJ | low voltage {Canada without Quebec} \| market group for \| APOS, U | Ecoinvent 3 | Magnetic stirring |
|  |  | Electricity | 0.54 | kJ | low voltage {Canada without Quebec} \| market group for \| APOS, U | Ecoinvent 3 | Reduction vaporization |
|  | Output | Waste water | 45.73 | cm^3^ | average {Europe without Switzerland}\| market for wastewater, average \| APOS, U | Ecoinvent 3 | Chloroform |

Table S8. Inputs/outputs to produce the active ingredient of imidacloprid (IMI) [3].

| Yield 98.7 % | Section | Material | Amount | Unit | Corresponding LCI | Database | Comments |
| --- | --- | --- | --- | --- | --- | --- | --- |
| 68.53 g  IMI active ingredient | Input | Methyl ethyl ketone | 286 | g | {RER}\| market for methyl ethyl ketone \| APOS, U | Ecoinvent 3 | - |
|  |  | Sodium sulfate | 2.5 | g | anhydrite {RER}\| market for \| APOS, U | Ecoinvent 3 | Desiccant |
|  |  | Sodium hydroxide | 20 | g | without water, in 50% solution state {CA-QC} \| chlor-alkali electrolysis, membrane cell \| APOS, U | Ecoinvent 3 | pH = 8;  Amount: 10/50% = 20 g |
|  |  | Potassium carbonate | 5 | g | {GLO}\| market for \| APOS, U | Ecoinvent 3 | Deacidification |
|  |  | 2-nitroiminoimidazoline | 40 | g | Created (table S7) | - | - |
|  |  | 2-Chloro-5-chloromethyl pyridine | 44 | g | Created (table S6) | - | - |
|  |  | Electricity | 2190 | kJ | low voltage {Canada without Quebec} \| market group for \| APOS, U | Ecoinvent 3 | Reduction vaporization |
|  | Output | Carbon dioxide | 1.59 | g | Emissions to air | - | - |
|  |  | Sodium chloride | 35.68 | g | Emissions to water | - | - |
|  |  | Waste water | 291.7 | cm^3^ | average {Europe without Switzerland}\| market for wastewater, average \| APOS, U | Ecoinvent 3 | Methyl ethyl ketone |

Table S9. Inputs/outputs to produce potassium bisulfate [6].

|  | Section | Material | Amount | Unit | Corresponding LCI | Database | Comments |
| --- | --- | --- | --- | --- | --- | --- | --- |
| 136.2 g potassium bisulfate | Input | Sulfuric acid | 98.08 | g | {CA-QC} \| primary zinc production from concentrate \| APOS, U | Ecoinvent 3 | - |
|  |  | Potassium nitrate | 101.1 | g | {GLO}\| market for \| APOS, U | Ecoinvent 3 | - |
|  | Output | nitric acid | 63.01 | g | Avoided products; without water, in 50% solution state {RER}\| market for nitric acid, without water, in 50% solution state \| APOS, U | Ecoinvent 3 | - |

Table S10. Inputs/outputs to produce potassium persulfate [6].

|  | Section | Material | Amount | Unit | Corresponding LCI | Database | Comments |
| --- | --- | --- | --- | --- | --- | --- | --- |
| 270.32 g Potassium bisulfate | Input | Potassium bisulfate | 272.34 | g | Created (table S9) | - | - |
|  |  | Electricity | 42.45 | kJ | low voltage {Canada without Quebec} \| market group for \| APOS, U | Ecoinvent 3 | PEM Electrolyser |
|  | Output | Hydrogen | 2.02 | g | Avoided products; liquid {CA-QC} \| chlor-alkali electrolysis, membrane cell \| APOS, U | Ecoinvent 3 | - |

Table S11. Inputs/outputs to produce polyacrylic acid (PAA) [5].

| Yield 100 % [24] | Section | Material | Amount | Unit | Corresponding LCI | Database | Comments |
| --- | --- | --- | --- | --- | --- | --- | --- |
| 7.5 g  Polyacrylic acid | Input | Acrylic acid | 7.5 | g | {RER}\| market for acrylic acid \| APOS, U | Ecoinvent 3 | - |
|  |  | Aluminium oxide | 31.25 | g | {RNA}\| aluminium oxide production \| APOS, U | Ecoinvent 3 | Supported catalyst |
|  |  | Potassium persulfate | 0.375 | g | Created (table S10) | - | Initiator |
|  |  | Deionized Water | 50 | g | from tap water, at user {Europe without Switzerland} \| market for water, deionised, from tap water, at user \| APOS, U | Ecoinvent 3 | - |
|  |  | Electricity | 1392 | kJ | low voltage {Canada without Quebec} \| market group for \| APOS, U | Ecoinvent 3 | Heating for 16 h |
|  |  | Electricity | 33.94 | kJ | low voltage {Canada without Quebec} \| market group for \| APOS, U | Ecoinvent 3 | Heating 1 h |
|  | Output | Potassium compounds, unspecified | 0.375 | g | Emissions to water | - | Initiator |
|  |  | Water, CA | 50 | cm^3^ | Emissions to water | - | - |
|  |  | Catalyst waste | 31.25 | g | Final waste flows | - | Al_2_O_3_ |

Table S12. Inputs/outputs to produce polymer nanoparticles [4].

| Yield 97 % | Section | Material | Amount | Unit | Corresponding LCI | Database | Comments |
| --- | --- | --- | --- | --- | --- | --- | --- |
| 0.097 g  Polymer nanoparticles | Input | Deionized water | 100 | g | from tap water, at user {Europe without Switzerland} \| market for water, deionised, from tap water, at user \| APOS, U | Ecoinvent 3 | - |
|  |  | polyacrylic acid | 0.1 | g | Created (table S11) | - | - |
|  |  | Sodium hydroxide | 0.34 | mg | without water, in 50% solution state {CA-QC} \| chlor-alkali electrolysis, membrane cell \| APOS, U | Ecoinvent 3 | Adjust pH to 9.63; Amount: 0.17/50% = 0.34 mg |
|  |  | Sodium chloride | 11.1 | g | brine solution {GLO}\| market for \| APOS, U | Ecoinvent 3 | - |
|  |  | Electricity | 6.75 | kJ | low voltage {Canada without Quebec} \| market group for \| APOS, U | Ecoinvent 3 | Magnetic stirring |
|  |  | Electricity | 0.6 | kJ | low voltage {Canada without Quebec} \| market group for \| APOS, U | Ecoinvent 3 | UV lamp |
|  |  | Electricity | 1.42 | kJ | low voltage {Canada without Quebec} \| market group for \| APOS, U | Ecoinvent 3 | Freezer dryer |
|  | Output | Sodium chloride | 11.1 | g | Emissions to water | - | - |
|  |  | Water, CA | 100 | cm^3^ | Emissions to water | - | - |

Table S13. Inputs/outputs to produce the active ingredient (contain the nanocarrier) of nano-encapsulated imidacloprid (nano-IMI) [4].

| Load rate 91 % | Section | Material | Amount | Unit | Corresponding LCI | Database | Comments |
| --- | --- | --- | --- | --- | --- | --- | --- |
| 5.92 mg  nano-IMI ingredient | Input | Methanol | 3.16 | g | {GLO}\| market for \| APOS, U | Ecoinvent 3 | - |
|  |  | Polymer nanoparticles | 3.1 | mg | Created (table S12) | - | m_IMI_/m_carrier_=91% |
|  |  | IMI active ingredient | 28.5 | mg | Created (table S8) | - | Recycle 100% |
|  |  | Hydrochloric acid | 4.89 | mg | without water, in 30% solution state {CA-QC} \| hydrochloric acid production, from the reaction of hydrogen with chlorine \| APOS, U | Ecoinvent 3 | Adjust pH to 2 |
|  |  | Deionized water | 1 | g | from tap water, at user {Europe without Switzerland} \| market for water, deionised, from tap water, at user \| APOS, U | Ecoinvent 3 | - |
|  |  | Sodium hydroxide | 36 | ng | without water, in 50% solution state {CA-QC} \| chlor-alkali electrolysis, membrane cell \| APOS, U | Ecoinvent 3 | Adjust pH to 8 |
|  |  | Electricity | 4.11 | kJ | low voltage {Canada without Quebec} \| market group for \| APOS, U | Ecoinvent 3 | Whirl mixer |
|  |  | Electricity | 0.46 | J | low voltage {Canada without Quebec} \| market group for \| APOS, U | Ecoinvent 3 | Freezer dryer |
|  |  | Electricity | 0.9 | kJ | low voltage {Canada without Quebec} \| market group for \| APOS, U | Ecoinvent 3 | Evaporated methanol |
|  | Output | IMI active ingredient | 25.7 | mg | Avoided products; Created (table S8) | - | Recycle 100 % |
|  |  | Sodium chloride | 4.89 | mg | Emissions to water | - | - |
|  |  | Water, CA | 1 | cm^3^ | Emissions to water | - | - |
|  |  | Waste water | 3.16 | cm^3^ | average {Europe without Switzerland}\| market for wastewater, average \| APOS, U | Ecoinvent 3 | Methanol |

Table S14. Inputs/outputs to produce nonylphenol [25].

| Yield 96.84 % | Section | Material | Amount | Unit | Corresponding LCI | Database | Comments |
| --- | --- | --- | --- | --- | --- | --- | --- |
| 17.32 g  nonylphenol | Input | Fatty alcohol | 14.43 | g | {GLO}\| market for \| APOS, U | Ecoinvent 3 | Representative 1-Nonanol |
|  |  | Phenol | 9.41 | g | {RER}\| production \| APOS, U | Ecoinvent 3 | - |
|  |  | Iron (III) sulfate | 9.6 | g | without water, in 12.5% iron solution state {CA-QC} \| production \| APOS, U | Ecoinvent 3 | Representative sulfated Fe_2_O_3_;  Amount: 1.2/12.5% = 9.6 g |
|  |  | Sodium bicarbonate | 108 | g | {RER}\| soda production, solvay process \| APOS, U | Ecoinvent 3 | - |
|  |  | Naphtha | 77 | g | {RER}\| market for \| APOS, U | Ecoinvent 3 | Representative petroleum ether; density of 0.77g/cm^3^, assuming 100 mL is used |
|  |  | Deionized Water | 50 | g | from tap water, at user {Europe without Switzerland} \| market for water, deionised, from tap water, at user \| APOS, U | Ecoinvent 3 | - |
|  |  | Electricity | 889.2 | kJ | low voltage {Canada without Quebec} \| market group for \| APOS, U | Ecoinvent 3 | 80℃ and drying |
|  |  | Electricity | 18 | kJ | low voltage {Canada without Quebec} \| market group for \| APOS, U | Ecoinvent 3 | Reduction vaporization |
|  | Output | Water, CA | 50 | cm^3^ | Emissions to water | - | - |
|  |  | Sodium compounds, unspecified | 108 | g | Emissions to water | - | - |
|  |  | Catalyst waste | 1.2 | g | Final waste flows | - | sulfated Fe_2_O_3_ |
|  |  | Waste water | 91.92 | cm^3^ | average {Europe without Switzerland}\| market for wastewater, average \| APOS, U | Ecoinvent 3 | petroleum ether |

Table S15. Inputs/outputs to produce polyethylene glycol nonylphenyl ether (APNOHO, CAS: 9016-45-9) [11].

| Yield 98.7 % | Section | Material | Amount | Unit | Corresponding LCI | Database | Comments |
| --- | --- | --- | --- | --- | --- | --- | --- |
| 196.3 g  APNOHO | Input | Nonylphenol | 77 | g | Created (table S14) | - | - |
|  |  | Potassium hydroxide | 2.3 | g | {RER}\| production \| APOS, U | Ecoinvent 3 | - |
|  |  | Nitrogen | 1.88 | g | liquid {CA-QC} \| air separation, cryogenic \| APOS, U | Ecoinvent 3 | - |
|  |  | Propylene oxide | 127.6 | g | liquid {RER}\| market for propylene oxide, liquid \| APOS, U | Ecoinvent 3 | - |
|  |  | Electricity | 556.42 | kJ | low voltage {Canada without Quebec} \| market group for \| APOS, U | Ecoinvent 3 | 120 ℃ |
|  | Output | Nitrogen, atmospheric | 1.88 | g | Emissions to air | - | N2 |
|  |  | Waste water | 10.6 | cm^3^ | average {Europe without Switzerland}\| market for wastewater, average \| APOS, U | Ecoinvent 3 | - |

Table S16. Inputs/outputs to produce IMI.

|  | Section | Material | Amount | Unit | Corresponding LCI | Database | Comments |
| --- | --- | --- | --- | --- | --- | --- | --- |
| 303.22 g  IMI | Input | IMI active ingredient | 68.53 | g | Created (table S8) | - | Active ingredient: 22.6 % |
|  |  | APNOHO | 234.69 | g | Created (table S15) | - | Inert ingredients: 77.4 % |

Table S17. Inputs/outputs to produce nano-IMI.

|  | Section | Material | Amount | Unit | Corresponding LCI | Database | Comments |
| --- | --- | --- | --- | --- | --- | --- | --- |
| 15.25 mg  nano-IMI | Input | nano-IMI ingredient | 5.92 | mg | Created (table S13) | - | Active ingredient: 18.50 %  Nanocarrier: 18.5%/91%=20.33 % |
|  |  | APNOHO | 9.33 | mg | Created (table S15) | - | Inert ingredients:1-18.5%-20.33%=61.17 % |

Table S18. Calculations and assumptions for energy consumption during material syntheses.

| Product | Instrumentation | Specification | Using time | Amount of reagent | Computational formula | Value (kJ) | Comment |
| --- | --- | --- | --- | --- | --- | --- | --- |
| isobutylene | Series 4568 Mini Reactor [26] | 600 mL; include Cooling Coil; heater power: 780 w | 1 h | 16.08 mL | (0.78*0.5+0.1*0.78*0.5) *3600*16.08/600 | 41.39 | Isobutanol evaporates at 200 ℃; Assumption: Holding time 0.5 h |
| tert-Butanol | Series 4568 Mini Reactor [26] | 600 mL; include Cooling Coil; heater power: 780 w | 1.5 h | 90 mL | (0.78*0.5+0.1*0.78*1) *3600*90/600 | 252.72 | 85 ℃; Assumption: Holding time 1 h |
| sodium tert-butoxide | Series 4568 Mini Reactor [26] | 600 mL; include Cooling Coil; heater power: 780 w | 1.5 h | 240 mL | (0.78*0.5+0.1*0.78*1) *3600*240/600 | 673.92 | 70-110 ℃; Assumption: Holding time 1 h |
|  | IKA Rotary Evaporators  RV 10 auto pro V [27] | Filling volume max: 4 L; Power input: 100 w | 2 h | 240 mL | 0.1*2*3600*240/4000 | 43.2 | Atmospheric distillation: 1 h; Decompress distillation: 1 h |
| 2,3-dichloro-propionaldehyde | DHJF-8002 Low-temperature Stirring Reaction Bath [28] | Maximum flask capacity: 2 L; Heating power: 1500 w | 2.5 h | 80 mL | (1.5*0.5+0.1*1.5*2) *3600*80/2000 | 151.2 | -1-0 ℃; Assumption: Holding time 2 h |
|  | IKA Rotary Evaporators  RV 10 auto pro V [27] | Filling volume max: 4 L; Power input: 100 w | 1 h | 80 mL | 0.1*1*3600*80/4000 | 7.2 | Decompress vaporization |
| CCC | Series 4568 Mini Reactor [26] | 600 mL; include Cooling Coil; heater power: 780 w | 6.3 h | 200 mL | (0.78*0.5+0.1*0.78*5.8) *3600*200/600 | 1010.88 | 25-30 ℃; Assumption: Holding time 5.8 h |
|  | IKA Rotary Evaporators  RV 10 auto pro V [27] | Filling volume max: 4 L; Power input: 100 w | 1 h | 250 mL | 0.1*1*3600*250/4000 | 22.5 | Decompress vaporization |
| 2-chloro-5-chloromethyl-pyridine | Series 4568 Mini Reactor [26] | 600 mL; include Cooling Coil; heater power: 780 w | 2.5 h | 30 mL | (0.78*0.5+0.1*0.78*2) *3600*30/600 | 98.28 | 30 ℃; Assumption: Holding time 2 h |
|  | IKA Rotary Evaporators  RV 10 auto pro V [27] | Filling volume max: 4 L; Power input: 100 w | 1 h | 30 mL | 0.1*1*3600*30/4000 | 2.7 | Decompress vaporization |
|  | NDK200-1N Sample  Concentrator [29] | Power: 200 w | 2 h | 2 L (gas) | 0.2*2*3600*2/10 | 288 |  |
| 2-nitroamino-imidazoline | CAPPRondo Magnetic  Stirrer CRS-15X [30] | Stirring Quantity Max: 800 mL; Power consumption: 5 w | 3 h | 6 mL | 0.005*3*3600*6/800 | 0.405 | Magnetic stirring; |
|  | IKA Rotary Evaporators  RV 10 auto pro V [27] | Filling volume max: 4 L; Power input: 100 w | 1 h | 6 mL | 0.1*1*3600*6/4000 | 0.54 | Decompress vaporization |
| IMI active ingredient | Series 4568 Mini Reactor [26] | 600 mL; include Cooling Coil; heater power: 780 w | 8.5 h | 360 mL | (0.78*0.5+0.1*0.78*8) *3600*30/600 | 2190.24 | 40-50 ℃; Assumption: Holding time 8 h |
| potassium persulfate | AEM Electrolyser EL 4.0 [31] | Nominal power consumption per Nm³ of H₂ produced: 4.8 kWh/Nm³ | - | 0.00246 m^3^  (H_2_ gas) | 0.00246*4.8*3600 | 42.45 | Mass of H_2_: 2.02 g; Density of H_2_: 0.82218 kg/m^3^ |
| polyacrylic acid | Parallel & Scale-Up Microwave Synthesizer - MARS 6 Synthesis [32] | Power: 1800 W; Minimum Ramp Time: 20 min; Maximum Working Volume (per vessel): 12*75 = 900 mL | 16.3 h | 100 mL | (1.8*20/60+0.1*1.8*16) *3600*100/900 | 1392 | Microwave heating; Assumption: Holding time 16 h |
|  |  | Power: 1800W; Minimum Ramp Time: 5 min; Maximum Working Volume (Open Vessel): 5*70% = 3.5 L | 1.08 h | 100 mL | (1.8*5/60+0.1*1.8*1) *3600*100/3500 | 33.94 | Microwave heating |
| polymer nanoparticles | CAPPRondo Magnetic Stirrer  CRS-15X [30] | Stirring Quantity Max: 800 mL; Power consumption: 5 w | 3 h | 100 mL | 0.005*3*3600*100/800 | 6.75 | Magnetic stirring |
|  | Thermo Scientific™ 3UV Lamp [33] | Wattage: 8 w | 5min | 100 mL | 0.008*5/60*3600*100/400 | 0.6 | Assumption: Maximum exposure volume: 400 mL |
|  | FREEZE DRYERS [34] | Refrigeration system: 1.02 kW; Max performance: 6 kg/24 h | 24 h | 0.097 g | 1.02*24*3600*0.097/6000 | 1.43 | Freeze drying |
| nano-IMI ingredient | MULTI-TX5 Digital Multi-tube Vortex [35] | Power: 40 W | 2 h | 10 mL | 0.04*2*3600*10/700 | 4.11 | Assumption: Maximum capacity: 14*50 = 700 mL |
|  | FREEZE DRYERS [34] | Refrigeration system: 1.02 kW; Max performance: 6 kg/24 h | 24 h | 26 mg | 1.02*24*3600*31.6*0.001/6000 | 0.46 |  |
|  | IKA Rotary Evaporators  RV 10 auto pro V [27] | Filling volume max: 4 L; Power input: 100 w | 1 h | 10 mL | 0.1*1*3600*10/4000 | 0.9 |  |
| nonylphenol | Series 4568 Mini Reactor [26] | 600 mL; include Cooling Coil; heater power: 780 w | 14.5 h | 100 mL | (0.78*0.5+0.1*0.78*14) *3600*100/600 | 889.2 | Reaction at 80℃ for 4 h, drying for 10 h |
|  | IKA Rotary Evaporators  RV 10 auto pro V [27] | Filling volume max: 4 L; Power input: 100 w | 2 h | 100 mL | 0.1*2*3600*100/4000 | 18 | Atmospheric distillation: 1 h; Decompress distillation: 1 h |
| APNOHO | WHF－0.5 L high magnetic  driven autoclave [36] | Power: 92 w; Volume: 0.5 L | 3.5 h | 240 mL | 0.092*3.5*3600*240/500 | 556.42 | Reaction at 120 ℃ for 2 h; Assumption: Holding time 1 h |

Assumption: For instruments lacking specific instructions on heating time or holding power, the heating time of 0.5 h and the holding power equal to 10% of the heating power are applied.

Table S19. TRACI characterization results for IMI and nano-IMI production under different functional units.

| Impact category | unit | 1 kg of pesticides production | | Annual per-hectare quantity of pesticide applied | |
| --- | --- | --- | --- | --- | --- |
|  |  | IMI | nano-IMI | IMI | nano-IMI |
| Ozone depletion | kg CFC-11 eq | 7.05E-04 | 5.80E-04 | 1.50E-03 | 1.33E-03 |
| Global warming | kg CO_2_ eq | 43.8 | 337 | 93 | 776 |
| Smog | kg O_3_ eq | 2.89 | 7.45 | 6.14 | 17.1 |
| Acidification | kg SO_2_ eq | 0.317 | 1.54 | 0.673 | 3.54 |
| Eutrophication | kg N eq | 0.223 | 0.588 | 0.473 | 1.35 |
| Carcinogenics | CTUh | 3.07E-06 | 9.47E-06 | 6.53E-06 | 2.18E-05 |
| Non carcinogenics | CTUh | 2.31E-05 | 5.09E-05 | 4.90E-05 | 1.17E-04 |
| Respiratory effects | kg PM_2.5_ eq | 0.047 | 0.125 | 0.100 | 0.289 |
| Ecotoxicity | CTUe | 566 | 2122 | 1202 | 4885 |
| Fossil fuel depletion | MJ surplus | 107 | 1320 | 228 | 3038 |

Table S20. Sensitivity analysis for IMI production. Reported numbers are sensitivity factors. (Red color highlights sensitive parameters)

| Impact category | OD | GW | PS | AC | EU | HHC | HHNC | RE | EC | FF |
| --- | --- | --- | --- | --- | --- | --- | --- | --- | --- | --- |
| 2-nitroimino-imidazoline | 9.95E-01 | 7.05E-01 | 7.10E-01 | 7.80E-01 | 6.57E-01 | 6.96E-01 | 7.80E-01 | 8.11E-01 | 6.54E-01 | 6.14E-01 |
| 2-chloro-5-chloro-methylpyridine | 6.54E-04 | 7.15E-02 | 1.00E-01 | 3.69E-02 | 6.31E-02 | 6.21E-02 | 3.43E-02 | 2.91E-02 | 6.76E-02 | 1.05E-01 |
| APNOHO | 3.69E-03 | 1.71E-01 | 1.58E-01 | 1.58E-01 | 2.33E-01 | 1.98E-01 | 1.60E-01 | 1.41E-01 | 2.24E-01 | 2.05E-01 |
| Methyl ethyl ketone | 1.93E-04 | 3.64E-02 | 2.06E-02 | 1.51E-02 | 1.72E-02 | 1.87E-02 | 1.11E-02 | 1.06E-02 | 1.29E-02 | 7.07E-02 |
| Energy | 4.87E-05 | 1.45E-02 | 8.68E-03 | 8.29E-03 | 2.79E-02 | 2.31E-02 | 1.24E-02 | 6.38E-03 | 3.96E-02 | 4.72E-03 |
| Synthetic stage | 0.00E+00 | 1.20E-04 | 0.00E+00 | 0.00E+00 | 0.00E+00 | 0.00E+00 | 0.00E+00 | 0.00E+00 | 0.00E+00 | 0.00E+00 |
| Sodium sulfate | 1.35E-06 | 1.37E-04 | 1.41E-04 | 1.75E-04 | 1.36E-04 | 1.68E-04 | 1.77E-04 | 1.29E-04 | 1.48E-04 | 1.09E-04 |
| Sodium hydroxide | 7.25E-05 | 4.88E-04 | 5.21E-04 | 4.00E-04 | 5.78E-04 | 8.46E-04 | 8.25E-04 | 5.77E-04 | 8.04E-04 | 1.76E-04 |
| Potassium carbonate | 5.87E-06 | 1.12E-03 | 1.13E-03 | 7.12E-04 | 8.81E-04 | 1.07E-03 | 8.29E-04 | 1.21E-03 | 8.57E-04 | 4.90E-04 |
| Waste water treated | 5.90E-08 | 1.09E-05 | 1.46E-05 | 1.38E-05 | 1.35E-04 | 5.40E-05 | 1.26E-04 | 1.47E-05 | 2.25E-05 | 3.95E-06 |

Table S21. Sensitivity analysis for nano-IMI production. Reported numbers are sensitivity factors (SFs). (red cells indicate sensitive parameters)

| Impact category | OD | GW | PS | AC | EU | HHC | HHNC | RE | EC | FF |
| --- | --- | --- | --- | --- | --- | --- | --- | --- | --- | --- |
| IMI | 9.91E-01 | 8.83E-02 | 2.68E-01 | 1.42E-01 | 2.43E-01 | 2.15E-01 | 3.19E-01 | 2.64E-01 | 1.70E-01 | 5.28E-02 |
| Methanol | 3.99E-07 | 7.71E-01 | 4.50E-01 | 7.16E-01 | 1.83E-02 | 9.07E-02 | 1.94E-01 | 5.14E-01 | 9.78E-02 | 9.07E-01 |
| Polymer nanoparticles | 1.65E-03 | 2.87E-02 | 6.35E-02 | 3.06E-02 | 1.39E-01 | 2.68E-01 | 1.51E-01 | 6.01E-02 | 1.57E-01 | 7.63E-03 |
| Electricity | 2.94E-03 | 9.40E-02 | 1.68E-01 | 8.50E-02 | 5.37E-01 | 3.76E-01 | 2.86E-01 | 1.19E-01 | 5.27E-01 | 1.91E-02 |
| APNOHO | 3.54E-03 | 1.76E-02 | 4.86E-02 | 2.57E-02 | 7.13E-02 | 5.11E-02 | 5.87E-02 | 4.20E-02 | 4.74E-02 | 1.32E-02 |
| Wastewater treatment | 1.55E-05 | 3.06E-04 | 1.23E-03 | 6.13E-04 | 1.13E-02 | 3.81E-03 | 1.26E-02 | 1.19E-03 | 1.30E-03 | 6.92E-05 |
| Hydrochloric acid | 4.75E-04 | 4.15E-04 | 2.87E-03 | 5.62E-04 | 1.68E-03 | 2.17E-03 | 3.17E-03 | 1.48E-03 | 1.77E-03 | 9.22E-05 |
| Deionized water | 4.79E-05 | 1.77E-04 | 4.98E-04 | 2.10E-04 | 6.65E-04 | 1.65E-03 | 6.95E-04 | 4.80E-04 | 3.98E-04 | 3.90E-05 |
| Sodium hydroxide | 3.15E-09 | 2.27E-09 | 7.25E-09 | 2.95E-09 | 8.01E-09 | 9.91E-09 | 1.37E-08 | 7.76E-09 | 7.70E-09 | 5.11E-10 |

Table S22. The emission rate of IMI and nano-IMI in multicompartment (vector *e*).

|  | Calculation | e vector (g·s^−1^) | |
| --- | --- | --- | --- |
| IMI | *e*_S_ = IMI active ingredient application rate × Area_S_  = 0.48 kg/ha/y × 2.23E+08 ha = 1.07E+08 kg/y = 3.39E+03 g/s | $e=\left[ \begin{matrix} e_{A} \\ e_{S} \\ \begin{matrix} e_{W} \\ e_{\mathrm{SE}} \end{matrix} \end{matrix} \right]$ | $e\text{ }\text{=}\left[ \begin{matrix} \text{0} \\ \text{3.39E+03} \\ \begin{matrix} \text{0} \\ \text{0} \end{matrix} \end{matrix} \right]$ |
| nano-IMI | nano-IMI active ingredient application rate (contain nanocarrier)  = 0.43 kg/ha/y ÷ 18.5% × (18.5% + 20.33%) = 0.89 kg/ha/y  *e*_S_ = nano-IMI active ingredient application rate (contain nanocarrier)× Area_S_  = 0.89 kg/ha/y × 2.23E+08 ha = 1.99E+08 kg/y = 6.32E+03 g/s | $e=\left[ \begin{matrix} e_{\mathrm{Afree}} \\ 0 \\ \begin{matrix} 0 \\ 0 \\ \begin{matrix} 0 \\ 0 \\ \begin{matrix} e_{\mathrm{Sfree}} \\ 0 \\ \begin{matrix} 0 \\ e_{\mathrm{Wfree}} \\ \begin{matrix} 0 \\ 0 \\ \begin{matrix} 0 \\ 0 \\ 0 \end{matrix} \end{matrix} \end{matrix} \end{matrix} \end{matrix} \end{matrix} \end{matrix} \right]$ | $e\text{ }\text{=}\left[ \begin{matrix} \text{0} \\ \text{0} \\ \begin{matrix} \text{0} \\ \text{0} \\ \begin{matrix} \text{0} \\ \text{0} \\ \begin{matrix} \text{6.32E+03} \\ \text{0} \\ \begin{matrix} \text{0} \\ \text{0} \\ \begin{matrix} \text{0} \\ \text{0} \\ \begin{matrix} \text{0} \\ \text{0} \\ \text{0} \end{matrix} \end{matrix} \end{matrix} \end{matrix} \end{matrix} \end{matrix} \end{matrix} \right]$ |

Table S23. Multimedia distribution of IMI under three rainfall scenarios (R_1_: limited rainfall, R_2_: normal rainfall, R_3_: heavy rainfall). A: Air, S: Agricultural soil, W: Fresh water, SE: Sediment.

| Compartment | R_1_ | | R_2_ | | R_3_ | |
| --- | --- | --- | --- | --- | --- | --- |
|  | m (kg) | fraction (%) | m (kg) | fraction (%) | m (kg) | fraction (%) |
| A | 1.51E-10 | 3.15E-08 | 4.19E-11 | 8.73E-09 | 1.48E-11 | 3.09E-09 |
| S | 0.44 | 92.08 | 0.11 | 23.15 | 0.03 | 6.11 |
| W | 0.04 | 7.85 | 0.37 | 76.19 | 0.45 | 93.08 |
| SE | 3.27E-04 | 0.068 | 3.17E-03 | 0.66 | 3.88E-03 | 0.81 |

Table S24. Multimedia distribution of nano-IMI under three rainfall scenarios (R_1_, R_2_, R_3_) and three attachment coefficients (α_1_, α_2_, α_3_).

| Scenario | | A | | R | | S | | W | | SE | |
| --- | --- | --- | --- | --- | --- | --- | --- | --- | --- | --- | --- |
|  |  | m (kg) | fraction (%) | m (kg) | fraction (%) | m (kg) | fraction (%) | m (kg) | fraction (%) | m (kg) | fraction (%) |
| R1 | S1 α_1_ | 0 | 0 | 0 | 0 | 0.89 | 99.9992 | 7.35E-06 | 0.0008 | 7.15E-14 | 8.00E-12 |
|  | S1 α_2_ | 0 | 0 | 0 | 0 | 0.89 | 99.9997 | 2.51E-06 | 0.0003 | 5.44E-15 | 6.09E-13 |
|  | S1 α_3_ | 0 | 0 | 0 | 0 | 0.89 | 99.9997 | 2.76E-06 | 0.0003 | 7.58E-15 | 8.48E-13 |
|  | S2 α_1_ | 0 | 0 | 0 | 0 | 0.89 | 99.9924 | 6.81E-05 | 0.008 | 2.27E-07 | 2.54E-05 |
|  | S2 α_2_ | 0 | 0 | 0 | 0 | 0.89 | 99.9924 | 6.81E-05 | 0.008 | 2.29E-07 | 2.57E-05 |
|  | S2 α_3_ | 0 | 0 | 0 | 0 | 0.89 | 99.9924 | 6.81E-05 | 0.008 | 2.28E-07 | 2.56E-05 |
| R2 | S1 α_1_ | 0 | 0 | 0 | 0 | 0.89 | 99.97 | 2.83E-04 | 0.03 | 2.76E-12 | 3.08E-10 |
|  | S1 α_2_ | 0 | 0 | 0 | 0 | 0.89 | 99.99 | 9.69E-05 | 0.01 | 2.10E-13 | 2.35E-11 |
|  | S1 α_3_ | 0 | 0 | 0 | 0 | 0.89 | 99.99 | 1.06E-04 | 0.01 | 2.92E-13 | 3.27E-11 |
|  | S2 α_1_ | 0 | 0 | 0 | 0 | 0.89 | 99.71 | 0.0026 | 0.29 | 4.45E-06 | 4.98E-04 |
|  | S2 α_2_ | 0 | 0 | 0 | 0 | 0.89 | 99.71 | 0.0026 | 0.29 | 4.54E-06 | 5.08E-04 |
|  | S2 α_3_ | 0 | 0 | 0 | 0 | 0.89 | 99.71 | 0.0026 | 0.29 | 4.50E-06 | 5.04E-04 |
| R3 | S1 α_1_ | 0 | 0 | 0 | 0 | 0.89 | 99.85 | 0.0013 | 0.15 | 1.27E-11 | 1.43E-09 |
|  | S1 α_2_ | 0 | 0 | 0 | 0 | 0.89 | 99.95 | 0.0004 | 0.05 | 9.71E-13 | 1.09E-10 |
|  | S1 α_3_ | 0 | 0 | 0 | 0 | 0.89 | 99.94 | 0.0005 | 0.06 | 1.35E-12 | 1.51E-10 |
|  | S2 α_1_ | 0 | 0 | 0 | 0 | 0.88 | 98.68 | 0.0118 | 1.32 | 1.97E-05 | 0.0022 |
|  | S2 α_2_ | 0 | 0 | 0 | 0 | 0.88 | 98.68 | 0.0118 | 1.32 | 2.02E-05 | 0.0023 |
|  | S2 α_3_ | 0 | 0 | 0 | 0 | 0.88 | 98.68 | 0.0118 | 1.32 | 2.00E-05 | 0.0022 |

Table S25. Predicted environmental concentrations (PEC) of IMI and nano-IMI in the freshwater compartment.

| Rainfall scenario | PEC_IMI_ (μg/L) | nano-IMI Scenario | PEC_nano-IMI_ (μg/L) | | |
| --- | --- | --- | --- | --- | --- |
|  |  |  | PEC_nano-IMI,free_ | PEC_nano-IMI,agg_ | PEC_nano-IMI,att_ |
| R1 | 12.64 | S1 α_1_ | 3.67E-09 | - | - |
|  |  | S1 α_2_ | 1.25E-09 | - | - |
|  |  | S1 α_3_ | 1.38E-09 | - | - |
|  |  | S2 α_1_ | 3.67E-09 | 1.57E-04 | 2.74E-07 |
|  |  | S2 α_2_ | 1.25E-09 | 1.57E-04 | 2.80E-07 |
|  |  | S2 α_3_ | 1.38E-09 | 1.57E-04 | 2.77E-07 |
| R2 | 131.03 | S1 α_1_ | 1.42E-07 | - | - |
|  |  | S1 α_2_ | 4.84E-08 | - | - |
|  |  | S1 α_3_ | 5.32E-08 | - | - |
|  |  | S2 α_1_ | 1.42E-07 | 6.01E-03 | 4.69E-07 |
|  |  | S2 α_2_ | 4.84E-08 | 6.01E-03 | 6.87E-07 |
|  |  | S2 α_3_ | 5.32E-08 | 6.01E-03 | 6.03E-07 |
| R3 | 162.84 | S1 α_1_ | 6.55E-07 | - | - |
|  |  | S1 α_2_ | 2.24E-07 | - | - |
|  |  | S1 α_3_ | 2.46E-07 | - | - |
|  |  | S2 α_1_ | 6.55E-07 | 2.72E-02 | 1.20E-06 |
|  |  | S2 α_2_ | 2.24E-07 | 2.72E-02 | 2.21E-06 |
|  |  | S2 α_3_ | 2.46E-07 | 2.72E-02 | 1.82E-06 |

Table S26. Ecotoxicity data of nano-IMI for effect factor (*EF*) derivative. The green background indicates the toxicity data obtained from previous experiments [37].

| **Species** | **Time (h)** | **Acute/Chronic** | **Test endpoint** | **L/EC_50_** | **Toxicity (mg/L)** |
| --- | --- | --- | --- | --- | --- |
| Crustaceans | | | | | |
| *Daphnia magna* | 48 | Acute | Mortality | LC_50_ | 28.9 |
| Fish | | | | | |
| *Danio rerio* | 120 | Acute | Mortality | LC_50_ | 191 |
| Insects | | | | | |
| *Chironomus kiiensis* | 96 | Acute | Mortality | LC_50_ | 3.35E-03 |
| *Chironomus kiiensis* | 240 | Acute | Mortality | LC_50_ | 3.75E-03 |
| *Chironomus kiiensis* | 552 | Acute | Mortality | LC_50_ | 1.55E-03 |
| *Chironomus kiiensis* | 240 | Acute | Midge growth | EC_50_ | 2.41E-03 |
| *Chironomus kiiensis* | 552 | Acute | Emergence | EC_50_ | 1.62E-03 |

Table S27. Ecotoxicity data of IMI for effect factor (*EF*_1_) derivative. Data were collected from the ECOtox database. The green background indicates the toxicity data obtained from previous experiments [37].

| **Species** | **Time (h)** | **Acute/Chronic** | **Test endpoint** | **L/EC_50_** | **Toxicity (mg/L)** | **Source** |
| --- | --- | --- | --- | --- | --- | --- |
| Crustaceans | | | | | | |
| *Daphnia magna* | 48 | Acute | Mortality | LC_50_ | 28.9 | [37] |
| *Daphnia magna* | 48 | Acute | Mortality | LC_50_ | 102 | [38] |
| *Daphnia magna* | 504 | Chronic | reproduction | EC_50_ | 2 | [39] |
| *Daphnia magna* | 504 | Chronic | reproduction | EC_50_ | 5.61 |  |
| *Daphnia magna* | 504 | Chronic | reproduction | EC_50_ | 5.8 |  |
| *Daphnia magna* | 504 | Chronic | reproduction | EC_50_ | 5.65 |  |
| *Daphnia magna* | 504 | Chronic | reproduction | EC_50_ | 7.75 |  |
| *Daphnia magna* | 504 | Chronic | reproduction | EC_50_ | 6.16 |  |
| *Daphnia magna* | 504 | Chronic | reproduction | EC_50_ | 6.3 |  |
| *Daphnia magna* | 48 | Acute | Immobility | LC_50_ | 10.4 | [40] |
| *Daphnia magna* | 48 | Acute | Immobility | LC_50_ | 17.4 |  |
| *Daphnia magna* | 48 | Acute | Immobilization | EC_50_ | 43.3 | [41] |
| *Daphnia magna* | 504 | Chronic | Reproduction | EC_50_ | 5.5 | [42] |
| *Daphnia magna* | 48 | Acute | Immobility | EC_50_ | 93.9 | [43] |
| *Daphnia magna* | 24 | Acute | Immobility | EC_50_ | 97.9 | [44] |
| *Daphnia magna* | 48 | Acute | Immobility | EC_50_ | 56.6 |  |
| *Daphnia magna* | 24 | Acute | Immobility | EC_50_ | 38 |  |
| *Daphnia magna* | 48 | Acute | Immobility | EC_50_ | 30 |  |
| *Daphnia magna* | 504 | Chronic | Survival | EC_50_ | 37.2 | [45] |
| *Daphnia magna* | 504 | Chronic | Survival | EC_50_ | 34.1 |  |
| *Daphnia magna* | 504 | Chronic | Survival | EC_50_ | 31.1 |  |
| *Daphnia magna* | 504 | Chronic | Survival | EC_50_ | 28.4 |  |
| *Daphnia magna* | 24 | Acute | Immobility | EC_50_ | 11.8 | [46] |
| *Daphnia magna* | 24 | Acute | Mortality | LC_50_ | 320 |  |
| *Daphnia magna* | 48 | Acute | Immobility | EC_50_ | 6.03 |  |
| *Daphnia magna* | 48 | Acute | Mortality | LC_50_ | 64.9 | [46] |
| *Daphnia magna* | 48 | Acute | Immobility | EC_50_ | 16.5 | [47] |
| *Daphnia magna* | 48 | Acute | Embryo hatching | EC_50_ | 16.2 |  |
| *Daphnia magna* | 48 | Acute | Immobility | EC_50_ | 85.2 | [48] |
| *Daphnia magna* | 48 | Acute | Mortality | EC_50_ | 96.7 | [49] |
| *Daphnia magna* | 48 | Acute | Mortality | EC_50_ | 90.7 |  |
| *Daphnia magna* | 504 | Chronic | Reproductive success | EC_50_ | 4.59 | [50] |
| *Daphnia magna* | 504 | Chronic | Mortality | LC_50_ | 34.4 |  |
| *Daphnia magna* | 24 | Acute | Immobility | EC_50_ | 3.7 | [51] |
| *Daphnia magna* | 48 | Acute | Mortality | LC_50_ | 97 |  |
| Fish | | | | | | |
| *Danio rerio* | 120 | Acute | Mortality | LC_50_ | 628 | [37] |
| *Danio rerio* | 96 | Acute | Mortality | LC_50_ | 277 | [52] |
| *Danio rerio* | 72 | Acute | Mortality | LC_50_ | 288 |  |
| *Danio rerio* | 48 | Acute | Mortality | LC_50_ | 305 |  |
| *Danio rerio* | 24 | Acute | Mortality | LC_50_ | 312 |  |
| *Danio rerio* | 48 | Acute | Missing eye pigmentation | EC_50_ | 732 | [44] |
| *Danio rerio* | 48 | Acute | Missing body pigmentation | EC_50_ | 626 |  |
| *Danio rerio* | 48 | Acute | Missing blood flow | EC_50_ | 408 |  |
| *Danio rerio* | 48 | Acute | Incomplete eye development | EC_50_ | 760 |  |
| *Danio rerio* | 48 | Acute | Incomplete ear development | EC_50_ | 626 |  |
| *Danio rerio* | 48 | Acute | Missing somites | LC_50_ | 826 |  |
| *Danio rerio* | 48 | Acute | Egg coagulation | LC_50_ | 1160 |  |
| *Danio rerio* | 48 | Acute | Missing heartbeat | LC_50_ | 502 |  |
| *Danio rerio* | 48 | Acute | Missing tail detachment | LC_50_ | 1150 |  |
| *Danio rerio* | 96 | Acute | Mortality | LC_50_ | 241 |  |
| *Danio rerio* | 96 | Acute | Mortality | LC_50_ | 214 |  |
| *Danio rerio* | 48 | Acute | Mortality | LC_50_ | 187 | [53] |
| *Danio rerio* | 96 | Acute | Mortality | LC_50_ | 144 |  |
| *Danio rerio* | 96 | Acute | Mortality | LC_50_ | 122 | [54] |
| *Danio rerio* | 96 | Acute | Mortality | LC_50_ | 129 |  |
| *Danio rerio* | 96 | Acute | Mortality | LC_50_ | 26.4 |  |
| *Danio rerio* | 96 | Acute | Mortality | LC_50_ | 76.1 |  |
| *Danio rerio* | 72 | Acute | Mortality | LC_50_ | 151 |  |
| *Danio rerio* | 48 | Acute | Mortality | LC_50_ | 352 |  |
| *Danio rerio* | 24 | Acute | Mortality | LC_50_ | 434 |  |
| Insects | | | | | | |
| *Chironomus kiiensis* | 96 | Acute | Mortality | LC_50_ | 2.63E-03 | [37] |
| *Chironomus kiiensis* | 240 | Acute | Mortality | LC_50_ | 7.40E-03 |  |
| *Chironomus kiiensis* | 552 | Acute | Mortality | LC_50_ | 1.52E-03 |  |
| *Chironomus kiiensis* | 240 | Acute | Midge growth | EC_50_ | 8.12E-03 |  |
| *Chironomus kiiensis* | 552 | Acute | Emergence | EC_50_ | 1.55E-03 |  |

Table S28. Additional toxicity data considered to derive the effect factor (*EF*_2_). Data were collected from the ECOtox database.

| **Species** | **Time (h)** | **Acute/**  **Chronic** | **Test endpoint** | **L/EC_50_** | **Toxicity (mg/L)** | **Source** |
| --- | --- | --- | --- | --- | --- | --- |
| Crustaceans | | | | | | |
| *Arthropoda* spp*.* | 2192 | Acute | Diversity, Evenness | EC_50_ | 2.08E-03 | [55] |
| *Arthropoda* spp. | 2192 | Acute | Abundance | EC_50_ | 1.06E-03 |  |
| *Arthropoda* spp. | 2192 | Acute | Diversity, Evenness | EC_50_ | 6.17E-03 |  |
| *Arthropoda* spp. | 2192 | Acute | Index to population size | EC_50_ | 1.14E-03 |  |
| *Asellus aquaticus* | 24 | Acute | Immobility | LC_50_ | 0.8 | [56] |
| *Asellus aquaticus* | 48 | Acute | Mortality | EC_50_ | 8.5 |  |
| *Asellus aquaticus* | 96 | Chronic | Immobility | LC_50_ | 0.119 | [57] |
| *Asellus aquaticus* | 96 | Chronic | Mortality | EC_50_ | 0.316 |  |
| *Asellus aquaticus* | 96 | Chronic | Immobility | LC_50_ | 0.078 |  |
| *Asellus aquaticus* | 96 | Chronic | Mortality | EC_50_ | 20 |  |
| *Asellus aquaticus* | 24 | Acute | Paralysis | LC_50_ | 0.8 | [58] |
| *Asellus aquaticus* | 48 | Acute | Mortality | EC_50_ | 8.5 |  |
| *Asellus aquaticus* | 48 | Acute | Mortality | LC_50_ | 0.603 | [59] |
| *Asellus aquaticus* | 48 | Acute | Immobility | LC_50_ | 1.52 |  |
| *Asellus aquaticus* | 96 | Chronic | Immobility | EC_50_ | 0.119 | [60] |
| *Asellus aquaticus* | 96 | Chronic | Mortality | EC_50_ | 0.316 |  |
| *Asellus aquaticus* | 672 | Chronic | Immobility | LC_50_ | 0.0119 |  |
| *Asellus aquaticus* | 672 | Chronic | Mortality | EC_50_ | 0.0203 |  |
| *Caecidotea acuticarpa* | 96 | Chronic | Immobility | LC_50_ | 0.321 | [38] |
| *Caecidotea acuticarpa* | 96 | Chronic | Mortality | EC_50_ | 15.6 |  |
| *Ceriodaphnia dubia* | 48 | Acute | Mortality | LC_50_ | 72.1 |  |
| *Ceriodaphnia dubia* | 48 | Acute | Immobilization | LC_50_ | 0.572 | [41] |
| *Ceriodaphnia dubia* | 48 | Acute | Mortality | EC_50_ | 2.07E-03 | [61] |
| *Ceriodaphnia dubia* | 144 | Chronic | Reproductive success | LC_50_ | 2.98 | [50] |
| *Ceriodaphnia dubia* | 144 | Chronic | Mortality | EC_50_ | 8.42 |  |
| *Ceriodaphnia reticulata* | 48 | Acute | Immobilization | LC_50_ | 5.55 | [41] |
| *Chydorus sphaericus* | 24 | Acute | Immobility | EC_50_ | 1.47 | [46] |
| *Chydorus sphaericus* | 24 | Acute | Immobility | EC_50_ | 18.7 |  |
| *Chydorus sphaericus* | 24 | Acute | Mortality | EC_50_ | 162 |  |
| *Chydorus sphaericus* | 48 | Acute | Immobility | LC_50_ | 0.832 |  |
| *Chydorus sphaericus* | 48 | Acute | Immobility | EC_50_ | 2.21 |  |
| *Chydorus sphaericus* | 48 | Acute | Mortality | EC_50_ | 133 |  |
| *Cypretta seurati* | 24 | Acute | Immobility | LC_50_ | 0.012 |  |
| *Cypretta seurati* | 24 | Acute | Immobility | EC_50_ | 0.046 |  |
| *Cypretta seurati* | 24 | Acute | Mortality | EC_50_ | 0.732 |  |
| *Cypretta seurati* | 48 | Acute | Immobility | LC_50_ | 0.016 |  |
| *Cypretta seurati* | 48 | Acute | Immobility | EC_50_ | 1.00E-03 |  |
| *Cypretta seurati* | 48 | Acute | Mortality | EC_50_ | 0.301 |  |
| *Cypridopsis vidua* | 24 | Acute | Immobility | LC_50_ | 8.00E-03 |  |
| *Cypridopsis vidua* | 24 | Acute | Immobility | EC_50_ | 0.016 |  |
| *Cypridopsis vidua* | 24 | Acute | Mortality | EC_50_ | 4 |  |
| *Cypridopsis vidua* | 24 | Acute | Mortality | LC_50_ | 0.542 |  |
| *Cypridopsis vidua* | 48 | Acute | Immobility | LC_50_ | 3.00E-03 |  |
| *Cypridopsis vidua* | 48 | Acute | Immobility | EC_50_ | 0.01 |  |
| *Cypridopsis vidua* | 48 | Acute | Mortality | EC_50_ | 0.715 |  |
| *Cypridopsis vidua* | 48 | Acute | Mortality | LC_50_ | 0.273 |  |
| *Daphnia pulex* | 48 | Acute | Immobilization | EC_50_ | 36.9 | [41] |
| *Daphnia pulex* | 24 | Acute | Mortality | LC_50_ | 1.50E-03 | [62] |
| *Daphnia pulex* | 48 | Acute | Mortality | LC_50_ | 1.09E-03 |  |
| *Diaptomus affinis* | 96 | Chronic | Immobility | EC_50_ | 3.86E-05 | [63] |
| *Diaptomus affinis* | 96 | Chronic | Mortality | LC_50_ | 6.54E-03 |  |
| *Eriocheir sinensis* | 96 | Chronic | Mortality | LC_50_ | 25 | [64] |
| *Gammarus fossarum* | 24 | Acute | Immobility | EC_50_ | 0.07 | [56] |
| *Gammarus fossarum* | 48 | Acute | Mortality | LC_50_ | 0.8 |  |
| *Gammarus fossarum* | 168 | Chronic | Food consumption | EC_50_ | 2.23E-03 | [65] |
| *Gammarus fossarum* | 168 | Chronic | Food consumption | EC_50_ | 8.26E-03 |  |
| *Gammarus fossarum* | 24 | Acute | Paralysis | EC_50_ | 0.07 | [58] |
| *Gammarus fossarum* | 48 | Acute | Mortality | LC_50_ | 0.8 |  |
| *Gammarus pulex* | 96 | Chronic | Immobility | EC_50_ | 0.018 | [57] |
| *Gammarus pulex* | 96 | Chronic | Mortality | LC_50_ | 0.263 |  |
| *Gammarus pulex* | 96 | Chronic | Immobility | EC_50_ | 0.049 |  |
| *Gammarus pulex* | 96 | Chronic | Mortality | LC_50_ | 0.386 |  |
| *Gammarus pulex* | 96 | Chronic | Immobility | LC_50_ | 0.131 | [66] |
| *Gammarus pulex* | 48 | Acute | Mortality | LC_50_ | 3.86 | [67] |
| *Gammarus pulex* | 24 | Acute | Mortality | LC_50_ | 8.76 |  |
| *Gammarus pulex* | 24 | Acute | Food consumption | EC_50_ | 19 | [68] |
| *Gammarus pulex* | 96 | Chronic | Food consumption | EC_50_ | 5.34 |  |
| *Gammarus pulex* | 72 | Acute | Food consumption | EC_50_ | 10.5 |  |
| *Gammarus pulex* | 48 | Acute | Food consumption | EC_50_ | 20.6 |  |
| *Gammarus pulex* | 96 | Chronic | Mortality | LC_50_ | 0.27 | [69] |
| *Gammarus pulex* | 96 | Chronic | Immobility | EC_50_ | 0.0183 | [60] |
| *Gammarus pulex* | 96 | Chronic | Mortality | LC_50_ | 0.263 |  |
| *Gammarus pulex* | 672 | Chronic | Immobility | EC_50_ | 0.0154 |  |
| *Gammarus pulex* | 672 | Chronic | Mortality | LC_50_ | 0.0338 |  |
| *Gammarus roeseli* | 96 | Chronic | Immobility | EC_50_ | 1.90E-03 | [70] |
| *Gammarus roeseli* | 96 | Chronic | Immobility | EC_50_ | 0.0142 |  |
| *Hyalella azteca* | 96 | Chronic | Mortality | LC_50_ | 0.0654 | [71] |
| *Hyalella azteca* | 96 | Chronic | Mortality | LC_50_ | 0.0174 |  |
| *Hyalella azteca* | 672 | Chronic | Mortality | LC_50_ | 7.08E-03 |  |
| *Hyalella azteca* | 240 | Chronic | Mortality | LC_50_ | 7.01E-03 |  |
| *Hyalella azteca* | 672 | Chronic | Mortality | LC_50_ | 9.74E-03 |  |
| *Hyalella azteca* | 240 | Chronic | Dry weight | EC_50_ | 0.0103 |  |
| *Hyalella azteca* | 240 | Chronic | Dry weight | EC_50_ | 9.83E-03 |  |
| *Hyalella azteca* | 672 | Chronic | Weight | EC_50_ | 4.30E-03 | [72] |
| *Hyalella azteca* | 672 | Chronic | Mortality | LC_50_ | 0.09 |  |
| *Hyalella azteca* | 168 | Chronic | Mortality | LC_50_ | 0.23 |  |
| *Hyalella azteca* | 336 | Chronic | Mortality | LC_50_ | 0.13 |  |
| *Hyalella azteca* | 504 | Chronic | Mortality | LC_50_ | 0.1 |  |
| *Hyalella azteca* | 96 | Chronic | Lethality | LC_50_ | 0.363 | [73] |
| *Hyalella azteca* | 96 | Chronic | Immobility | EC_50_ | 0.177 |  |
| *Hyalella azteca* | 96 | Chronic | Immobility | EC_50_ | 0.177 | [38] |
| *Hyalella azteca* | 96 | Chronic | Mortality | LC_50_ | 0.363 |  |
| *Hyalella azteca* | 96 | Chronic | Multiple effects | EC_50_ | 0.0335 | [74] |
| *Hyalella azteca* | 48 | Acute | Mortality | LC_50_ | 0.115 | [48] |
| *Hyalella azteca* | 96 | Chronic | Mortality | LC_50_ | 0.055 |  |
| *Ilyocypris dentifera* | 24 | Acute | Immobility | EC_50_ | 5.00E-03 | [46] |
| *Ilyocypris dentifera* | 24 | Acute | Immobility | EC_50_ | 0.013 |  |
| *Ilyocypris dentifera* | 24 | Acute | Mortality | LC_50_ | 1.12 |  |
| *Ilyocypris dentifera* | 24 | Acute | Mortality | LC_50_ | 0.759 |  |
| *Ilyocypris dentifera* | 48 | Acute | Immobility | EC_50_ | 3.00E-03 | [46] |
| *Ilyocypris dentifera* | 48 | Acute | Immobility | EC_50_ | 3.00E-03 |  |
| *Ilyocypris dentifera* | 48 | Acute | Mortality | LC_50_ | 0.214 |  |
| *Ilyocypris dentifera* | 48 | Acute | Mortality | LC_50_ | 0.517 |  |
| *Moina macrocopa* | 48 | Acute | Immobilization | EC_50_ | 45.3 | [41] |
| *Zooplankton* spp. | 2192 | Chronic | Diversity, Evenness | EC_50_ | 1.28E-03 | [55] |
| *Zooplankton* spp. | 2192 | Chronic | Abundance | EC_50_ | 3.60E-04 |  |
| *Zooplankton* spp. | 2192 | Chronic | Diversity, Evenness | EC_50_ | 1.67E-03 |  |
| *Zooplankton* spp. | 2192 | Chronic | Index to population size | EC_50_ | 4.40E-04 |  |
| Fish | | | | | | |
| *Acipenser transmontanus* | 96 | Acute | Mortality | LC_50_ | 124 | [75] |
| *Acipenser transmontanus* | 96 | Acute | Mortality | LC_50_ | 124 | [76] |
| *Carassius auratus* | 96 | Acute | Mortality | LC_50_ | 24.8 | [77] |
| *Carassius auratus* | 96 | Acute | Mortality | LC_50_ | 6.2 |  |
| *Clarias gariepinus* | 96 | Acute | Mortality | LC_50_ | 0.0102 | [78] |
| *Ctenopharyngodon idella* | 96 | Acute | Mortality | LC_50_ | 3.3 | [77] |
| *Ctenopharyngodon idella* | 96 | Acute | Mortality | LC_50_ | 13.2 |  |
| *Cyprinus carpio* | 96 | Acute | Mortality | LC_50_ | 1.29 | [79] |
| *Cyprinus carpio* | 72 | Acute | Mortality | LC_50_ | 0.275 |  |
| *Cyprinus carpio* | 96 | Acute | Mortality | LC_50_ | 6.68 | [77] |
| *Cyprinus carpio* | 96 | Acute | Mortality | LC_50_ | 1.67 |  |
| *Labeo rohita* | 96 | Acute | Mortality | LC_50_ | 550 | [80] |
| *Lepomis macrochirus* | 96 | Acute | Mortality | LC_50_ | 105 | [48] |
| *Misgurnus anguillicaudatus* | 24 | Acute | Mortality | LC_50_ | 168 | [81] |
| *Misgurnus anguillicaudatus* | 48 | Acute | Mortality | LC_50_ | 159 |  |
| *Misgurnus anguillicaudatus* | 72 | Acute | Mortality | LC_50_ | 148 |  |
| *Misgurnus anguillicaudatus* | 96 | Acute | Mortality | LC_50_ | 146 |  |
| *Oncorhynchus mykiss* | 96 | Acute | Mortality | LC_50_ | 83 | [48] |
| *Oncorhynchus mykiss* | 96 | Acute | Mortality | LC_50_ | 229 |  |
| *Oreochromis niloticus* | 24 | Acute | Mortality | LC_50_ | 13.4 | [82] |
| *Oreochromis niloticus* | 96 | Acute | Mortality | LC_50_ | 1.09E-04 | [83] |
| *Pimephales promelas* | 96 | Acute | Multiple effects | EC_50_ | 1 | [74] |
| Algae | | | | | | |
| *Desmodesmus subspicatus* | 72 | Chronic | Growth inhibitions | IC_50_ | 389 | [44] |
| *Desmodesmus subspicatus* | 72 | Chronic | Growth inhibitions | IC_50_ | 116 |  |
| Amphibian | | | | | | |
| *Hypsiboas pulchellus* | 24 | Acute | Mortality | LC_50_ | 131 | [84] |
| *Hypsiboas pulchellus* | 48 | Acute | Mortality | LC_50_ | 92.6 |  |
| *Hypsiboas pulchellus* | 72 | Acute | Mortality | LC_50_ | 92.6 |  |
| *Hypsiboas pulchellus* | 96 | Acute | Mortality | LC_50_ | 84.9 |  |
| *Hypsiboas pulchellus* | 96 | Acute | Mortality | LC_50_ | 52.6 | [85] |
| *Hypsiboas pulchellus* | 24 | Acute | Mortality | LC_50_ | 69.4 |  |
| *Hypsiboas pulchellus* | 48 | Acute | Mortality | LC_50_ | 58.2 |  |
| *Hypsiboas pulchellus* | 72 | Acute | Mortality | LC_50_ | 56.8 |  |
| *Rana limnocharis* | 96 | Acute | Mortality | LC_50_ | 82 | [86] |
| *Rana nigronaculata Hallowell* | 96 | Acute | Mortality | LC_50_ | 129 |  |
| *Xenopus tropicalis* | 96 | Acute | Mortality | LC_50_ | 100 | [87] |
| Mollusc | | | | | | |
| *Planorbella pilsbryi* | 672 | Chronic | Weight | EC_50_ | 0.042 | [88] |
| *Planorbella pilsbryi* | 672 | Chronic | Biomass | EC_50_ | 0.0394 |  |
| *Planorbella pilsbryi* | 168 | Acute | Mortality | LC_50_ | 3.98 |  |
| *Planorbella pilsbryi* | 672 | Chronic | Mortality | LC_50_ | 0.646 |  |
| *Lampsilis fasciola* | 48 | Acute | Mortality | LC_50_ | 0.688 |  |
| Worm | | | | | | |
| *Lumbriculus variegatus* | 96 | Acute | Lethality | EC_50_ | 0.0454 | [73] |
| *Lumbriculus variegatus* | 96 | Acute | Immobility | LC_50_ | 0.0324 |  |
| *Lumbriculus variegatus* | 96 | Acute | Immobility | EC_50_ | 0.0324 | [38] |
| *Lumbriculus variegatus* | 96 | Acute | Mortality | EC_50_ | 0.0454 |  |
| *Lumbriculus variegatus* | 96 | Acute | Immobility | LC_50_ | 6.20E-03 | [89] |
| *Lumbriculus variegatus* | 24 | Acute | Mortality | EC_50_ | 0.0653 | [90] |
| *Lumbriculus variegatus* | 24 | Acute | Mortality | LC_50_ | 0.0878 |  |
| *Tubifex* | 48 | Acute | Mortality | LC_50_ | 1.66 | [91] |
| *Tubifex* | 6 | Acute | Movements | LC_50_ | 0.14 | [92] |
| *Tubifex* | 12 | Acute | Movements | EC_50_ | 0.11 |  |
| *Tubifex* | 18 | Acute | Movements | EC_50_ | 0.09 |  |
| *Tubifex* | 24 | Acute | Movements | EC_50_ | 0.09 |  |
| *Tubifex* | 24 | Acute | Mortality | EC_50_ | 0.32 |  |

Table S29. Additional toxicity data considered to derive the effect factor (*EF*_3_). Data were collected from the ECOtox database.

| **Species** | **Time (h)** | **Acute/**  **Chronic** | **Test endpoint** | **L/EC_50_** | **Toxicity (mg/L)** | **Source** |
| --- | --- | --- | --- | --- | --- | --- |
| Insects | | | | | | |
| *Aedes aegypti* | 24 | Acute | Mortality | LC_50_ | 0.037 | [93] |
| *Aedes aegypti* | 72 | Acute | Mortality | LC_50_ | 0.21 | [94] |
| *Aedes aegypti* | 48 | Acute | Mortality | LC_50_ | 0.044 | [40] |
| *Aedes aegypti* | 48 | Acute | Mortality | LC_50_ | 0.045 |  |
| *Aedes aegypti* | 24 | Acute | Mortality | LC_50_ | 0.15 | [95] |
| *Aedes aegypti* | 72 | Acute | Mortality | LC_50_ | 0.084 | [96] |
| *Aedes aegypti* | 24 | Acute | Mortality | LC_50_ | 0.82 | [97] |
| *Aedes aegypti* | 24 | Acute | Mortality | LC_50_ | 1.83 | [98] |
| *Aedes aegypti* | 24 | Acute | Mortality | LC_50_ | 8.35 |  |
| *Aedes aegypti* | 24 | Acute | Mortality | LC_50_ | 3.18 | [99] |
| *Aedes aegypti* | 48 | Acute | Mortality | LC_50_ | 0.36 |  |
| *Aedes aegypti* | 72 | Acute | Mortality | LC_50_ | 0.139 |  |
| *Aedes albopictus* | 48 | Acute | Mortality | LC_50_ | 0.0408 | [38] |
| *Aedes albopictus* | 24 | Acute | Mortality | LC_50_ | 0.6 | [100] |
| *Aedes albopictus* | 24 | Acute | Mortality | LC_50_ | 0.3 |  |
| *Aedes albopictus* | 24 | Acute | Mortality | LC_50_ | 0.8 |  |
| *Aedes albopictus* | 24 | Acute | Mortality | LC_50_ | 0.6 |  |
| *Aedes albopictus* | 24 | Acute | Mortality | LC_50_ | 0.5 |  |
| *Anopheles stephensi* | 72 | Acute | Mortality | LC_50_ | 0.066 | [94] |
| *Anopheles stephensi* | 72 | Acute | Mortality | LC_50_ | 0.049 |  |
| *Baetis rhodani* | 96 | Acute | Mortality | LC_50_ | 8.49E-03 | [69] |
| *Caenis horaria* | 96 | Acute | Immobility | EC_50_ | 1.80E-03 | [57] |
| *Caenis horaria* | 96 | Acute | Mortality | LC_50_ | 6.70E-03 |  |
| *Caenis horaria* | 96 | Acute | Immobility | EC_50_ | 6.00E-03 |  |
| *Caenis horaria* | 96 | Acute | Mortality | LC_50_ | 0.028 |  |
| *Caenis horaria* | 96 | Acute | Immobility | EC_50_ | 0.0218 | [38] |
| *Caenis horaria* | 96 | Acute | Mortality | LC_50_ | 0.0218 |  |
| *Caenis horaria* | 96 | Acute | Immobility | EC_50_ | 1.77E-03 | [60] |
| *Caenis horaria* | 96 | Acute | Mortality | LC_50_ | 6.68E-03 |  |
| *Caenis horaria* | 672 | Acute | Immobility | EC_50_ | 1.26E-04 |  |
| *Caenis horaria* | 672 | Acute | Mortality | LC_50_ | 3.16E-04 |  |
| *Chaetopteryx villosa* | 168 | Acute | Food consumption | EC_50_ | 7.05E-03 | [65] |
| *Chaetopteryx villosa* | 168 | Acute | Food consumption | EC_50_ | 0.0194 |  |
| *Chaetopteryx villosa* | 168 | Acute | Mortality | LC_50_ | 0.0114 |  |
| *Chaoborus obscuripes* | 96 | Acute | Immobility | EC_50_ | 0.284 | [57] |
| *Chaoborus obscuripes* | 96 | Acute | Mortality | LC_50_ | 0.294 |  |
| *Chaoborus obscuripes* | 96 | Acute | Immobility | EC_50_ | 3.26 |  |
| *Chaoborus obscuripes* | 96 | Acute | Mortality | LC_50_ | 5.23 |  |
| *Chaoborus obscuripes* | 96 | Acute | Immobility | EC_50_ | 0.284 | [60] |
| *Chaoborus obscuripes* | 96 | Acute | Mortality | LC_50_ | 0.294 |  |
| *Chaoborus obscuripes* | 672 | Acute | Immobility | EC_50_ | 0.0118 |  |
| *Chaoborus obscuripes* | 672 | Acute | Mortality | LC_50_ | 0.0126 |  |
| *Cheumatopsyche brevilineata* | 48 | Acute | Immobility | EC_50_ | 4.22E-03 | [101] |
| *Cheumatopsyche brevilineata* | 48 | Acute | Immobility | EC_50_ | 4.85E-03 |  |
| *Cheumatopsyche brevilineata* | 48 | Acute | Immobility | EC_50_ | 5.24E-03 |  |
| *Cheumatopsyche brevilineata* | 96 | Acute | Immobility | EC_50_ | 0.176 | [38] |
| *Cheumatopsyche brevilineata* | 96 | Acute | Mortality | LC_50_ | 0.324 |  |
| *Chironomus dilutus* | 96 | Acute | Lethality | LC_50_ | 0.0118 | [73] |
| *Chironomus dilutus* | 96 | Acute | Immobility | EC_50_ | 2.47E-03 |  |
| *Chironomus dilutus* | 96 | Acute | Immobility | EC_50_ | 2.50E-03 | [38] |
| *Chironomus dilutus* | 96 | Acute | Mortality | LC_50_ | 0.0118 |  |
| *Chironomus dilutus* | 672 | Acute | Emergence | EC_50_ | 5.00E-04 | [102] |
| *Chironomus dilutus* | 336 | Acute | Weight | EC_50_ | 7.20E-04 | [103] |
| *Chironomus dilutus* | 1344 | Acute | First emergence | EC_50_ | 1.43E-03 |  |
| *Chironomus dilutus* | 1344 | Acute | Germ cell count | EC_50_ | 7.20E-04 |  |
| *Chironomus dilutus* | 1344 | Acute | Emergence | EC_50_ | 2.40E-04 |  |
| *Chironomus dilutus* | 336 | Acute | Mortality | LC_50_ | 1.43E-03 |  |
| *Chironomus dilutus* | 1344 | Acute | Lifespan | LC_50_ | 9.00E-04 |  |
| *Chironomus dilutus* | 960 | Acute | Sex ratio | EC_50_ | 1.70E-04 | [104] |
| *Chironomus dilutus* | 336 | Acute | Biomass | EC_50_ | 2.23E-03 |  |
| *Chironomus dilutus* | 960 | Acute | Emergence | EC_50_ | 3.90E-04 |  |
| *Chironomus dilutus* | 336 | Acute | Mortality | LC_50_ | 1.52E-03 |  |
| *Chironomus dilutus* | 96 | Acute | Mortality | LC_50_ | 7.00E-03 | [105] |
| *Chironomus dilutus* | 96 | Acute | Mortality | LC_50_ | 4.63E-03 | [106] |
| *Chironomus dilutus* | 96 | Acute | Mortality | LC_50_ | 2.65E-03 | [107] |
| *Chironomus dilutus* | 96 | Acute | Burrowing length | EC_50_ | 6.80E-04 | [108] |
| *Chironomus dilutus* | 96 | Acute | Mortality | LC_50_ | 3.56E-03 |  |
| *Chironomus riparius* | 240 | Acute | Length | EC_50_ | 5.03E-03 | [109] |
| *Chironomus riparius* | 240 | Acute | Mortality | LC_50_ | 2.33E-03 |  |
| *Chironomus riparius* | 24 | Acute | Mortality | LC_50_ | 0.0315 |  |
| *Chironomus riparius* | 48 | Acute | Mortality | LC_50_ | 0.0199 | [110] |
| *Chironomus riparius* | 672 | Acute | Emergence | EC_50_ | 1.52 | [111] |
| *Chironomus riparius* | 672 | Acute | Emergence | EC_50_ | 2.63 |  |
| *Chironomus riparius* | 672 | Acute | Emergence | EC_50_ | 2.64 |  |
| *Chironomus riparius* | 672 | Acute | Emergence | EC_50_ | 2.75 |  |
| *Chironomus riparius* | 672 | Acute | Emergence | EC_50_ | 2.64 |  |
| *Chironomus tentans* | 96 | Acute | Mortality | LC_50_ | 5.75E-03 | [71] |
| *Chironomus tentans* | 96 | Acute | Mortality | LC_50_ | 5.40E-03 |  |
| *Chironomus tentans* | 672 | Acute | Emergence | EC_50_ | 9.10E-04 |  |
| *Chironomus tentans* | 240 | Acute | Dry weight | EC_50_ | 3.14E-03 |  |
| *Chironomus tentans* | 48 | Acute | Mortality | LC_50_ | 0.0689 | [48] |
| *Chironomus tepperi* | 24 | Acute | Mortality | LC_50_ | 3.04E-03 | [112] |
| *Chironomus tepperi* | 24 | Acute | Mortality | LC_50_ | 1.60E-03 |  |
| *Chironomus tepperi* | 24 | Acute | Mortality | LC_50_ | 2.90E-03 | [112] |
| *Chironomus tepperi* | 24 | Acute | Mortality | LC_50_ | 2.56E-03 |  |
| *Cloeon dipterum* | 96 | Acute | Immobility | EC_50_ | 0.025 | [57] |
| *Cloeon dipterum* | 96 | Acute | Mortality | LC_50_ | 0.034 |  |
| *Cloeon dipterum* | 96 | Acute | Immobility | EC_50_ | 1.00E-03 |  |
| *Cloeon dipterum* | 96 | Acute | Mortality | LC_50_ | 0.026 |  |
| *Cloeon dipterum* | 96 | Acute | Immobility | EC_50_ | 0.031 |  |
| *Cloeon dipterum* | 96 | Acute | Mortality | LC_50_ | 0.154 |  |
| *Cloeon dipterum* | 96 | Acute | Immobility | EC_50_ | 0.023 |  |
| *Cloeon dipterum* | 96 | Acute | Mortality | LC_50_ | 0.077 |  |
| *Cloeon dipterum* | 96 | Acute | Immobility | EC_50_ | 0.018 |  |
| *Cloeon dipterum* | 96 | Acute | Mortality | LC_50_ | 0.037 |  |
| *Cloeon dipterum* | 96 | Acute | Immobility | EC_50_ | 0.018 |  |
| *Cloeon dipterum* | 96 | Acute | Mortality | LC_50_ | 0.029 |  |
| *Cloeon dipterum* | 672 | Acute | Immobility | EC_50_ | 6.80E-04 |  |
| *Cloeon dipterum* | 672 | Acute | Mortality | LC_50_ | 8.50E-04 |  |
| *Cloeon dipterum* | 672 | Acute | Immobility | EC_50_ | 1.30E-04 |  |
| *Cloeon dipterum* | 672 | Acute | Mortality | LC_50_ | 3.20E-04 |  |
| *Cloeon dipterum* | 96 | Acute | Immobility | EC_50_ | 0.0231 | [38] |
| *Cloeon dipterum* | 96 | Acute | Mortality | LC_50_ | 1.15 |  |
| *Cloeon dipterum* | 96 | Acute | Immobility | EC_50_ | 5.48E-06 | [63] |
| *Cloeon dipterum* | 96 | Acute | Mortality | LC_50_ | 2.38E-05 |  |
| *Cloeon dipterum* | 96 | Acute | Immobility | EC_50_ | 1.02E-03 | [60] |
| *Cloeon dipterum* | 96 | Acute | Mortality | LC_50_ | 0.0263 |  |
| *Cloeon dipterum* | 672 | Acute | Immobility | EC_50_ | 1.23E-04 |  |
| *Cloeon dipterum* | 672 | Acute | Mortality | LC_50_ | 1.95E-04 |  |
| *Coenagrion aculeatum* | 96 | Acute | Immobility | EC_50_ | 5.44 | [38] |
| *Coenagrion aculeatum* | 96 | Acute | Mortality | LC_50_ | 3.46 |  |
| *Coloburiscus humeralis* | 96 | Acute | Immobile | EC_50_ | 0.0123 | [113] |
| *Coloburiscus humeralis* | 96 | Acute | Mortality | LC_50_ | 0.0317 |  |
| *Culex pipiens* | 72 | Acute | Mortality | LC_50_ | 8.10E-05 | [114] |
| *Culex pipiens* | 72 | Acute | Mortality | LC_50_ | 6.00E-05 |  |
| *Culex pipiens* | 24 | Acute | Mortality | LC_50_ | 0.555 | [115] |
| *Culex pipiens* | 24 | Acute | Mortality | LC_50_ | 1.02 |  |
| *Culex pipiens* | 48 | Acute | Mortality | LC_50_ | 0.323 |  |
| *Culex pipiens* | 48 | Acute | Mortality | LC_50_ | 0.705 |  |
| *Culex pipiens* | 72 | Acute | Mortality | LC_50_ | 0.167 |  |
| *Culex pipiens* | 72 | Acute | Mortality | LC_50_ | 0.621 |  |
| *Culex pipiens* | 24 | Acute | Mortality | LC_50_ | 0.117 | [116] |
| *Culex quinquefasciatus* | 72 | Acute | Mortality | LC_50_ | 0.02 | [94] |
| *Culex quinquefasciatus* | 24 | Acute | Mortality | LC_50_ | 0.4 | [117] |
| *Culex quinquefasciatus* | 24 | Acute | Mortality | LC_50_ | 0.3 |  |
| *Culex quinquefasciatus* | 24 | Acute | Mortality | LC_50_ | 0.04 |  |
| *Culex quinquefasciatus* | 24 | Acute | Mortality | LC_50_ | 0.2 | [100] |
| *Culex quinquefasciatus* | 24 | Acute | Mortality | LC_50_ | 0.027 | [118] |
| *Culex quinquefasciatus* | 48 | Acute | Mortality | LC_50_ | 0.022 |  |
| *Culex quinquefasciatus* | 72 | Acute | Mortality | LC_50_ | 0.021 |  |
| *Deleatidium vernale* | 672 | Acute | Righting response | EC_50_ | 1.90E-04 | [119] |
| *Deleatidium vernale* | 672 | Acute | Immobility | IC_50_ | 2.60E-04 |  |
| *Deleatidium vernale* | 672 | Acute | Mortality | LC_50_ | 2.80E-04 |  |
| *Deleatidium vernale* | 96 | Acute | Immobile | EC_50_ | 8.19E-03 | [113] |
| *Deleatidium vernale* | 96 | Acute | Mortality | LC_50_ | 0.0406 |  |
| *Epeorus longimanus* | 24 | Acute | Mortality | LC_50_ | 2.10E-03 | [89] |
| *Epeorus longimanus* | 96 | Acute | Mortality | LC_50_ | 6.50E-04 |  |
| *Ephemerella alleni* | 96 | Acute | Immobility | EC_50_ | 0.0106 | [38] |
| *Ephemerella alleni* | 96 | Acute | Mortality | LC_50_ | 0.0682 |  |
| *Gyrinus aquiris* | 96 | Acute | Immobility | EC_50_ | 0.0575 |  |
| *Gyrinus aquiris* | 96 | Acute | Mortality | LC_50_ | 0.132 |  |
| *Heptagenia adaequata* | 96 | Acute | Burrowing length | EC_50_ | 0.01 | [120] |
| *Heptagenia adaequata* | 96 | Acute | Mortality | LC_50_ | 0.9 |  |
| *Heptagenia adaequata* | 96 | Acute | Mortality | LC_50_ | 9.32 | [38] |
| *Heptageniidae* | 96 | Acute | Mortality | LC_50_ | 3.70E-03 | [121] |
| *Isonychia bicolor* | 96 | Acute | Immobility | EC_50_ | 0.0604 | [38] |
| *Isonychia bicolor* | 96 | Acute | Mortality | LC_50_ | 0.715 |  |
| *Isonychia bicolor* | 96 | Acute | Immobility | EC_50_ | 5.88E-03 | [122] |
| *Isonychia bicolor* | 96 | Acute | Mortality | LC_50_ | 0.0188 |  |
| *Limnephilidae* | 96 | Acute | Immobility | EC_50_ | 1.79E-03 | [60] |
| *Limnephilidae* | 96 | Acute | Mortality | LC_50_ | 0.0257 |  |
| *Maccaffertium appaloosa* | 96 | Acute | Immobility | EC_50_ | 0.0106 | [38] |
| *Maccaffertium appaloosa* | 96 | Acute | Mortality | LC_50_ | 1.81 |  |
| *Micrasema abbreviatum* | 96 | Acute | Immobility | EC_50_ | 6.40E-03 |  |
| *Micrasema abbreviatum* | 96 | Acute | Mortality | LC_50_ | 0.0146 |  |
| *Micrasema abbreviatum* | 96 | Acute | Immobility | EC_50_ | 0.0108 | [60] |
| *Micrasema abbreviatum* | 96 | Acute | Mortality | LC_50_ | 0.0282 |  |
| *Neocloeon triangulifer* | 96 | Acute | Lethality | LC_50_ | 5.21E-03 | [73] |
| *Neocloeon triangulifer* | 96 | Acute | Immobility | EC_50_ | 3.11E-03 |  |
| *Neocloeon triangulifer* | 96 | Acute | Immobility | EC_50_ | 3.10E-03 | [38] |
| *Neocloeon triangulifer* | 96 | Acute | Mortality | LC_50_ | 5.20E-03 |  |
| *Neocloeon triangulifer* | 768 | Acute | First emergence | EC_50_ | 2.11E-03 | [103] |
| *Neocloeon triangulifer* | 768 | Acute | Mortality | LC_50_ | 1.75E-03 |  |
| *Notonecta glauca* | 96 | Acute | Immobility | EC_50_ | 0.0182 | [60] |
| *Notonecta glauca* | 96 | Acute | Mortality | LC_50_ | 10 |  |
| *Plea minutissima* | 96 | Acute | Immobility | EC_50_ | 0.036 | [57] |
| *Plea minutissima* | 96 | Acute | Mortality | LC_50_ | 0.037 |  |
| *Plea minutissima* | 96 | Acute | Immobility | EC_50_ | 0.189 |  |
| *Plea minutissima* | 96 | Acute | Mortality | LC_50_ | 0.287 |  |
| *Plea minutissima* | 96 | Acute | Immobility | EC_50_ | 0.0359 | [60] |
| *Plea minutissima* | 96 | Acute | Mortality | LC_50_ | 0.0375 |  |
| *Plea minutissima* | 672 | Acute | Immobility | EC_50_ | 6.45E-03 |  |
| *Plea minutissima* | 672 | Acute | Mortality | LC_50_ | 9.80E-03 |  |
| *Pteronarcys dorsata* | 336 | Acute | Mortality | LC_50_ | 0.0701 | [123] |
| *Sialis lutaria* | 96 | Acute | Immobility | EC_50_ | 0.0506 | [60] |
| *Sialis lutaria* | 96 | Acute | Mortality | LC_50_ | 10 |  |
| *Sialis lutaria* | 672 | Acute | Immobility | EC_50_ | 3.46E-03 |  |
| *Sialis lutaria* | 672 | Acute | Mortality | LC_50_ | 0.0325 |  |
| *Simulium latigonium* | 96 | Acute | Mortality | LC_50_ | 3.73E-03 | [69] |
| *Simulium vittatum* | 48 | Acute | Mortality | LC_50_ | 6.75E-03 | [124] |
| *Simulium vittatum* | 48 | Acute | Mortality | LC_50_ | 9.54E-03 |  |
| *Simulium vittatum* | 48 | Acute | Mortality | LC_50_ | 8.25E-03 |  |
| *Stenelmis sexlineata* | 96 | Acute | Immobility | EC_50_ | 0.0992 | [38] |
| *Stenelmis sexlineata* | 96 | Acute | Mortality | LC_50_ | 0.366 |  |
| *Tipula paludosa* | 336 | Acute | Mortality | LC_50_ | 0.139 | [123] |
| *Trichocorixa arizonensis* | 48 | Acute | Immobility | EC_50_ | 0.0631 | [38] |
| *Trichocorixa arizonensis* | 48 | Acute | Mortality | LC_50_ | 0.45 |  |

Table S30. Summarized effects factors (*EF*) for IMI and nano-IMI.

| Scenario | | *EF* (PAF·m^3^·kg^-1^) | Species | Trophic level | Data point | Comment |
| --- | --- | --- | --- | --- | --- | --- |
| IMI | *EF* | 59.41 | 8 | 2 | - | USEtox |
|  | *EF*_1_ | 2641 | 3 | 2 | 65 | Uses the same species as nano-IMI |
|  | *EF*_2_ | 9007 | 40 | 3 | 221 | Non-target species (without insects) |
|  | *EF*_3_ | 74615 | 78 | 3 | 413 | All species |
| nano-IMI | - | 372.52 | 3 | 2 | 7 | Experimental data |

Table S31. The freshwater fate factor (*FF*) of IMI and nano-IMI.

| *FF*_IMI_ (d) | nano-IMI Scenario | *FF*_nano-IMI_ (d) |
| --- | --- | --- |
| 183.7 | S1 α_1_ | 0.057 |
|  | S1 α_2_ | 0.020 |
|  | S1 α_3_ | 0.021 |
|  | S2 α_1_ | 1.80 |
|  | S2 α_2_ | 1.76 |
|  | S2 α_3_ | 1.76 |

Table S32. Inputs and output for IMI’s exposure factors (*X*F) derivative [125].

| Equation | $\text{XF}\text{ = }\frac{\text{1}}{\text{1+}\text{K}_{\text{sus,x}}\text{∙}\frac{\text{ }\text{C}_{\text{sus}}}{\text{1000}}\text{+}\text{K}_{\text{doc,x}}\text{∙}\frac{\text{ }\text{C}_{\text{doc}}}{\text{1000}}\text{+}\text{BAF}_{\text{fish,x}}\text{∙}\frac{\text{ }\text{C}_{\text{biota}}}{\text{1000}}}$ | | |
| --- | --- | --- | --- |
| Parameter | Value | Unit | Reference |
| C_sus_ | 1.50E-02 | kg/m^3^ | USEtox document |
| C_doc_ | 5.00E-03 | kg/m^3^ |  |
| C_biota_ | 1.00E-03 | kg/m^3^ |  |
| K_sus, IMI_ | 1.58E+01 | L/kg | [126] |
| K_OW_ | 3.72E+00 | L/kg |  |
| K_doc_ | 2.97E-01 | L/kg | K_doc_ = 0.08* K_OW_ [127] |
| BAF_fish, IMI_ | 1.03E+00 | L/kg | [128] |
| *XF*_IMI_ | 0.99976 | - | Calculated in this study |
| *XF*_IMI_ | 0.99997 | - | USEtox input database |

Table S33. Inputs and output for nano-IMI’s exposure factors (*XF*) derivative based on different perspective on hetero-aggregation of nano-IMI in fresh water.

| Equation | $\text{XF}_{\text{S1}}\text{ = }\frac{\text{1}}{\text{1+}\mathrm{BCF}_{\mathrm{free}}\text{*[Biota]}}$  $\text{XF}_{\text{S2}}\text{ = }\frac{\text{1}}{\text{1+}\mathrm{BCF}_{free+agg+att}\text{*[Biota]}}$ | | |
| --- | --- | --- | --- |
| Parameter | Value | Unit | Reference |
| [biota] | 1.00E-03 | kg/m3 | USEtox 2.12 |
| BCF_free_ (IMI) | 0.33 | mL/g | Experimental data |
| BCF_free+agg+att_ (nano-IMI) | 0.48 | mL/g | Experimental data |
| *XF*_S1_ | 0.99999967 | | |
| *XF*_S2_ | 0.99999952 | | |

Table S34. The characterization factors (*CF*s) of IMI considering four different *EF*s.

|  | *EF* | *XF* | *FF* | *CF* |
| --- | --- | --- | --- | --- |
| Scenario | PAF·m^3^·kg^-1^ | - | d | PAF·m^3^·kg^-1^·d |
| *EF* | 59.41 | 0.99997  (USEtox default  IMI XF) | 183.7 | 1.09E+04 |
| *EF*_1_ | 2641 |  |  | 4.85E+05 |
| *EF*_2_ | 9007 |  |  | 1.65E+06 |
| *EF*_3_ | 74615 |  |  | 1.37E+07 |
| *EF* | 59.41 | 0.99976  (Calculated) |  | 1.09E+04 |
| *EF*_1_ | 2641 |  |  | 4.85E+05 |
| *EF*_2_ | 9007 |  |  | 1.65E+06 |
| *EF*_3_ | 74615 |  |  | 1.37E+07 |

Table S35. The characterization factors (*CF*s) of nano-IMI based on two hetero-aggregation scenarios and three attachment coefficients.

| Scenario | | *EF* | *XF* | *FF* | *CF* |
| --- | --- | --- | --- | --- | --- |
|  |  | PAF·m^3^·kg^-1^ | - | d | PAF·m^3^·kg^-1^·d |
| S1 | α_1_ | 372.5 | 0.99999967 | 0.0571 | 21.3 |
|  | α_2_ |  |  | 0.0195 | 7.28 |
|  | α_3_ |  |  | 0.0215 | 8.00 |
| S2 | α_1_ |  | 0.99999952 | 1.80 | 670 |
|  | α_2_ |  |  | 1.76 | 656 |
|  | α_3_ |  |  | 1.76 | 656 |

Table S36. Summarized freshwater characterization factors (CFs) of conventional pesticide from literature [129-131].

| Pesticide number | *CF* range  PAF·m^3^·kg^-1^·d | Max pesticide | Min pesticide |
| --- | --- | --- | --- |
| 19 | 6.10E+02–1.00E+06 | Folpet | Pyraclostrobine |
| 15 | 2.50E+02–1.40E+08 | Metaldehyde | Lambda-cyhalothrin |
| 52 | 2.29E+02–3.95E+08 | Chlormequat-chlorid | Lambda-cyhalothrin |

Table S37. Integrated impact score (*IS*) and life cycle ecotoxicity of IMI. Red marks indicate the scenarios where the freshwater ecological risk of IMI is lower than nano-IMI.

| Scenario | | | *CF*  (PAF·m^3^·kg^-1^·d) | M (kg) | *IS* (CTUe) | LCA Ecotoxicity (CTUe) | Superposition (CTUe) |
| --- | --- | --- | --- | --- | --- | --- | --- |
| IMI | R1 | *EF* | 1.09E+04 | 3.77E-02 | 4.11E+02 | 1.20E+03 | 1.61E+03 |
|  |  | *EF*_1_ | 4.85E+05 |  | 1.83E+04 |  | 1.95E+04 |
|  |  | *EF*_2_ | 1.65E+06 |  | 6.24E+04 |  | 6.36E+04 |
|  |  | *EF*_3_ | 1.37E+07 |  | 5.17E+05 |  | 5.18E+05 |
|  |  | *EF* | 1.09E+04 |  | 4.11E+02 |  | 1.61E+03 |
|  |  | *EF*_1_ | 4.85E+05 |  | 1.83E+04 |  | 1.95E+04 |
|  |  | *EF*_2_ | 1.65E+06 |  | 6.24E+04 |  | 6.36E+04 |
|  |  | *EF*_3_ | 1.37E+07 |  | 5.17E+05 |  | 5.18E+05 |
|  | R2 | *EF* | 1.09E+04 | 3.66E-01 | 3.99E+03 |  | 5.19E+03 |
|  |  | *EF*_1_ | 4.85E+05 |  | 1.77E+05 |  | 1.79E+05 |
|  |  | *EF*_2_ | 1.65E+06 |  | 6.05E+05 |  | 6.06E+05 |
|  |  | *EF*_3_ | 1.37E+07 |  | 5.01E+06 |  | 5.01E+06 |
|  |  | *EF* | 1.09E+04 |  | 3.99E+03 |  | 5.19E+03 |
|  |  | *EF*_1_ | 4.85E+05 |  | 1.77E+05 |  | 1.79E+05 |
|  |  | *EF*_2_ | 1.65E+06 |  | 6.05E+05 |  | 6.06E+05 |
|  |  | *EF*_3_ | 1.37E+07 |  | 5.01E+06 |  | 5.01E+06 |
|  | R3 | *EF* | 1.09E+04 | 4.47E-01 | 4.88E+03 |  | 6.08E+03 |
|  |  | *EF*_1_ | 4.85E+05 |  | 2.17E+05 |  | 2.18E+05 |
|  |  | *EF*_2_ | 1.65E+06 |  | 7.39E+05 |  | 7.40E+05 |
|  |  | *EF*_3_ | 1.37E+07 |  | 6.12E+06 |  | 6.13E+06 |
|  |  | *EF* | 1.09E+04 |  | 4.87E+03 |  | 6.08E+03 |
|  |  | *EF*_1_ | 4.85E+05 |  | 2.17E+05 |  | 2.18E+05 |
|  |  | *EF*_2_ | 1.65E+06 |  | 7.39E+05 |  | 7.40E+05 |
|  |  | *EF*_3_ | 1.37E+07 |  | 6.12E+06 |  | 6.12E+06 |

Table S38. Integrated impact score (*IS*) and life cycle ecotoxicity of nano-IMI (ignoring IMI release from nano-IMI).

| Scenario | | | *CF*  (PAF·m^3^·kg^-1^·d) | M (kg) | *IS* (CTUe) | LCA Ecotoxicity (CTUe) | Superposition (CTUe) |
| --- | --- | --- | --- | --- | --- | --- | --- |
| nano-IMI | R1 | S1 α_1_ | 2.13E+01 | 7.35E-06 | 1.56E-04 | 4.89E+03 | 4.89E+03 |
|  |  | S1 α_2_ | 7.28E+00 | 2.51E-06 | 1.83E-05 |  | 4.89E+03 |
|  |  | S1 α_3_ | 8.00E+00 | 2.76E-06 | 2.21E-05 |  | 4.89E+03 |
|  |  | S2 α_1_ | 6.70E+02 | 6.81E-05 | 4.56E-02 |  | 4.89E+03 |
|  |  | S2 α_2_ | 6.56E+02 | 6.81E-05 | 4.47E-02 |  | 4.89E+03 |
|  |  | S2 α_3_ | 6.56E+02 | 6.81E-05 | 4.47E-02 |  | 4.89E+03 |
|  | R2 | S1 α_1_ | 2.13E+01 | 2.83E-04 | 6.03E-03 |  | 4.89E+03 |
|  |  | S1 α_2_ | 7.28E+00 | 9.69E-05 | 7.05E-04 |  | 4.89E+03 |
|  |  | S1 α_3_ | 8.00E+00 | 1.06E-04 | 8.52E-04 |  | 4.89E+03 |
|  |  | S2 α_1_ | 6.70E+02 | 2.61E-03 | 1.75E+00 |  | 4.89E+03 |
|  |  | S2 α_2_ | 6.56E+02 | 2.61E-03 | 1.71E+00 |  | 4.89E+03 |
|  |  | S2 α_3_ | 6.56E+02 | 2.61E-03 | 1.71E+00 |  | 4.89E+03 |
|  | R3 | S1 α_1_ | 2.13E+01 | 1.31E-03 | 2.79E-02 |  | 4.89E+03 |
|  |  | S1 α_2_ | 7.28E+00 | 4.48E-04 | 3.26E-03 |  | 4.89E+03 |
|  |  | S1 α_3_ | 8.00E+00 | 4.93E-04 | 3.94E-03 |  | 4.89E+03 |
|  |  | S2 α_1_ | 6.70E+02 | 1.18E-02 | 7.90E+00 |  | 4.89E+03 |
|  |  | S2 α_2_ | 6.56E+02 | 1.18E-02 | 7.73E+00 |  | 4.89E+03 |
|  |  | S2 α_3_ | 6.56E+02 | 1.18E-02 | 7.74E+00 |  | 4.89E+03 |

Table S39. Integrated impact score (*IS*) and life cycle ecotoxicity of nano-IMI (considering IMI release from nano-IMI).

| Scenario | | | *CF* | M_1_  (nano-IMI 10%) | M_2_  (release IMI 90%) | *IS* | LCA Ecotoxicity | Superposition |
| --- | --- | --- | --- | --- | --- | --- | --- | --- |
|  |  |  | PAF·m^3^·kg^-1^·d | kg | kg | CTUe | CTUe | CTUe |
| nano-IMI | R1 | S1 α_1_ | 2.13E+01 | 7.35E-07 | 3.15E-06 | 3.44E-02 – 4.32E+01 | 4.89E+03 | 4.89E+03 – 4.93E+03 |
|  |  | S1 α_2_ | 7.28E+00 | 2.51E-07 | 1.08E-06 | 1.18E-02 – 1.48E+01 |  | 4.89E+03 – 4.90E+03 |
|  |  | S1 α_3_ | 8.00E+00 | 2.76E-07 | 1.18E-06 | 1.29E-02 – 1.62E+01 |  | 4.89E+03 – 4.90E+03 |
|  |  | S2 α_1_ | 6.70E+02 | 6.81E-06 | 2.92E-05 | 3.23E-01 – 4.00E+02 |  | 4.89E+03 – 5.29E+03 |
|  |  | S2 α_2_ | 6.56E+02 | 6.81E-06 | 2.92E-05 | 3.23E-01 – 4.00E+02 |  | 4.89E+03 – 5.29E+03 |
|  |  | S2 α_3_ | 6.56E+02 | 6.81E-06 | 2.92E-05 | 3.23E-01 – 4.00E+02 |  | 4.89E+03 – 5.29E+03 |
|  | R2 | S1 α_1_ | 2.13E+01 | 2.83E-05 | 1.21E-04 | 1.33E+00 – 1.66E+03 |  | 4.89E+03 – 6.55E+03 |
|  |  | S1 α_2_ | 7.28E+00 | 9.69E-06 | 4.15E-05 | 4.53E-01 – 5.69E+02 |  | 4.89E+03 – 5.45E+03 |
|  |  | S1 α_3_ | 8.00E+00 | 1.06E-05 | 4.57E-05 | 4.98E-01 – 6.26E+02 |  | 4.89E+03 – 5.51E+03 |
|  |  | S2 α_1_ | 6.70E+02 | 2.61E-04 | 1.12E-03 | 1.24E+01 – 1.53E+04 |  | 4.90E+03 – 2.02E+04 |
|  |  | S2 α_2_ | 6.56E+02 | 2.61E-04 | 1.12E-03 | 1.24E+01 – 1.53E+04 |  | 4.90E+03 – 2.02E+04 |
|  |  | S2 α_3_ | 6.56E+02 | 2.61E-04 | 1.12E-03 | 1.24E+01 – 1.53E+04 |  | 4.90E+03 – 2.02E+04 |
|  | R3 | S1 α_1_ | 2.13E+01 | 1.31E-04 | 5.61E-04 | 6.13E+00 – 7.69E+03 |  | 4.89E+03 – 1.26E+04 |
|  |  | S1 α_2_ | 7.28E+00 | 4.48E-05 | 1.92E-04 | 2.10E+00 – 2.63E+03 |  | 4.89E+03 – 7.52E+03 |
|  |  | S1 α_3_ | 8.00E+00 | 4.93E-05 | 2.11E-04 | 2.30E+00 – 2.89E+03 |  | 4.89E+03 – 7.78E+03 |
|  |  | S2 α_1_ | 6.70E+02 | 1.18E-03 | 5.06E-03 | 5.60E+01 – 6.93E+04 |  | 4.94E+03 – 7.42E+04 |
|  |  | S2 α_2_ | 6.56E+02 | 1.18E-03 | 5.06E-03 | 5.59E+01 – 6.93E+04 |  | 4.94E+03 – 7.42E+04 |
|  |  | S2 α_3_ | 6.56E+02 | 1.18E-03 | 5.06E-03 | 5.59E+01 – 6.93E+04 |  | 4.94E+03 – 7.42E+04 |

**FIGURES**


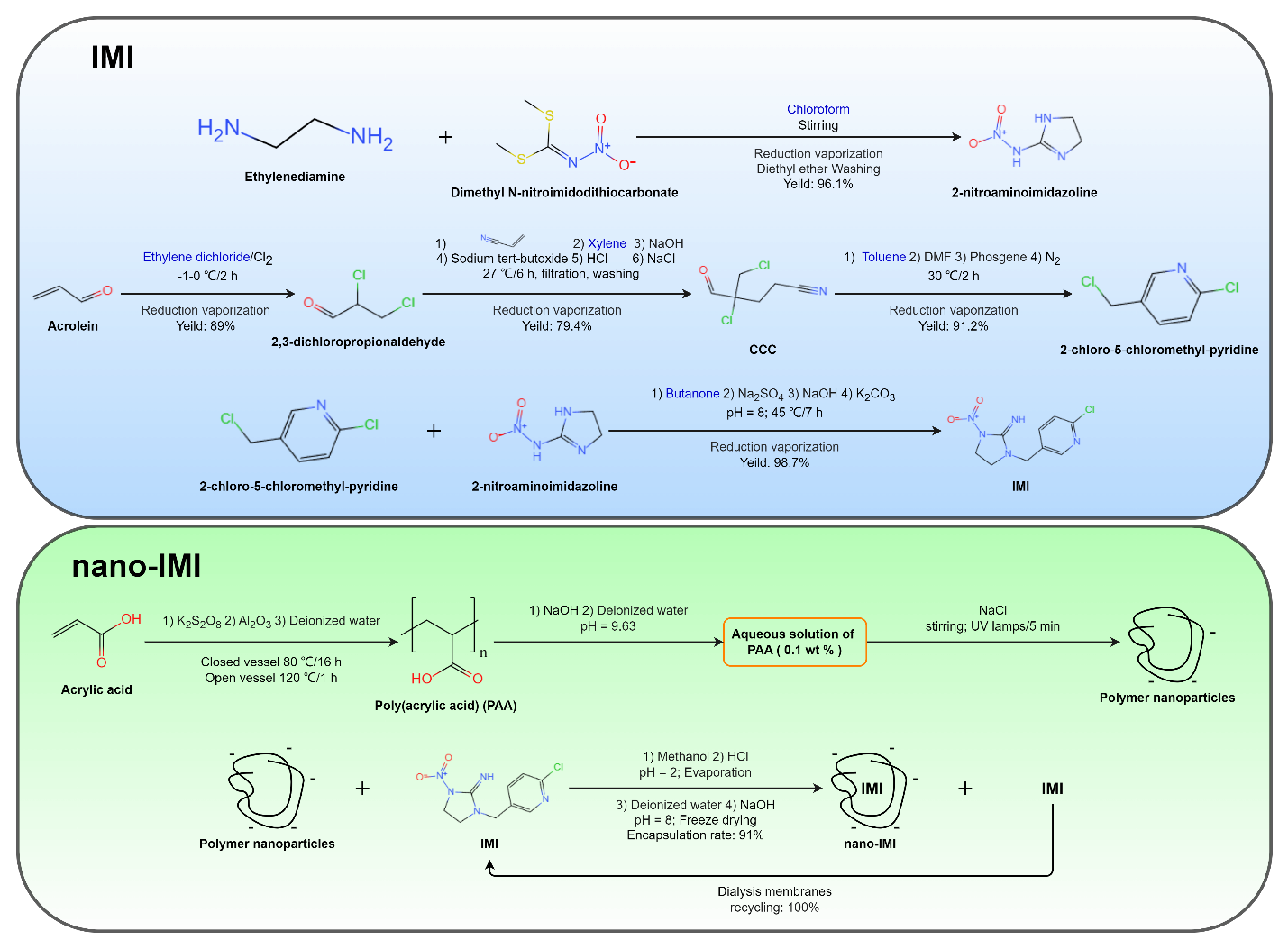
 Figure S1. Main process flow of IMI and nano-IMI production.


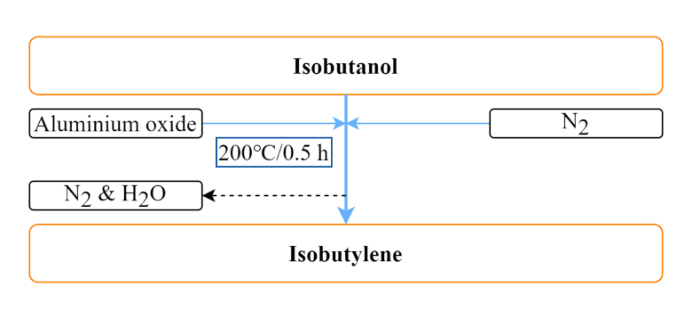


Figure S2. Scheme for isobutylene production [20].


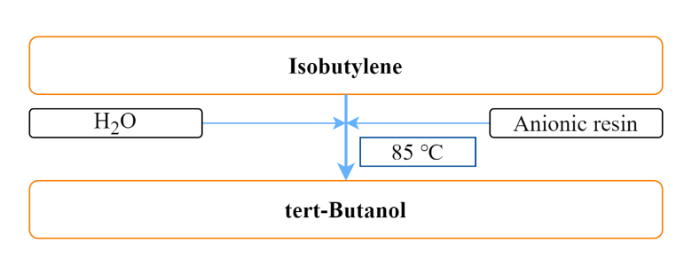


Figure S3. Scheme for tert-butanol production [21].


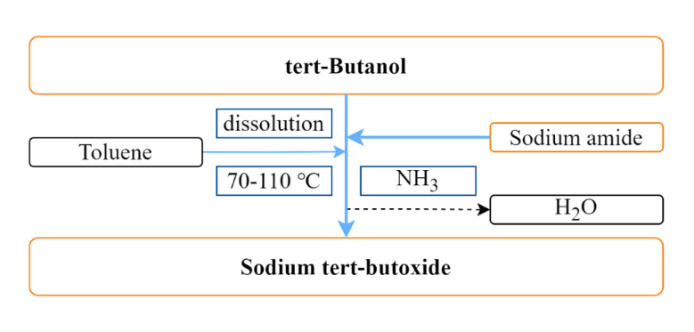


Figure S4. Scheme for sodium tert-butoxide production [22].


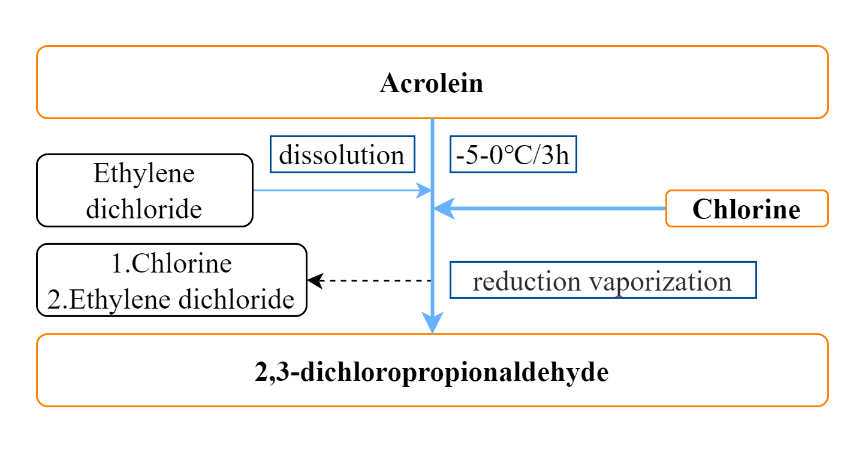


Figure S5. Scheme for 2,3-dichloropropionaldehyde (CAS: 10140-89-3)

production [23].


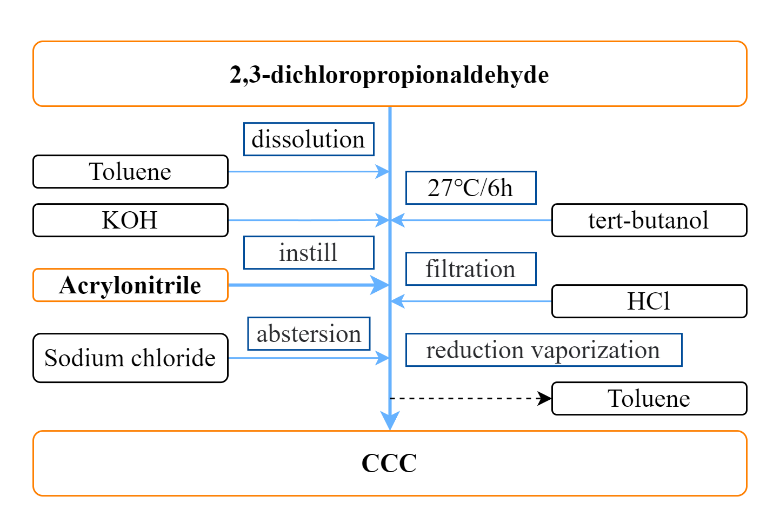


Figure S6. Scheme for 4-chloro-4-(chloromethyl)-5-oxopentanenitrile

(CCC, CAS: 150807-86-6) production [23].


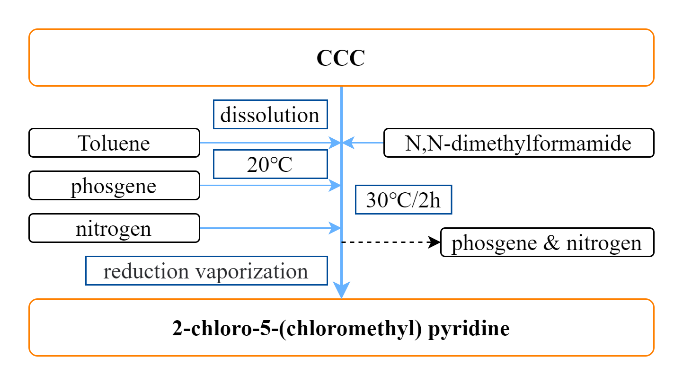


Figure S7. Scheme for 2-chloro-5-chloromethylpyridine (CAS: 70258-18-3)

production [1].


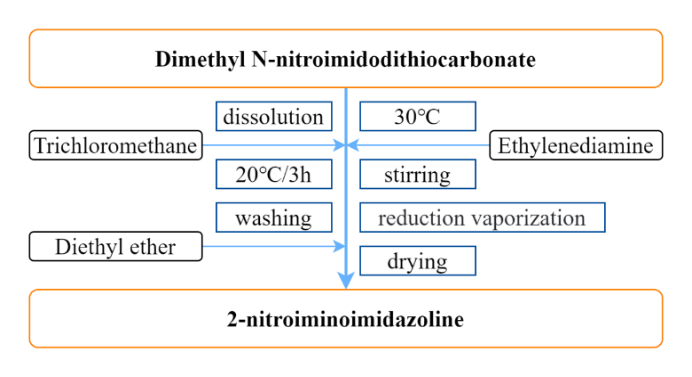


Figure S8. Scheme for 2-nitroaminoimidazoline (CAS: 5465-96-3)

production [2].


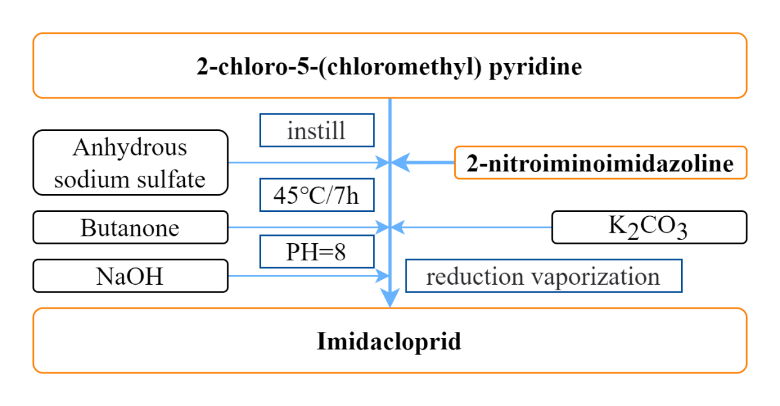


Figure S9. Scheme for the active ingredient production of imidacloprid (IMI) [3].


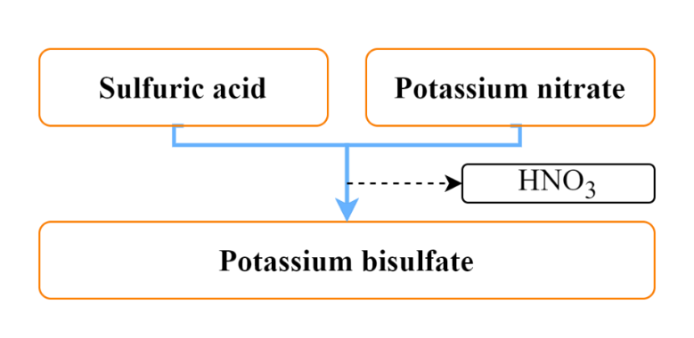


Figure S10. Scheme for potassium bisulfate production [6].


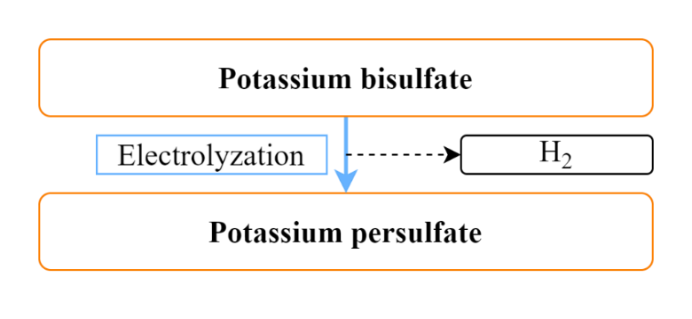


Figure S11. Scheme for potassium persulfate production [6].


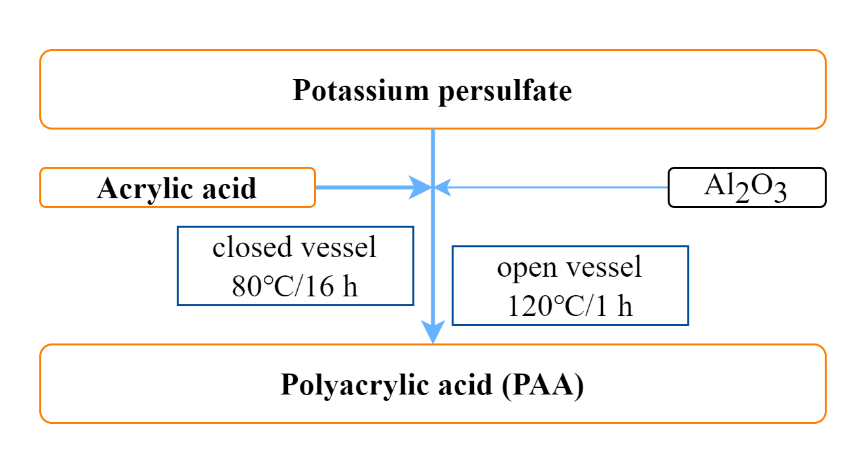


Figure S12. Scheme for polyacrylic acid (PAA) production [5].


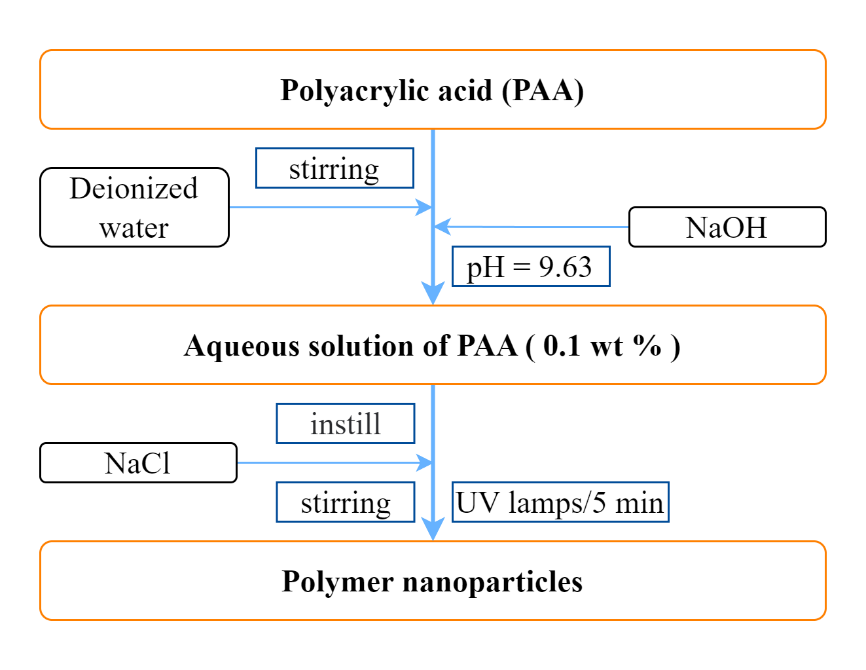


Figure S13. Scheme for polymer nanoparticles production [4].


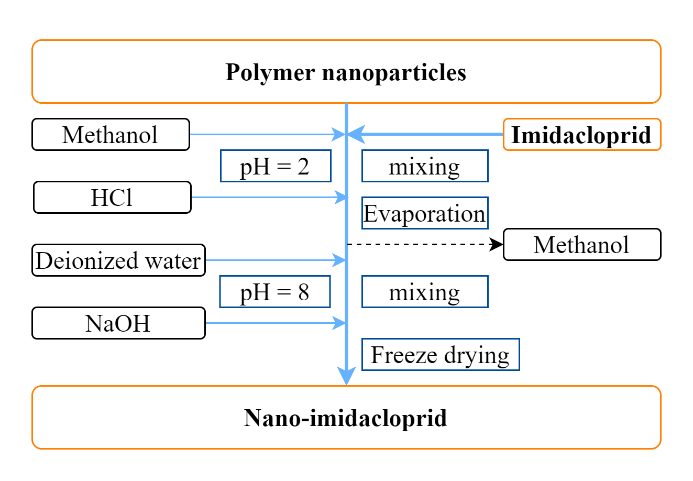


Figure S14. Scheme for the active ingredient (contain the nanocarrier) production of

nano-imidacloprid (nano-IMI) [4].


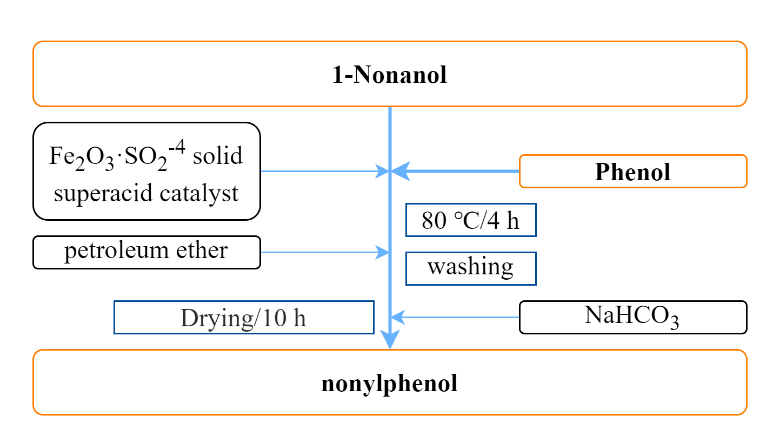


Figure S15. Scheme for nonylphenol (CAS: 25154-52-3) production [25].


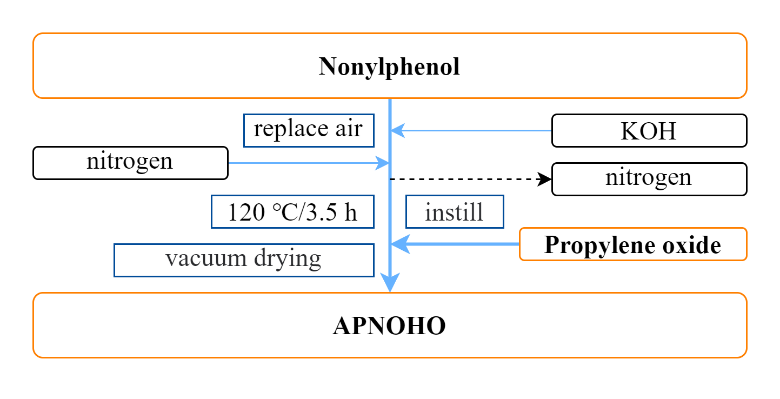


Figure S16. Scheme for polyethylene glycol nonylphenyl ether (APNOHO, CAS: 9016-45-9) production [11].


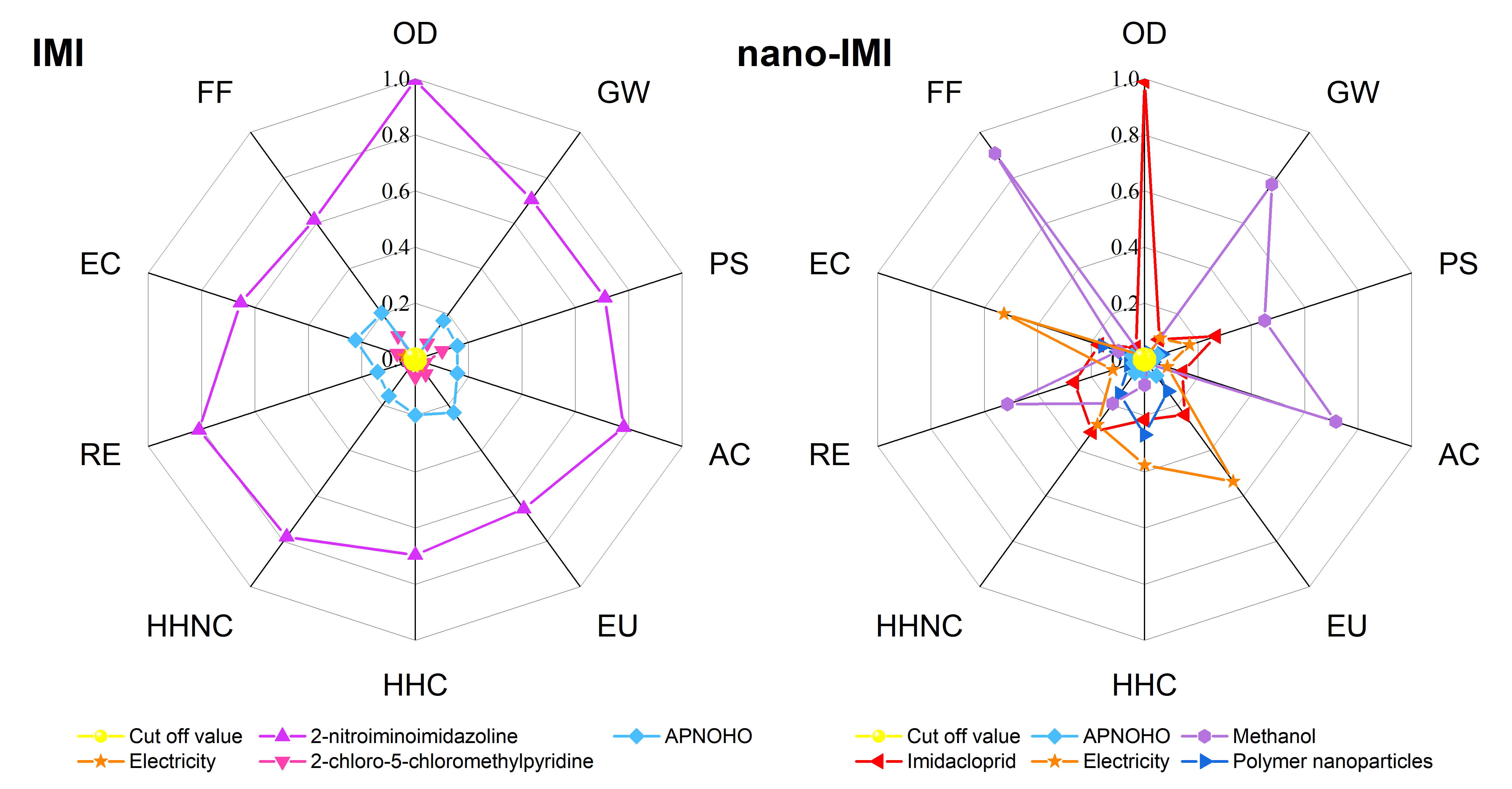


Figure S17. Sensitivity analysis of IMI and nano-IMI production. OD = ozone depletion; GW = global warming potential; PS = smog; AC = acidification; EU = eutrophication; HHC = carcinogenic; HHNC = noncarcinogenic; RE = respiratory effects; EC = ecotoxicity; FF = fossil fuel depletion. Detailed data are listed in Tables S19–S20.


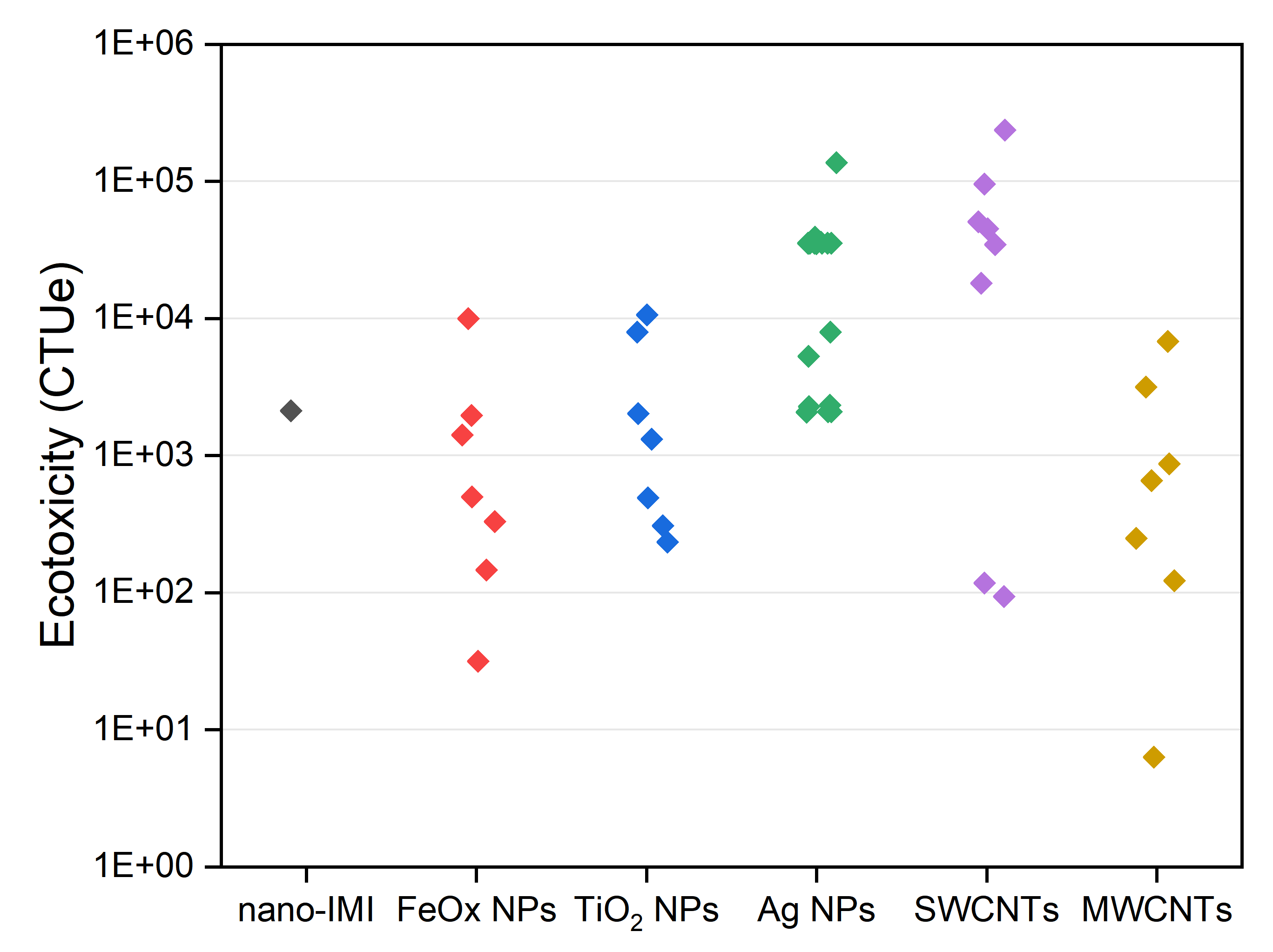


Figure S18. Ecotoxicity of nano-IMI and ENPs during life cycle production [132-136].


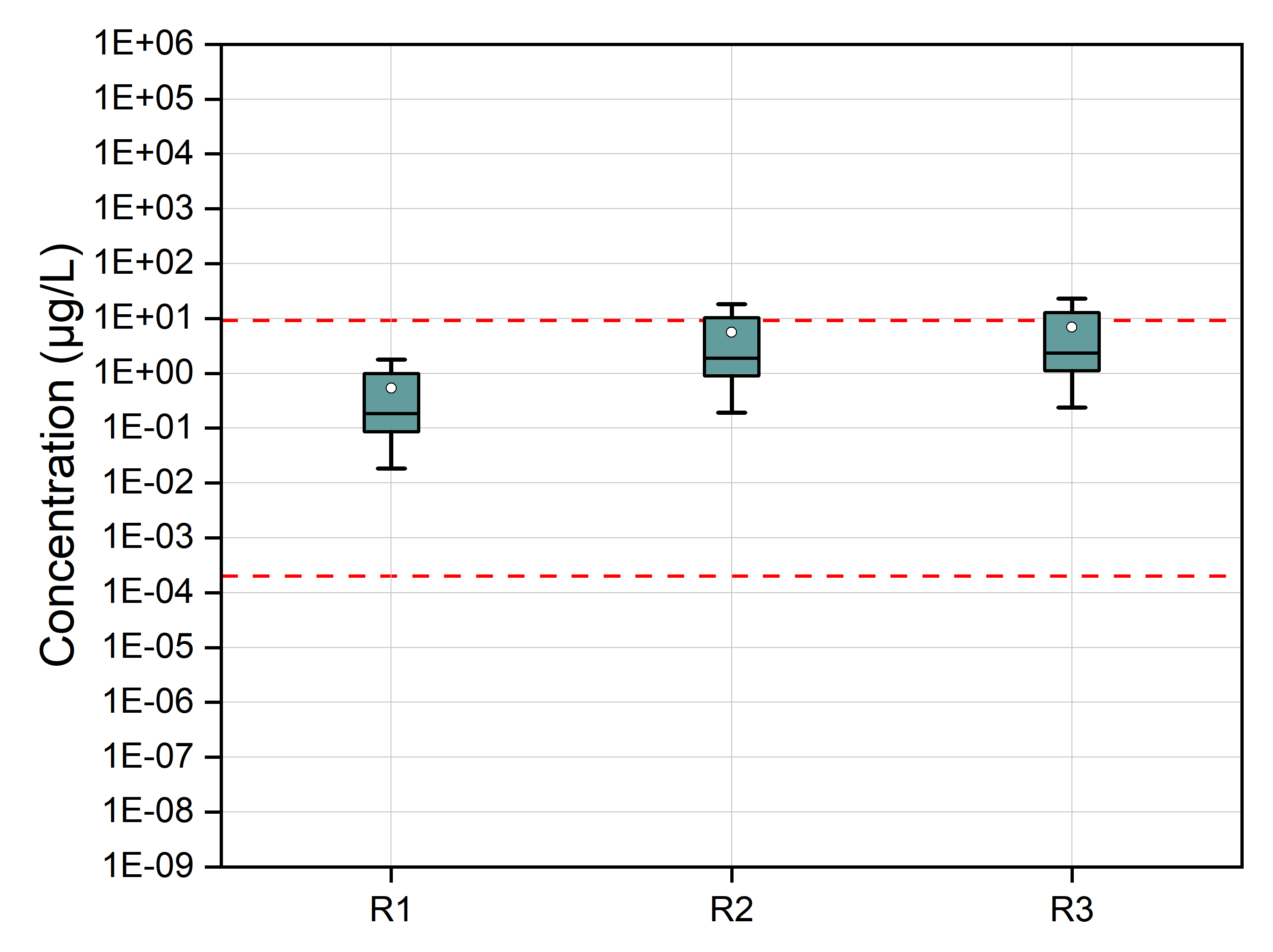


Figure S19. Prediction environmental concentration (PEC) of IMI considering multiple factors (Pesticide drift, plant absorption, proportion of agricultural soil at continental scale, etc.). The red dashed line represents the range of measured environment concentration (MEC) of IMI in fresh water adjacent agricultural area.

**Reference**

[1] L. Xin,L. Xuliang Green synthesis method of 2-chloro-5-chloromethylpyridine. CN 110483382 A, 2019/09/18, 2019.

[2] S. Kojima, M. Funabora, N. Kawahara,Y. Iiyoshi Nitroimino compound as intermediate for insecticides and pharmaceuticals. US 5453529 A, 1993/11/23, 1995.

[3] Z. Ming Production improvement process of imidacloprid. CN 103641815 A, 2013/11/21, 2014.

[4] F. Li, H. Pham,J. Anderson Darren Methods to formulate neutral organic compounds with polymer nanoparticles. US 2020/0260724 A1, 2020/02/12, 2020.

[5] V. Bastiaan Maarten, A. Bergwerff Jacob, J. Van Oene,J. A. N. Tromp Henk Supported hydrotreating catalysts having enhanced activity. WO 2014/056846 A1, 2013/10/07, 2014.

[6] P. Patnaik, Handbook of inorganic chemicals, McGraw-Hill New York, 2003, Vol. 529, pp. 769-771.

[7] N. Memarizadeh, M. Ghadamyari, M. Adeli,K. Talebi, Preparation, characterization and efficiency of nanoencapsulated imidacloprid under laboratory conditions, Ecotoxicol. Environ. Saf. 107 (2014) 77–83.

[8] Y. Liu, Z. Tong,R. K. Prud'homme, Stabilized polymeric nanoparticles for controlled and efficient release of bifenthrin, Pest Manage. Sci. 64 (8) (2008) 808–812.

[9] L. E. Beane Freeman, Invited perspective: Pesticide adjuvants and inert ingredients — a missing piece of the puzzle, Environ. Health Perspect. 130 (8) (2022) 81301.

[10] C. Cox,M. Zeiss, Health, pesticide adjuvants, and inert ingredients: California case study illustrates need for data access, Environ. Health Perspect. 130 (8) (2022) 085001.

[11] H. Zhiqiang, J. Guangyong, F. Yun,C. Ji, A novel technique to enhance selectivity of sulfation against sulfonation using katastaltic chlorosulfonic acid, Fine Chemicals 30 (07) (2013) 725-728+765.

[12] AmTide Limited Liability Company., AmTide Imidacloprid 2F Insecticide. <http://cues.cfans.umn.edu/old/cwlb/labels/AmTide2F.pdf> (accessed 2023-06-20).

[13] Vive Crop Protection, Viloprid FC 1.7 insecticide. <https://www.vivecrop.com/products/vilopridfc1.7> (accessed 2023-06-20).

[14] J. Wang, R. Yin, Y. Liu, B. Wang, N. Wang, P. Xiao, T. Xiao,H. Hirai, Meta-analysis of neonicotinoid insecticides in global surface waters, Environ. Sci. Pollut. Res. 30 (1) (2023) 1039-1047.

[15] P. Fantke, M. Bijster, C. Guignard, M. Z. Hauschild, M. A. J. Huijbregts, O. Jolliet, A. Kounina, V. Magaud, M. Margni, T. McKone, L. Posthuma, R. K. Rosenbaum, D. van de Meent, R. van Zelm,P. Fantke, USEtox 2.0: Documentation (Version 1), USEtox International Center, 2017.

[16] S. Raimondo, B. J. Montague,M. G. Barron, Determinants of variability in acute to chronic toxicity ratios for aquatic invertebrates and fish, Environ. Toxicol. Chem. 26 (9) (2007) 2019-23.

[17] Y. Y. L. Wang, J. Xiong, O. E. Ohore, Y. E. Cai, H. Fan, E. Sanganyado, P. Li, J. You, W. Liu,Z. Wang, Deriving freshwater guideline values for neonicotinoid insecticides: Implications for water quality guidelines and ecological risk assessment, Sci. Total Environ. 828 (2022) 154569.

[18] R. K. Rosenbaum, M. Margni,O. Jolliet, A flexible matrix algebra framework for the multimedia multipathway modeling of emission to impacts, Environ. Int. 33 (5) (2007) 624-634.

[19] Y. L. Deng, J. Y. Li, M. Qiu, F. Yang, J. Y. Zhang,C. Yuan, Deriving characterization factors on freshwater ecotoxicity of graphene oxide nanomaterial for life cycle impact assessment, Int. J. Life Cycle Assess. 22 (2017) 222–236.

[20] T. SUZUKI, A. TAKE-DA, T. YASUKAWA, W. NINOM-IYA,K. OOYACHI Catalyst for isobutylene production and method for producing isobutylene. JP WO2016171178 A1, 2016/04/20, 2017.

[21] K. U. N. Liu, M. Han, Y. Zhao, H. Li, Z. Gao,J. Zhao Production system and method for preparing methyl methacrylate from low-purity isobutylene. CN 108863789 A, 2018/06/14, 2018.

[22] S. Tang,Z. Duan, New synthesis technology of sodium tert-Butoxide, Modern Agrochemicals (01) (2004) 9-19.

[23] Q. Xu, Z. Wu, J. Wu, Y. Xiong, L. An,A. Jin Method for preparing precursor 2-chloro-2-chloromethyl-4-cyano butyraldehyde of Paichonghding intermediate. CN 102766070 B, 2012/06/06, 2014.

[24] J. Jovanovic,B. Adnadjevic, Influence of microwave heating on the kinetic of acrylic acid polymerization and crosslinking, J Appl Polym Sci 116 (1) (2010) 55-63.

[25] Y. Su, S. Zhu,J. Wang, Catalytic synthesis of long chain alkylphenol by Fe_2_O_3_·SO_4_^2-^, Shiyou Xuebao, Shiyou Jiagong (05) (2006) 92-95.

[26] Parr Instrument Company, Series 4560 Mini Reactors, 100-600 mL Specifications. <https://www.parrinst.com/products/stirred-reactors/series-4560-100-600-ml-mini-reactors/specifications/> (accessed 2023-04-04).

[27] IKA, IKA Rotary Evaporators RV 10 auto pro V. <https://www.ika.com/en/Products-Lab-Eq/Rotary-Evaporators-Rotary-evaporator-distilling-distillation-csp-35/RV-10-auto-pro-V-cpdt-10004815/> (accessed 2023-04-04).

[28] Orioner, Low-temperature (constant-temperature) Stirring Reaction Bath. <https://www.orioner.com/Low-temperature-(constant-temperature)-Stirring-Reaction-Bath> (accessed 2023-04-04).

[29] Hangzhou Miu Instruments co. ltd., NDK200-1N sample concentrator. <http://miulab.com/cn/product/NDK200-2N.html> (accessed 2023-04-04).

[30] CAPP, CAPPRondo magnetic stirrer CRS-15X. <https://capp.dk/files/userguide/CRS-15X_CappRondo_Magnetic_Stirrer_UG_0.pdf> (accessed 2023-04-04).

[31] Enapter AG, Enapter Datasheet AEM Electrolyser EL 4.0 AC air cooled. <https://handbook.enapter.com/electrolyser/el40/downloads/Enapter_Datasheet_EL40_EN.pdf> (accessed 2023-04-04).

[32] CEM Corporation, Parallel & Scale-Up Microwave Synthesizer - MARS 6 Synthesis. <https://cem.com/en/mars-6-synthesis> (accessed 2023-04-04).

[33] Thermo Fisher Scientific, Thermo Scientific™ 3UV Lamp. <https://www.fishersci.nl/shop/products/pierce-uvp-3uv-ultraviolet-lamp-1/10531194#?keyword> (accessed 2023-04-04).

[34] SciQuip, CHRIST BETA 2-8-85 ℃ LSCBASIC 8kg freeze dryers. <https://sciquip.co.uk/christ-beta-1-8-55-c-lscbasic-2-8-85-c-lscbasic-8kg-freeze-dryers.html> (accessed 2023-04-04).

[35] VELP Scientifica, MULTI-TX5 digital multi-tube vortex. <https://www.velp.com/en-ww/multi-tx5-digital-multi-tube-vortex.aspx> (accessed 2023-04-04).

[36] Weihai Automatically-controlled Reaction Kettle co. ltd., WHF－0.5 L high magnetic driven autoclave. <http://www.whweiba.cn/pddetaildate/product/detail/20130615_17649556.html> (accessed 2023-04-04).

[37] F. Wu, S. Zhang, H. Li, P. Liu, H. Su, Y. Zhang, B. W. Brooks,J. You, Toxicokinetics explain differential freshwater ecotoxicity of nanoencapsulated imidacloprid compared to its conventional active ingredient, Environ. Sci. Technol. 58 (22) (2024) 9548–9558.

[38] M. Raby, M. Nowierski, D. Perlov, X. Zhao, C. Hao, D. G. Poirier,P. K. Sibley, Acute toxicity of 6 neonicotinoid insecticides to freshwater invertebrates, Environ. Toxicol. Chem. 37 (5) (2018) 1430-1445.

[39] M. D. Pavlaki, A. L. G. Ferreira, A. M. V. M. Soares,S. Loureiro, Changes of chemical chronic toxicity to *Daphnia magna* under different food regimes, Ecotoxicol. Environ. Saf. 109 (2014) 48-55.

[40] M. Y. Song, J. D. Stark,J. J. Brown, Comparative toxicity of four insecticides, including imidacloprid and tebufenozide, to four aquatic arthropods, Environ. Toxicol. Chem. 16 (12) (1997) 2494-2500.

[41] D. Hayasaka, T. Korenaga, K. Suzuki, F. Sánchez-Bayo,K. Goka, Differences in susceptibility of five cladoceran species to two systemic insecticides, imidacloprid and fipronil, Ecotoxicology 21 (2) (2012) 421-427.

[42] M. D. Pavlaki, R. Pereira, S. Loureiro,A. M. V. M. Soares, Effects of binary mixtures on the life traits of *Daphnia magna*, Ecotoxicol. Environ. Saf. 74 (1) (2011) 99-110.

[43] J. Chevalier, E. Harscoët, M. Keller, P. Pandard, J. Cachot,M. Grote, Exploration of *Daphnia* behavioral effect profiles induced by a broad range of toxicants with different modes of action, Environ. Toxicol. Chem. 34 (8) (2015) 1760-1769.

[44] T. Tisler, A. Jemec, B. Mozetic,P. Trebse, Hazard identification of imidacloprid to aquatic environment, Chemosphere 76 (7) (2009) 907-914.

[45] O. Ieromina, W. J. G. M. Peijnenburg, G. de Snoo, J. Müller, T. P. Knepper,M. G. Vijver, Impact of imidacloprid on *Daphnia magna* under different food quality regimes, Environ. Toxicol. Chem. 33 (3) (2014) 621-631.

[46] F. Sanchez-Bayo,K. Goka, Influence of light in acute toxicity bioassays of imidacloprid and zinc pyrithione to zooplankton crustaceans, Aquat Toxicol 78 (3) (2006) 262-271.

[47] S. Z. Qi, D. H. Wang, L. Z. Zhu, M. M. Teng, C. J. Wang, X. F. Xue,L. M. Wu, Neonicotinoid insecticides imidacloprid, guadipyr, and cycloxaprid induce acute oxidative stress in *Daphnia magna*, Ecotoxicol. Environ. Saf. 148 (2018) 352-358.

[48] USEPA, Pesticide Ecotoxicity Database [Formerly: Environmental Effects Database (EEDB)], Office of Pesticide Programs, Environmental Fate and Effects Division, Washington, DC. (2000).

[49] J. L. T. Pestana, S. Loureiro, D. J. Baird,A. M. M. Soares, Pesticide exposure and inducible antipredator responses in the zooplankton grazer, *Daphnia magna* Straus, Chemosphere 78 (3) (2010) 241-248.

[50] M. Raby, X. M. Zhao, C. Y. Hao, D. G. Poirier,P. K. Sibley, Relative chronic sensitivity of neonicotinoid insecticides to *Ceriodaphnia dubia* and *Daphnia magna*, Ecotoxicol. Environ. Saf. 163 (2018) 238-244.

[51] S. Loureiro, C. Svendsen, A. L. G. Ferreira, C. Pinheiro, F. Ribeiro,A. M. V. M. Soares, Toxicity of three binary mixtures to *Daphnia magna*: Comparing chemical modes of action and deviations from conceptual models, Environ. Toxicol. Chem. 29 (8) (2010) 1716-1726.

[52] Y. M. Chang, L. G. Mao, L. Zhang, Y. N. Zhang,H. Y. Jiang, Combined toxicity of imidacloprid, acetochlor, and tebuconazole to zebrafish (*Danio rerio*): acute toxicity and hepatotoxicity assessment, Environ. Sci. Pollut. Res. 27 (10) (2020) 10286-10295.

[53] Y. H. Wang, G. L. Yang, D. J. Dai, Z. L. Xu, L. M. Cai, Q. Wang,Y. J. Yu, Individual and mixture effects of five agricultural pesticides on zebrafish (*Danio rerio*) larvae, Environ. Sci. Pollut. Res. 24 (5) (2017) 4528-4536.

[54] S. G. Wu, X. F. Li, X. J. Liu, G. L. Yang, X. H. An, Q. Wang,Y. H. Wang, Joint toxic effects of triazophos and imidacloprid on zebrafish (*Danio rerio*), Environ. Pollut. 235 (2018) 470-481.

[55] F. Sánchez-Bayo,K. Goka, Evaluation of suitable endpoints for assessing the impacts of toxicants at the community level, Ecotoxicology 21 (3) (2012) 667-680.

[56] S. Lukancic, U. Zibrat, T. Mezek, A. Jerebic, T. Simcic,A. Brancelj, A new method for early assessment of effects of exposing two non-target crustacean species, *Asellus aquaticus* and *Gammarus fossarum*, to pesticides, a laboratory study, Toxicol Ind Health 26 (4) (2010) 217-228.

[57] P. J. Van den Brink, J. M. Van Smeden, R. S. Bekele, W. Dierick, D. M. De Gelder, M. Noteboom,I. Roessink, Acute and chronic toxicity of neonicotinoids to nymphs of a mayfly species and some notes on seasonal differences, Environ. Toxicol. Chem. 35 (1) (2016) 128-33.

[58] S. Lukancic, U. Zibrat, T. Mezek, A. Jerebic, T. Simcic,A. Brancelj, Effects of exposing two non-target crustacean species, *Asellus aquaticus* L., and *Gammarus fossarum* Koch., to atrazine and imidacloprid, B Environ Contam Tox 84 (1) (2010) 85-90.

[59] J. Augusiak,P. J. Van den Brink, The influence of insecticide exposure and environmental stimuli on the movement behaviour and dispersal of a freshwater isopod, Ecotoxicology 25 (7) (2016) 1338-1352.

[60] I. Roessink, L. B. Merga, H. J. Zweers,P. J. Van den Brink, The neonicotinoid imidacloprid shows high chronic toxicity to mayfly nymphs, Environ. Toxicol. Chem. 32 (5) (2013) 1096-1100.

[61] X. D. Chen, E. Culbert, V. Hebert,J. D. Stark, Mixture effects of the nonylphenyl polyethoxylate, R-11 and the insecticide, imidacloprid on population growth rate and other parameters of the crustacean, *Ceriodaphnia dubia*, Ecotoxicol. Environ. Saf. 73 (2) (2010) 132-137.

[62] H. A. Hassoon,S. A. Salman, The acute effect of pesticides carbaryl and imidacloprid on *Daphnia pulex* species, J. Int. Environ. Appl. Sci. 11 (1) (2016) 18-25.

[63] K. A. Sumon, A. K. Ritika, E. Peeters, H. Rashid, R. H. Bosma, M. S. Rahman, M. K. Fatema,P. J. Van den Brink, Effects of imidacloprid on the ecology of sub-tropical freshwater microcosms, Environ. Pollut. 236 (2018) 432-441.

[64] Y. H. Hong, Y. Huang, S. Wu, X. Z. Yang, Y. Z. Dong, D. Y. Xu,Z. Q. Huang, Effects of imidacloprid on the oxidative stress, detoxification and gut microbiota of Chinese mitten crab, *Eriocheir* *sinensis*, Sci. Total Environ. 729 (2020) 138276.

[65] D. Englert, J. P. Zubrod, M. Link, S. Mertins, R. Schulz,M. Bundschuh, Does waterborne exposure explain effects caused by neonicotinoid-contaminated plant material in aquatic systems?, Environ. Sci. Technol. 51 (10) (2017) 5793-5802.

[66] R. Ashauer, A. Hintermeister, E. Potthoff,B. I. Escher, Acute toxicity of organic chemicals to *Gammarus pulex* correlates with sensitivity of *Daphnia magna* across most modes of action, Aquat Toxicol 103 (1-2) (2011) 38-45.

[67] R. Ashauer, I. Caravatti, A. Hintermeister,B. I. Escher, Bioaccumulation kinetics of organic xenobiotic pollutants in the freshwater invertebrate *Gammarus pulex* modeled with prediction intervals, Environ. Toxicol. Chem. 29 (7) (2010) 1625-1636.

[68] A. Agatz, R. Ashauer,C. D. Brown, Imidacloprid perturbs feeding of *Gammarus pulex* at environmentally relevant concentrations, Environ. Toxicol. Chem. 33 (3) (2014) 648-653.

[69] M. A. Beketov,M. Liess, Potential of 11 pesticides to initiate downstream drift of stream macroinvertebrates, Arch Environ Con Tox 55 (2) (2008) 247-253.

[70] R. Böttger, J. Schaller,S. Mohr, Closer to reality — the influence of toxicity test modifications on the sensitivity of *Gammarus roeseli* to the insecticide imidacloprid, Ecotoxicol. Environ. Saf. 81 (2012) 49-54.

[71] S. J. Stoughton, K. Liber, J. Culp,A. Cessna, Acute and chronic toxicity of imidacloprid to the aquatic invertebrates *Chironomus tentans* and *Hyalella azteca* under constant- and pulse-exposure conditions, Arch Environ Con Tox 54 (4) (2008) 662-673.

[72] A. J. Bartlett, A. M. Hedges, K. D. Intini, L. R. Brown, F. J. Maisonneuve, S. A. Robinson, P. L. Gilliss,S. R. de Solla, Acute and chronic toxicity of neonicotinoid and butenolide insecticides to the freshwater amphipod, *Hyalella azteca*, Ecotoxicol. Environ. Saf. 175 (2019) 215-223.

[73] M. Raby, E. Maloney, D. G. Poirier,P. K. Sibley, Acute effects of binary mixtures of imidacloprid and tebuconazole on 4 freshwater invertebrates, Environ. Toxicol. Chem. 38 (5) (2019) 1093-1103.

[74] M. Lanteigne, S. A. Whiting,M. J. Lydy, Mixture toxicity of imidacloprid and cyfluthrin to two non-target species, the fathead minnow *Pimephales promelas* and the amphipod *Hyalella azteca*, Arch Environ Con Tox 68 (2) (2015) 354-361.

[75] J. A. Frew,C. E. Grue, Assessing the risk to green sturgeon from application of imidacloprid to control burrowing shrimp in Willapa Bay, Washington—Part II: Controlled exposure studies, Environ. Toxicol. Chem. 34 (11) (2015) 2542-8.

[76] J. A. Frew. Environmental and systemic exposure assessment for green sturgeon following application of imidacloprid for the control of burrowing shrimp in Willapa Bay, Washington. 2014.

[77] M. Grădilă, Chronic aspects of imidacloprid on the fishes from *Cyprinidae* family, Rom. J. Plant Prot. 6 (2013) 11-15.

[78] J. U. Nnadi, I. N. Dimelu, S. I. Nwani, J. C. Madu, C. I. Atama, G. N. Attamah, J. I. Okwor,C. D. Nwani, Biometric variations and oxidative stress responses in juvenile *Clarias gariepinus* exposed to Termex®, Afr J Aquat Sci 43 (1) (2018) 27-34.

[79] M. A. Islam, M. S. Hossen, K. A. Sumon,M. M. Rahman, Acute toxicity of imidacloprid on the developmental stages of common carp *Cyprinus carpio*, Toxicol Env Health 11 (3) (2019) 244-251.

[80] S. Qadir, A. Latif, M. Ali,F. Iqbal, Effects of Imidacloprid on the hematological and serum biochemical profile of *Labeo rohita*, Pak J Zool 46 (4) (2014) 1085-1090.

[81] X. H. Xia, X. P. Xia, W. R. Huo, H. Dong, L. X. Zhang,Z. J. Chang, Toxic effects of imidacloprid on adult loach (*Misgurnus anguillicaudatus*), Environ Toxicol Phar 45 (2016) 132-139.

[82] J. L. C. Vitualla, P. P. Ocampo,O. E. Matalog, Morphometrics of hepatic and splenic melanomacrophage centers (MMCS) of nile tilapia (*Oreochromis niloticus* Linn.) after exposure to imidacloprid, Philipp. Entomol. 28 (2).

[83] M. A. E. Naiel, N. E. M. Ismael, S. A. A. Abd El-hameed,M. S. Amer, The antioxidative and immunity roles of chitosan nanoparticle and vitamin C-supplemented diets against imidacloprid toxicity on *Oreochromis niloticus*, Aquaculture 523 (2020) 735219.

[84] C. Ruiz de Arcaute, J. M. Pérez-Iglesias, N. Nikoloff, G. S. Natale, S. Soloneski,M. L. Larramendy, Genotoxicity evaluation of the insecticide imidacloprid on circulating blood cells of Montevideo tree frog *Hypsiboas pulchellus* tadpoles (Anura, Hylidae) by comet and micronucleus bioassays, Ecol Indic 45 (2014) 632-639.

[85] J. M. Pérez-Iglesias, C. R. de Arcaute, N. Nikoloff, L. Dury, S. Soloneski, G. S. Natale,M. L. Larramendy, The genotoxic effects of the imidacloprid-based insecticide formulation Glacoxan Imida on Montevideo tree frog *Hypsiboas pulchellus* tadpoles (Anura, Hylidae), Ecotoxicol. Environ. Saf. 104 (2014) 120-126.

[86] S. L. Feng, Z. M. Kong, X. M. Wang, L. R. Zhao,P. G. Peng, Acute toxicity and genotoxicity of two novel pesticides on amphibian, *Rana N. Hallowell*, Chemosphere 56 (5) (2004) 457-463.

[87] M. Saka,N. Tada, Acute and chronic toxicity tests of systemic insecticides, four neonicotinoids and fipronil, using the tadpoles of the western clawed frog *Silurana tropicalis*, Chemosphere 270 (2021) 129418.

[88] R. S. Prosser, S. R. de Solla, E. A. M. Holman, R. Osborne, S. A. Robinson, A. J. Bartlett, F. J. Maisonneuve,P. L. Gillis, Sensitivity of the early-life stages of freshwater mollusks to neonicotinoid and butenolide insecticides, Environ. Pollut. 218 (2016) 428-435.

[89] A. C. Alexander, J. M. Culp, K. Liber,A. J. Cessna, Effects of insecticide exposure on feeding inhibition in mayflies and oligochaetes, Environ. Toxicol. Chem. 26 (8) (2007) 1726-1732.

[90] V. Contardo-Jara,M. O. Gessner, Uptake and physiological effects of the neonicotinoid imidacloprid and its commercial formulation Confidor® in a widespread freshwater oligochaete, Environ. Pollut. 264 (2020) 114793.

[91] P. Lekvongphiboon,N. Praphairaksit, Combined toxicity of imidacloprid and cadmium on histopathology and acetylcholinesterase activity in aquatic oligochaetes (*Tubifex tubifex* Müller, 1774), Environ Geochem Hlth 42 (10) (2020) 3431-3441.

[92] A. Gerhardt, Screening the toxicity of Ni, Cd, Cu, ivermectin, and imidacloprid in a short-term automated behavioral toxicity test with *Tubifex tubifex* (Müller 1774) (Oligochaeta), Hum Ecol Risk Assess 15 (1) (2009) 27-40.

[93] J. W. Pridgeon, J. J. Becnel, G. C. Clark,K. J. Linthicum, A high-throughput screening method to identify potential pesticides for mosquito control, J Med Entomol 46 (2) (2009) 335-341.

[94] S. Uragayala, V. Verma, E. Natarajan, P. S. Velamuri,R. Kamaraju, Adulticidal & larvicidal efficacy of three neonicotinoids against insecticide susceptible & resistant mosquito strains, Indian J Med Res 142 (Suppl 1) (2015) 64-70.

[95] C. F. Marina, J. G. Bond, J. Muñoz, J. Valle, H. Quiroz-Martínez, J. A. Torres-Monzón,T. Williams, Efficacy of larvicides for the control of dengue, Zika, and chikungunya vectors in an urban cemetery in southern Mexico, Parasitol Res 117 (6) (2018) 1941-1952.

[96] A. Paul, L. C. Harrington,J. G. Scott, Evaluation of novel insecticides for control of dengue vector *Aedes aegypti* (Diptera: Culicidae), J Med Entomol 43 (1) (2006) 55-60.

[97] M. A. Riaz, R. Poupardin, S. Reynaud, C. Strode, H. Ranson,J. P. David, Impact of glyphosate and benzo [a] pyrene on the tolerance of mosquito larvae to chemical insecticides. Role of detoxification genes in response to xenobiotics, Aquat Toxicol 93 (1) (2009) 61-69.

[98] M. A. Riaz, A. Chandor-Proust, C. Dauphin-Villemant, R. Poupardin, C. M. Jones, C. Strode, M. Regent-Kloeckner, J. P. David,S. Reynaud, Molecular mechanisms associated with increased tolerance to the neonicotinoid insecticide imidacloprid in the dengue vector *Aedes aegypti*, Aquat Toxicol 126 (2013) 326-337.

[99] M. A. I. Ahmed,F. Matsumura, Synergistic actions of formamidine insecticides on the activity of pyrethroids and neonicotinoids against *Aedes aegypti* (Diptera: Culicidae), J Med Entomol 49 (6) (2012) 1405-1410.

[100] H. Liu, E. W. Cupp, A. G. Guo,N. N. Liu, Insecticide resistance in Alabama and Florida mosquito strains of *Aedes albopictus*, J Med Entomol 41 (5) (2004) 946-952.

[101] A. Yokoyama, K. Ohtsu, T. Iwafune, T. Nagai, S. Ishihara, Y. Kobara, T. Horio,S. Endo, A useful new insecticide bioassay using first-instar larvae of a net-spinning caddisfly, *Cheumatopsyche brevilineata* (Trichoptera: Hydropsychidae), J Pestic Sci 34 (1) (2009) 13-20.

[102] E. Maloney, C. Morrissey, J. Headley, K. Peru,K. Liber, Can chronic exposure to imidacloprid, clothianidin, and thiamethoxam mixtures exert greater than additive toxicity in *Chironomus dilutus*?, Ecotoxicol. Environ. Saf. 156 (2018) 354-365.

[103] M. Raby, X. M. Zhao, C. Y. Hao, D. G. Poirier,P. K. Sibley, Chronic toxicity of 6 neonicotinoid insecticides to *Chironomus dilutus* and *Neocloeon triangulifer*, Environ. Toxicol. Chem. 37 (10) (2018) 2727-2739.

[104] M. C. Cavallaro, C. A. Morrissey, J. V. Headley, K. M. Peru,K. Liber, Comparative chronic toxicity of imidacloprid, clothianidin, and thiamethoxam to *Chironomus dilutus* and estimation of toxic equivalency factors, Environ. Toxicol. Chem. 36 (2) (2017) 372-382.

[105] E. M. Maloney, H. Sykes, C. Morrissey, K. M. Peru, J. V. Headley,K. Liber, Comparing the acute toxicity of imidacloprid with alternative systemic insecticides in the aquatic insect *Chironomus dilutus*., Environ. Toxicol. Chem. 39 (3) (2020) 587-594.

[106] E. M. Maloney, C. A. Morrissey, J. V. Headley, K. M. Peru,K. Liber, Cumulative toxicity of neonicotinoid insecticide mixtures to *Chironomus dilutus* under acute exposure scenarios, Environ. Toxicol. Chem. 36 (11) (2017) 3091-3101.

[107] H. M. K. LeBlanc, J. M. Culp, D. J. Baird, A. C. Alexander,A. J. Cessna, Single versus combined lethal effects of three agricultural insecticides on larvae of the freshwater insect *Chironomus dilutus*, Arch Environ Con Tox 63 (3) (2012) 378-390.

[108] F. H. Wei, D. L. Wang, H. Z. Li, P. Xia, Y. Ran,J. You, Toxicogenomics provides insights to toxicity pathways of neonicotinoids to aquatic insect, *Chironomus dilutus*, Environ. Pollut. 260 (2020) 114011.

[109] N. N. Chandran, D. Fojtova, L. Blahova, E. Rozmankova,L. Blaha, Acute and (sub)chronic toxicity of the neonicotinoid imidacloprid on *Chironomus riparius*, Chemosphere 209 (2018) 568-577.

[110] H. M. V. S. Azevedo-Pereira, M. F. L. Lemos,A. M. V. M. Soares, Behaviour and growth of *Chironomus riparius* meigen (Diptera: Chironomidae) under imidacloprid pulse and constant exposure scenarios, Water Air Soil Poll 219 (2011) 215-224.

[111] C. Scherer, R. Wolf, J. Volker, F. Stock, N. Brennhold, G. Reifferscheid,M. Wagner, Toxicity of microplastics and natural particles in the freshwater dipteran *Chironomus riparius*: Same same but different?, Sci. Total Environ. 711 (2020) 134604.

[112] M. M. Stevens, A. Ali, S. Helliwell, L. J. Schiller,S. Hansen, Comparison of two bioassay techniques for assessing the acute toxicity of pesticides to chironomid larvae (Diptera: Chironomidae), J Am Mosquito Contr 18 (2) (2002) 119-125.

[113] S. J. Macaulay, D. B. Buchwalter,C. D. Matthaei, Water temperature interacts with the insecticide imidacloprid to alter acute lethal and sublethal toxicity to mayfly larvae, New Zeal J Mar Fresh 54 (1) (2020) 115-130.

[114] R. F. Shoukat, S. Freed, K. W. Ahmad,Ateeq-ur-Rehman, Assessment of binary mixtures of entomopathogenic fungi and chemical insecticides on biological parameters of *Culex pipiens* (Diptera: Culicidae) under laboratory and field conditions, Pak J Zool 50 (1) (2018) 299-309.

[115] M. A. I. Ahmed,A. A. Othman, Piperonyl butoxide enhances the insecticidal toxicity of nanoformulation of imidacloprid on *Culex pipiens* (Diptera: Culicidae) mosquito, Vector-Borne Zoonotic Dis. 20 (2) (2020) 134-142.

[116] Y. Al Naggar, J. P. Giesy,S. El Kholy, Sublethal effects of chronic exposure to chlorpyrifos or imidacloprid insecticides or their binary mixtures on *Culex pipiens* mosquitoes, Physiol. Entomol. 44 (2) (2019) 123-132.

[117] H. Liu, E. W. Cupp, K. M. Micher, A. Guo,N. Liu, Insecticide resistance and cross-resistance in Alabama and Florida strains of *Culex quinquefasciatus*, J Med Entomol 41 (3) (2004) 408-13.

[118] S. Bhan, L. Mohan,C. N. Srivastava, Relative larvicidal potentiality of nano-encapsulated temephos and imidacloprid against *Culex quinquefasciatus*, J Asia-Pac Entomol 17 (4) (2014) 787-791.

[119] S. J. Macaulay, K. J. Hageman, R. E. Alumbaugh, S. M. Lyons, J. J. Piggott,C. D. Matthaei, Chronic toxicities of neonicotinoids to nymphs of the common New Zealand mayfly *Deleatidium* spp., Environ. Toxicol. Chem. 38 (11) (2019) 2459-2471.

[120] A. J. Bartlett, A. M. Hedges, K. D. Intini, L. R. Brown, F. J. Maisonneuve, S. A. Robinson, P. L. Gillis,S. R. de Solla, Lethal and sublethal toxicity of neonicotinoid and butenolide insecticides to the mayfly, *Hexagenia* spp., Environ. Pollut. 238 (2018) 63-75.

[121] H. M. LeBlanc. Single and combined effects of the agricultural insecticides chlorpyrifos, imidacloprid and dimethoate on freshwater insect larvae. Library and Archives Canada= Bibliothèque et Archives Canada, Ottawa, 2013.

[122] A. A. Camp,D. B. Buchwalter, Can't take the heat: Temperature-enhanced toxicity in the mayfly *Isonychia bicolor* exposed to the neonicotinoid insecticide imidacloprid, Aquat Toxicol 178 (2016) 49-57.

[123] D. P. Kreutzweiser, K. P. Good, D. T. Chartrand, T. A. Scarr,D. G. Thompson, Toxicity of the systemic insecticide, imidacloprid, to forest stream insects and microbial communities, B Environ Contam Tox 80 (3) (2008) 211-214.

[124] J. P. Overmyer, B. N. Mason,K. L. Armbrust, Acute toxicity of imidacloprid and fipronil to a nontarget aquatic insect, *Simulium vittatum* Zetterstedt cytospecies IS-7, B Environ Contam Tox 74 (5) (2005) 872-879.

[125] L. Pourzahedi, M. Pandorf, D. Ravikumar, J. B. Zimmerman, T. P. Seager, T. L. Theis, P. Westerhoff, L. M. Gilbertson,G. V. Lowry, Life cycle considerations of nano-enabled agrochemicals: Are today's tools up to the task?, Environ. Sci.: Nano 5 (5) (2018) 1057–1069.

[126] A. M. Sadaria, S. D. Supowit,R. U. Halden, Mass balance assessment for six neonicotinoid insecticides during conventional wastewater and wetland treatment: Nationwide reconnaissance in United States wastewater, Environ. Sci. Technol. 50 (12) (2016) 6199-6206.

[127] L. P. Burkhard, Estimating dissolved organic carbon partition coefficients for nonionic organic chemicals, Environ. Sci. Technol. 34 (22) (2000) 4663-4668.

[128] F. Soheilifard, A. Marzban, M. G. Raini, M. Taki,R. van Zelm, Chemical footprint of pesticides used in citrus orchards based on canopy deposition and off-target losses, Sci. Total Environ. 732 (2020) 139118.

[129] C. Renaud-Gentie, T. Dijkman, A. Bjorn,M. Birkved, Pesticide emission modelling and freshwater ecotoxicity assessment for grapevine LCA: Adaptation of PestLCI 2.0 to viticulture, Int. J. Life Cycle Assess. 20 (11) (2015) 1528-1543.

[130] C. Gentil, C. Basset-Mens, S. Manteaux, C. Mottes, E. Maillard, Y. Biard,P. Fantke, Coupling pesticide emission and toxicity characterization models for LCA: Application to open-field tomato production in Martinique, J. Cleaner Prod. 277 (2020) 124099.

[131] N. Peña, M. T. Knudsen, P. Fantke, A. Antón,J. E. Hermansen, Freshwater ecotoxicity assessment of pesticide use in crop production: Testing the influence of modeling choices, J. Cleaner Prod. 209 (2019) 1332–1341.

[132] A. Rahman, S. J. Kang, S. McGinnis,P. J. Vikesland, Life cycle impact assessment of iron oxide (Fe_3_O_4_/γ-Fe_2_O_3_) nanoparticle synthesis routes, Acs Sustain Chem Eng 10 (10) (2022) 3155-3165.

[133] F. Wu, Z. Zhou,A. L. Hicks, Life cycle impact of titanium dioxide nanoparticle synthesis through physical, chemical, and biological routes, Environ. Sci. Technol. 53 (8) (2019) 4078–4087.

[134] L. Pourzahedi,M. J. Eckelman, Comparative life cycle assessment of silver nanoparticle synthesis routes, Environ. Sci.: Nano 2 (4) (2015) 361–369.

[135] S. Temizel-Sekeryan,A. L. Hicks, Global environmental impacts of silver nanoparticle production methods supported by life cycle assessment, Resour., Conserv. Recycl. 156 (2020) 104676.

[136] S. Temizel-Sekeryan, F. Wu,A. L. Hicks, Global scale life cycle environmental impacts of single- and multi-walled carbon nanotube synthesis processes, Int. J. Life Cycle Assess. 26 (4) (2021) 656-672.
